# Development of potent isoflavone-based formyl peptide receptor 1 (FPR1) antagonists and their effects in gastric cancer cell models

**DOI:** 10.1016/j.ejmech.2023.115854

**Published:** 2023-10-04

**Authors:** Fabio Francavilla, Federica Sarcina, Igor A. Schepetkin, Lilya N. Kirpotina, Marialessandra Contino, Annalisa Schirizzi, Giampiero De Leonardis, Andrei I. Khlebnikov, Rosalba D’Alessandro, Mark T. Quinn, Enza Lacivita, Marcello Leopoldo

**Affiliations:** a Dipartimento di Farmacia-Scienze del Farmaco, Universitá degli Studi di Bari Aldo Moro, via Orabona 4, 70125, Bari, Italy; b Department of Microbiology and Cell Biology, Montana State University, Bozeman, MT, 59717, USA; c Laboratory of Experimental Oncology, National Institute of Gastroenterology - IRCCS “Saverio de Bellis”, Research Hospital, 70013, Castellana Grotte (BA), Italy; d Kizhner Research Center, Tomsk Polytechnic University, Tomsk, 634050, Russia

**Keywords:** FPR1, Gastric cancer, 4*H*-chromen-2-one derivatives, Docking studies, Cell growth

## Abstract

Formyl peptide receptor-1 (FPR1) is a G protein-coupled chemoattractant receptor that plays a crucial role in the trafficking of leukocytes into the sites of bacterial infection and inflammation. Recently, FPR1 was shown to be expressed in different types of tumor cells and could play a significant role in tumor growth and invasiveness. Starting from the previously reported FPR1 antagonist **4**, we have designed a new series of 4*H*-chromen-2-one derivatives that exhibited a substantial increase in FPR1 antagonist potency. Docking studies identified the key interactions for antagonist activity. The most potent compounds in this series (**24a** and **25b**) were selected to study the effects of the pharmacological blockade of FPR1 in NCl–N87 and AGS gastric cancer cells. Both compounds potently inhibited cell growth through a combined effect on cell proliferation and apoptosis and reduced cell migration, while inducing an increase in angiogenesis, thus suggesting that FPR1 could play a dual role as oncogene and onco-suppressor.

## 1. Introduction

Formyl peptide receptors (FPRs) are G protein-coupled receptors (GPCRs), belonging to the family of pattern recognition receptors that regulate innate immune responses [1]. FPRs play important roles as sensors of pathogen- and host-derived products and recruit leukocytes to the sites of infection where these cells exert microbicidal functions and clear cellular debris [2,3]. In humans, three FPR isoforms (FPR1, FPR2, and FPR3) exist and share different degrees of sequence similarity [1,4]. FPR1 was the first named member of the family because of the high affinity for bacterial or mitochondrial formylated peptides, such as *N*-formyl-methionyl-leucyl-phenylalanine (*f*MLF) produced by Gram-negative bacteria. FPR2 is a promiscuous receptor binding to *f*MLF with lower affinity but also to various viral, bacterial, host-derived, and synthetic peptides. FPRs are mainly expressed on phagocytic leukocytes, such as monocytes, neutrophils, and macrophages, but are also found in some non-hematopoietic cells, including epithelial cells, endothelial cells, neurons, and astrocytes [1,5,6]. FPRs predominantly govern the pro-inflammatory response, resulting in chemotaxis, degranulation, and oxidative burst during infection [7,8]. In addition, FPRs promote wound healing and maintain gut mucosal integrity. On the other hand, FPRs have been implicated in cancer progression for abnormal growth, survival, and invasion due to their aberrant expression in several cancers, including malignant glioblastoma, breast cancer, colon cancer, and gastric cancer [7–11].

Gastric cancer (GC) is the fifth most common malignancy and the third leading cause of cancer-related deaths worldwide [12]. Despite the remarkable surgical and therapeutic achievements, the overall 5-year survival rate of GC patients remains low due to the advanced stage of diagnosis and the malignant nature of invasion and metastasis of the disease [13]. All members of the FPR family have been detected in human GC cells, but only the function of FPR1 and FPR2 has been investigated [14]. The involvement of FPR1 in GC progression is controversial. FPR1 is highly expressed in GC tissues, particularly in stage IV disease, and its expression has been associated with deeper invasion depth and poorer clinical outcomes in patients. High FPR1 expression is also related to lower overall survival rates; thus, FPR1 has been proposed as a prognostic marker for poorer survival in GC patients [15]. Otani et al. reported that a specific FPR1 polymorphism, which reduces FPR1 activity, is positively associated with GC risk [16]. On the other hand, it has been proposed that FPR1 may act as a tumor suppressor by inhibiting angiogenesis in GC xenografts because silencing of FPR1 augmented vessel density in tumor stroma [17]. In addition, the pharmacological modulation of FPR1 activity through ALOX5/15 expression and production of pro-resolving mediators resolvin D1 and lipoxin B4, which transduce anti-inflammatory signals, reduced the rate of GC progression by inhibiting inflammatory and angiogenic processes in the tumor microenvironment [18]. The effect of FPR1 pharmacological blockade on GC progression has never been studied.

**Fig. 1.**
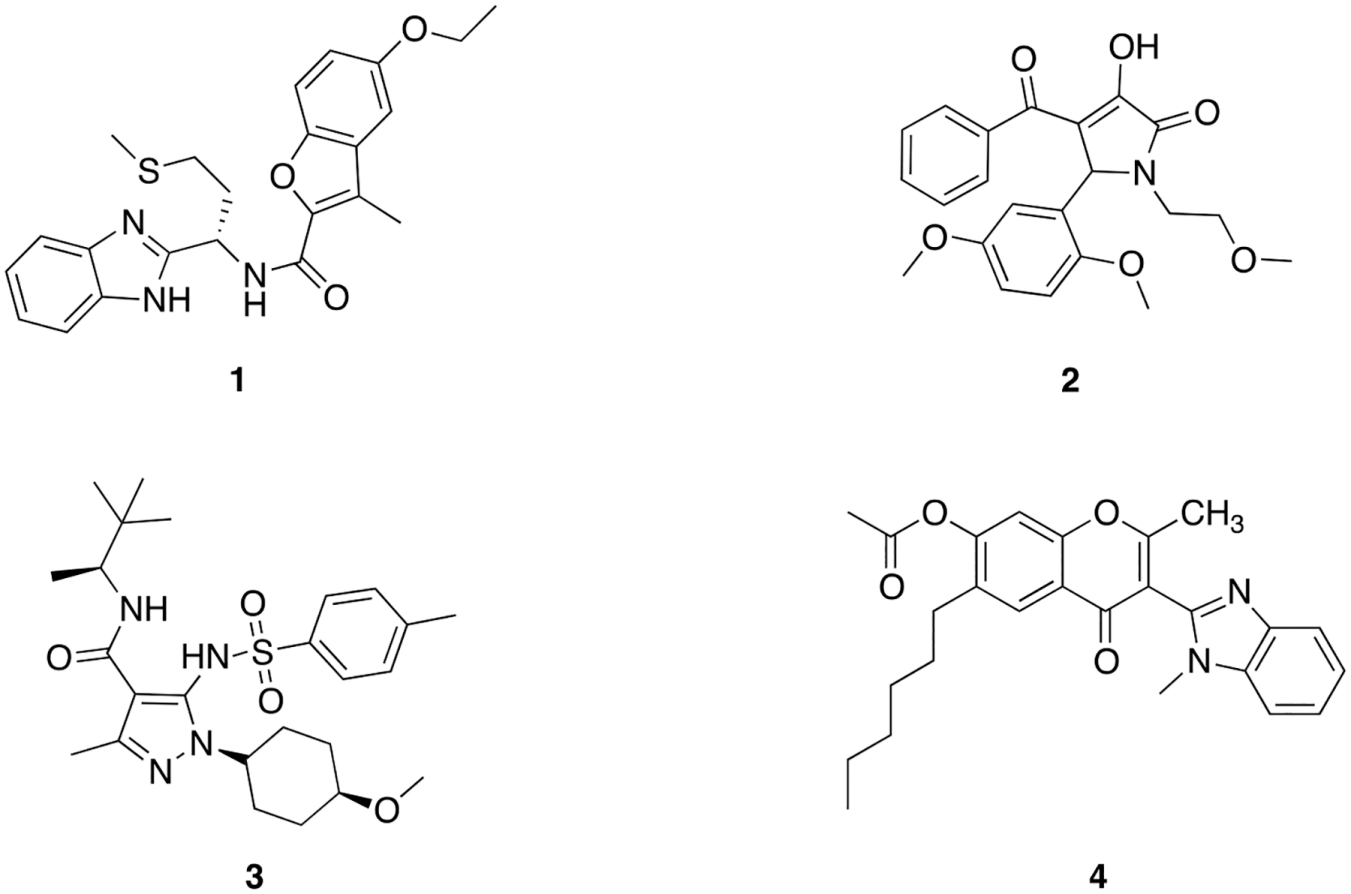
Chemical structures of small-molecule FPR1 antagonists.

The fungal hydrophobic cyclic peptides cyclosporines A and H are among the most potent FPR1 antagonists available [19,20]. In addition, several “small-molecule” FPR1 antagonists exhibiting a wide range of antagonist potency and selectivity towards FPR2 subtype have been identified so far, including the methionine-derived benzimidazoles (compound **1**) [21], the 1*H*-pyrrol-2(5*H*)-one derivatives such as compound **2** [22], and the pyrazole-4-carboxamides like compound **3** [23], (Fig. 1). The latter compound, also known as AZ2158, has been reported to potently inhibit *f*MLF-induced chemotaxis and NADPH oxidase activation in human neutrophils [24]. We have contributed to the field with the identification of a series of 4*H*-chromen-4-one derivatives exemplified by compound **4** (Fig. 1), which exhibited FPR1 antagonist activity in the submicromolar range (IC_50_ = 0.31 μM) and was selective for FPR2 and FPR3 subtypes and the chemokine receptor CXCR1 [25]. Compound **4** also inhibited *f*MLF-induced chemotaxis in human neutrophils (IC_50_ = 0.024 μM) [25], thus suggesting that 4*H*-chromen-4-one scaffold could be a good starting point to obtain potent and selective FPR1 antagonists. Keeping on our studies on FPR1 antagonists, we report on the optimization of the 4*H*-chromen-4-one scaffold. The most potent compounds were also used to study the role of FPR1 blockade on cell vitality and invasiveness of the gastric adenocarcinoma cell lines AGS and NCl–N87.

## 2. Study design

**Fig. 2.**
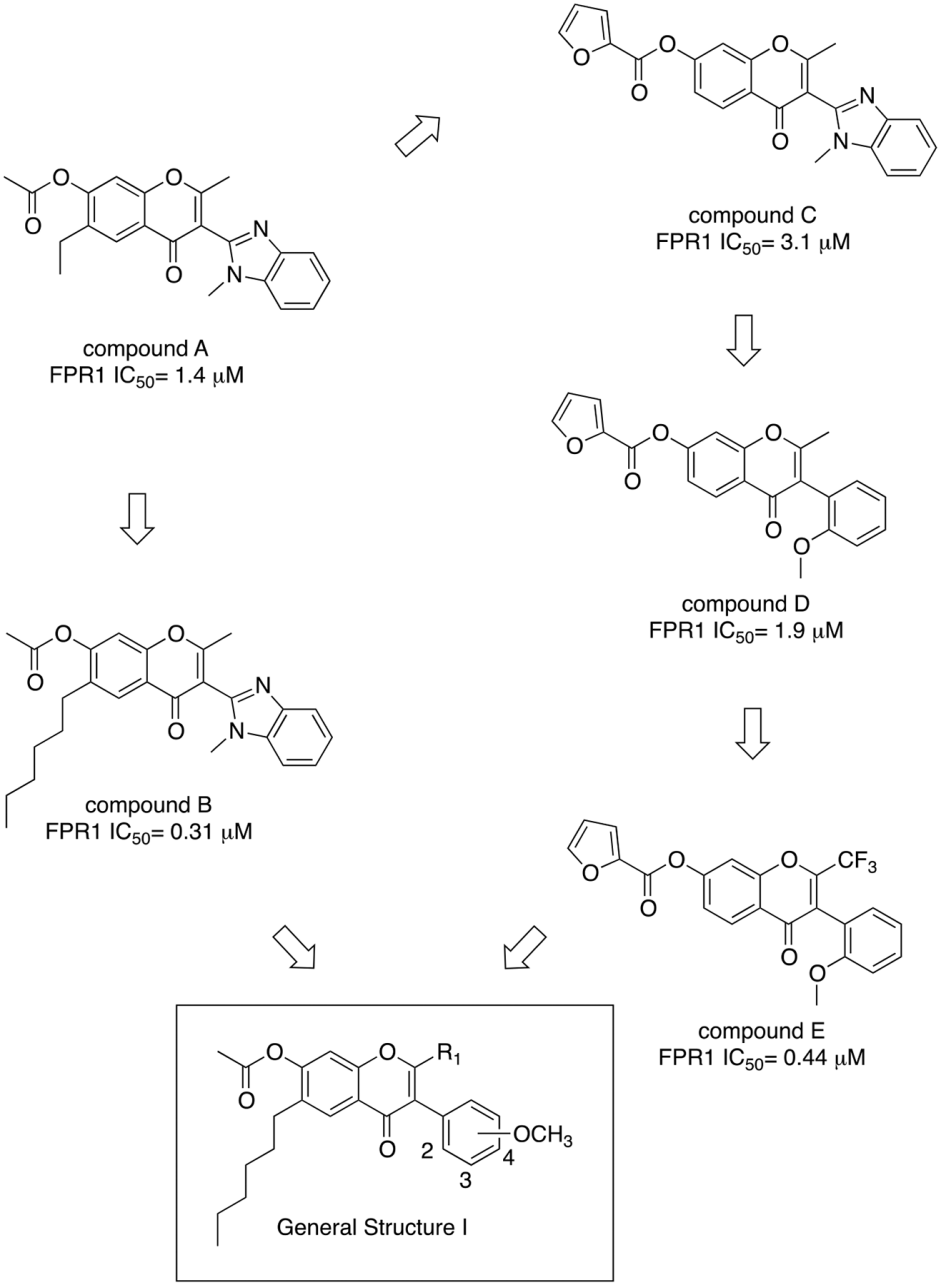
Graphical representation of the design of the new isoflavone FPR1 antagonists.

Previously, a series of ninety-six 4*H*-chromen-4-one derivatives were investigated as FPR1 antagonists, of which twenty-six were found to be active (0.31 μM < IC_50_ < 24.7 μM) [25]. In spite of the large number of analogs evaluated, the study did not provide a detailed description of the structure-activity relationships (SARs) of this set of compounds. Neverthless, it provided clues for further improvements in FPR1 antagonist potency. For example, the change from ethyl to hexyl in the 6-position of the chromone ring in compound A led to compound B with a 3-fold increase in potency (Fig. 2). A gain in potency was also observed when the benzimidazole ring (compound C) was replaced by a 2-methoxyphenyl ring (compound D) and when the 2-methyl group of compound D was replaced by trifluoromethyl group (compound E). Therefore, we combined the above-mentioned structural variations and designed the compounds **24a-c** and **25a-c**, described by General Structure I (Fig. 2). Once the key SARs of these compounds were assessed, we addressed the potential metabolic liability of the acetoxy function in the 7-position of the chromone ring by applying different approaches (compounds **30**, **35a-c**, **37a-c**, **38**, and **39**, Table 1).

## 3. Chemistry

**Scheme 1.**
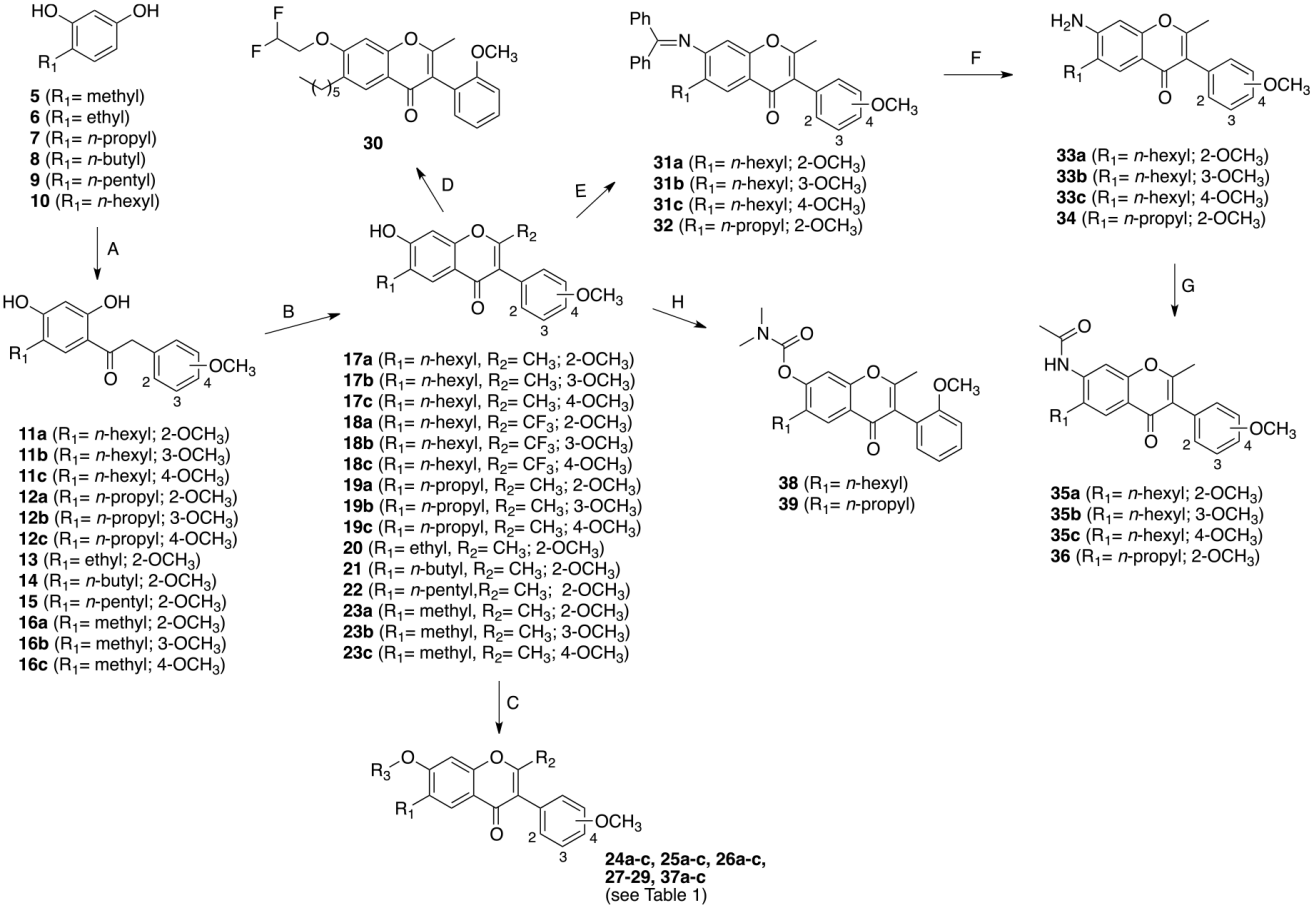
Synthesis of Target Compounds^a^ ^*a*^Reagents and Conditions: (A) methoxyphenyl acetyl chlorides, AlCl_3_, anhydrous 1,2-dichlorobenzene, 70 °C, 2h, 10–57% yield; (B) acetic anhydride, anhydrous DMF, 115 °C, 2 h or trifluoroacetic anhydride, anhydrous pyridine, r.t, overnight, yield 10–85%; (C) acetic anhydride, anhydrous pyridine, r.t., 4 days, or heptanoyl chloride, DMAP, Et_3_N, anhydrous DMF, r.t., 2 h, 14–71% yield; (D) 2,2-difluoroethyl methanesulfonate, K_2_CO_3_, anhydrous DMF, 90 °C, 16 h, 32% yield; (E) *i*: trifluoromethanesulfonic anhydride, DMAP, pyridine, anhydrous CH_2_Cl_2_, r.t., 4 h; *ii*: benzophenone imine, Pd(OAc)_2_, Cs_2_CO_3_, BINAP, anhydrous THF, 80 °C, 22 h, 15–25%; (F) 2 M HCl, THF, r.t., 1 h; (G) acetyl chloride, Et_3_N, CH_2_Cl_2_, r.t., 5 h, 53–90% yield; (H) *N,N*-dimethylcarbamoylcarbonate, K_2_CO_3_, acetonitrile, reflux, 5 h, 35–42% yield.

Synthesis of the target compounds is depicted in Scheme 1. A Friedel–Crafts reaction between alkyl benzene-1,3-dioles **5–10**, prepared according to the literature or commercially available [26–28], and methoxyphenylacetyl chlorides resulted in ketones **11a-c-12a-c**, **13–15**, and **16a-c**, which were cyclized with acetic anhydride or trifluoroacetic anhydride to obtain the key hydroxy chromones **17a-c-19a-c**, **20–22**, and **23a-c**. Target compounds featuring the acetoxy substituent were obtained from the corresponding phenol by reaction with acetyl chloride (**24a-c-26a-c,** and **27–29**). Derivative **30** was obtained from phenol **17a** by reaction with 2,2-difluoroethylmethansulfonate. The final acetamide derivatives **35a-c** and **36** were obtained as follows: phenols **17a-c** and **19a** were transformed into the corresponding triflate derivatives, which reacted with the benzophenone imine to give the intermediates **31a-c** and **32**, that by subsequent hydrolysis resulted in amines **33a-c** and **34**. Reaction of the latter compounds with acetyl chloride resulted in the desired target compounds **31a-c** and **32**. Target compounds featuring the heptanoyl substituent were obtained by reacting phenols **23a-c** with heptanoyl chloride (**37a-c**). Carbamate derivatives **38** and **39** were prepared by reacting phenol **17a** or **19a** with *N,N*-dimethylcarbamoylcarbonate, respectively.

## 4. Functional activity of the target compounds

Antagonist activity of the target compounds for FPR1 and FPR2 was assessed by measuring their ability to inhibit Ca^2+^ mobilization induced by the standard agonists *f*MLF and WKYMV, respectively, in HL-60 cells stably transfected with human FPR1 or FPR2 and was expressed as IC_50_.

Exploration of the SAR around compound B started by introducing a methoxyphenyl ring in place of the benzimidazole ring and replacing the 2-methyl with a 2-trifluoromethyl group. We found that the CF_3_- derivatives (**25a-c**) were more potent than the corresponding CH_3_- derivatives (**24a-c**) and that the position of the methoxy group on the 3-phenyl ring influenced the potency, with rank order 2-OCH_3_ > 3-OCH_3_ > 4-OCH_3_ (Table 1). Next, we evaluated the effect of the gradual shortening of the hexyl chain in compounds **24a** (pentyl **29**, butyl **28**, propyl **26a**, ethyl **27**). We observed a loss of potency in all cases but the propyl derivative **26a** (IC_50_ = 0.09 μM) (Table 1). These potency data prompted us further to evaluate the 3- and 4-methoxy isomers of **26a** (compounds **26b-c**). As noted for the corresponding hexyl analogs, the 2-methoxyphenyl derivative featured the highest FPR1 antagonist potency (compound **26a**, Table 1).

Collectively, the FPR1 antagonist activity data indicated that the design strategy was successful as we identified various FPR1 antagonists that were more potent than the starting points compounds A–E. Compounds **24a** and **25a** were the most potent of the set (IC_50_ = 0.07 and 0.025 μM, respectively).

Next, we addressed the presence of the acetoxy group in the structure of these FPR1 antagonists as a source of limited metabolic stability. We assessed metabolic stability of compound **24a** and **25a** in rat liver microsomes using a NADPH-regenerating system. After 30 min incubation, we could not detect the presence of the parent compounds in the incubation mixture, confirming that the ester function is readily hydrolyzed. Thus, we replaced the acetoxy group of compounds **24a-c**, and **26a** with an acetylamino group (compounds **35a-c** and **36**, respectively), which is much more stable toward the hydrolysis. Unfortunately, all the acetylamino derivatives were inactive. Following the same approach, we replaced the acetoxy group of **24a** and **26a** with a carbamoyloxy group (**38** and **39**, respectively) but also found practically inactive compounds. We also replaced the acetoxy group of compound **24a** with the 2,2-difluoroethoxy substituent, obtaining compound **30**, which was inactive at FPR1. Lastly, we reasoned whether exchanging the size of the substituents in positions 6- and 7- of compounds **24a-c** might be tolerated for FPR1 antagonist activity and provide more stable compounds. Thus, we designed derivatives **37a-c** featuring a *n-*hexanoyloxy group in the 6- position and a methyl in the 7- position. Again, this modification practically abolished the FPR1 antagonist activity. At this point, we evaluated compounds **30**, **35a**, **36**, **37a**, and **39** for their metabolic stability. We could not detect the parent compound in the incubation mixture of compound **37a**, thus suggesting that the introduction of a bulky carboxylic acid did not improve the stability. For the other compounds the percentages of recovery of the parent compounds after 30 min incubation were as follows: 18% for compound **30**; 14% for compound **35a**; 15% for compound **36**; 10% for compound **39**. These data suggest that the metabolic stability of these derivatives does not depend only on the acetoxy group, but likely from other soft spots in the 4*H*-chromen-2-one scaffold. Further studies are thus needed to combine high FPR1 antagonist potency and metabolic stability.

## 5. Molecular modeling

**Fig. 3.**
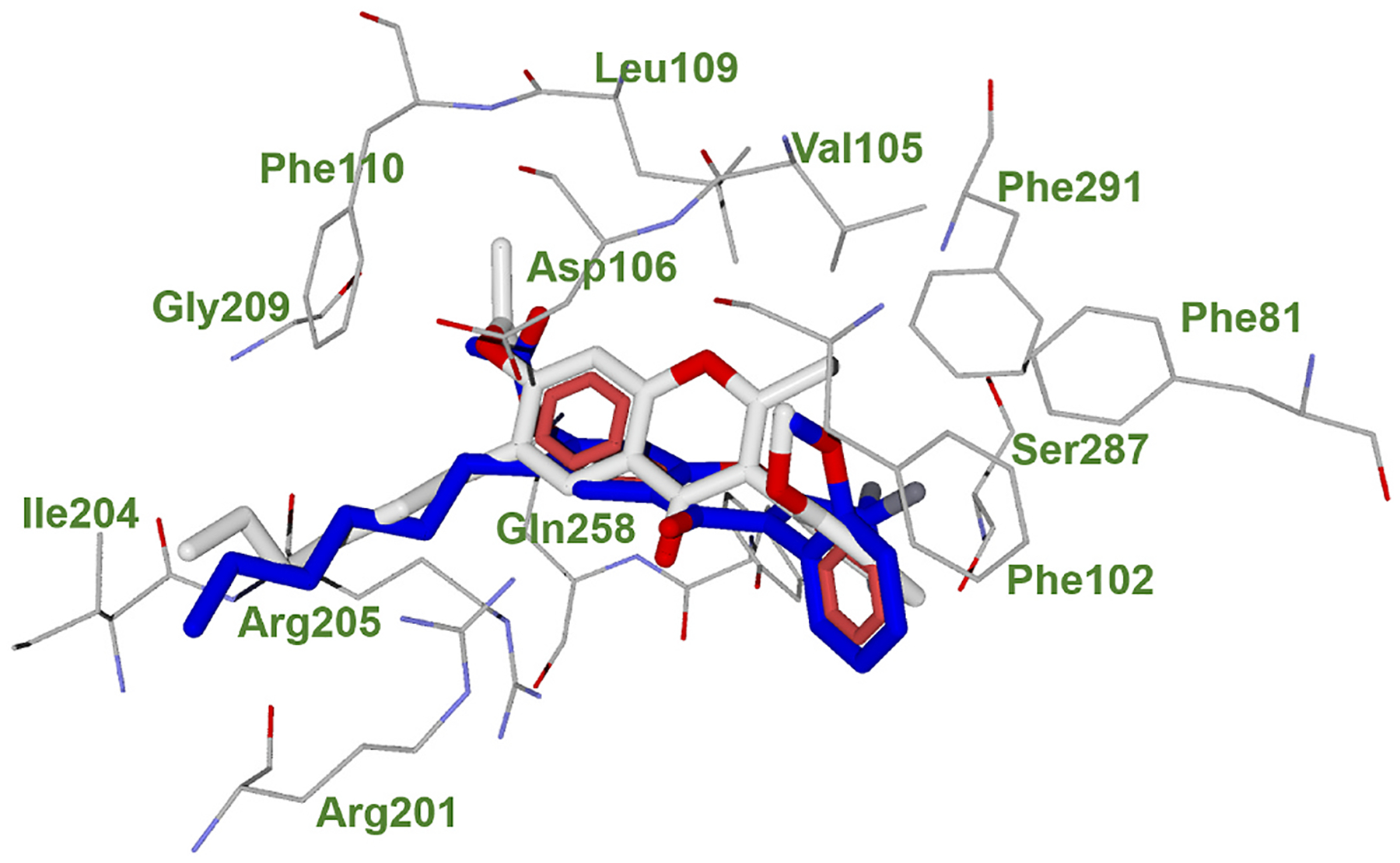
Superimposed docking poses of compounds **24a** (grey) and **25a** (blue). Residues within 3 Å from **25a** are shown.

While the study was in progress, cryo-EM structures of the FPR1-Gi complex bound to the *S. aureus*-derived peptide agonist *f*Met-Ile-Phe-Leu (fMIFL) and *f*MLF were solved and published [29]. These cryo-EM structures identified the ligand binding pocket of FPR1, which was surrounded by transmembrane (TM) helices 2,3,5,6, with minor involvement of TM7. In particular, *N*-formyl-methionine of *f*MIFL forms hydrogen bond contacts with the charged residues D106, R201, and R205 and is surrounded by a hydrophobic pocket formed by L109, F110, V113, W254, and Q258. The Ile of *f*MIFL is surrounded by the hydrophobic residues F81, V105, and F291. The aromatic ring of Phe in *f*MIFL phenylalanine forms hydrophobic contacts with T265, while the carboxy-terminal leucine is surrounded by a hydrophobic cap formed by R84, F102, and F178 [29]. These findings confirmed previous site-directed mutagenesis studies that identified D106, R201, and R205 as key residues for the binding of FPR1 agonists. Therefore, we exploited these pieces of information to reveal insights into the binding mode of the FPR1 antagonists included in the present study. First, we performed a molecular docking study on the most potent FPR1 antagonists identified in this study, **24a** and **25a** (Fig. 3). We found that both compounds partly occupied the binding site of *f*MIFL (Fig. S1, Supporting Information) by establishing a hydrogen bond between the carbonyl group of the chromone ring and R201 but not establishing contacts with W254 as does *f*MIFL. The substituents of the **24a** and **25a** chromone rings establish contact with P81, Y257, S287, and P291 residues. Compound **25a** forms an additional hydrogen bond between the oxygen atom of the chromone ring and Y257 (Fig. 3). The hexyl chain of both compounds points toward a small hydrophobic subpocket located between TM5 and TM4, which is surrounded by residues L156, P159, and I204. Interestingly, *f*MIFL has no contact with this subpocket, suggesting that such a difference may account for the different functional activities of **24a** and **25a** compared to *f*MIFL. In both compounds **24a** and **25a**, the phenyl ring is orthogonal to the plane of the chromen-2-one moiety, most likely because of the steric repulsion between the methoxy group and the 2-substituent of the chromen-2-one nucleus.

**Fig. 4.**
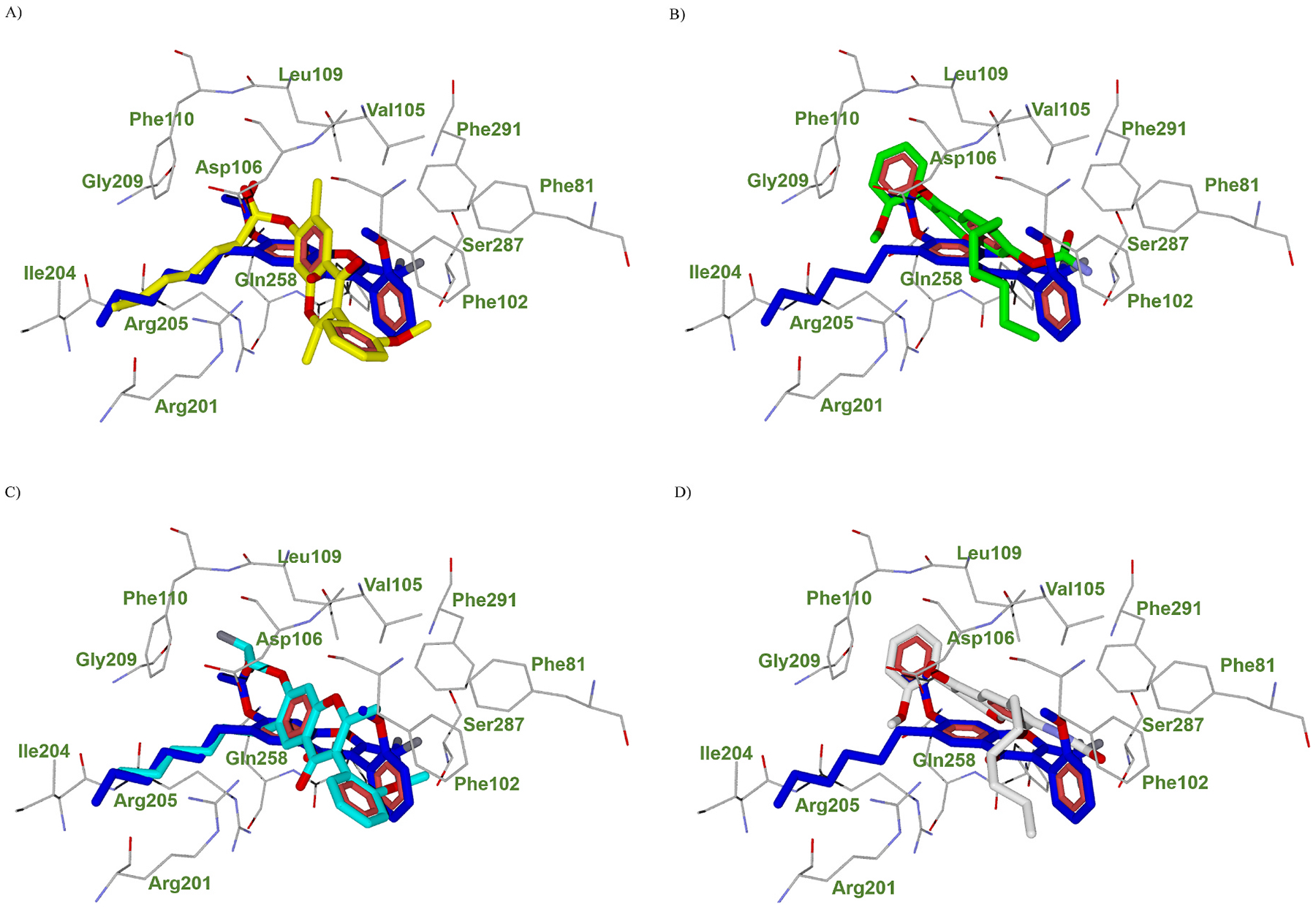
Pairwise superimposition of the docking poses of **37a** (yellow) (panel A); **38** (green) (panel B); **30** (light blue) (panel C); **35a** (grey) (panel D) on the docking pose of **25a** (blue). Residues within 3 Å from **25a** are shown.

We next performed docking studies on four inactive compounds, i.e., **30**, **35a**, **37a**, and **39**. Pairwise superimpositions of the docking pose of these compounds with **25a** are presented in Fig. 4. The docking poses of **35a** and **37a** are similar but substantially different from the **25a** docking pose, with the hexyl chain and the 2-methoxyphenyl ring of **35a** and **37a** completely mismatched compared to **25a**. It is tempting to attribute the different binding modes to the presence in **35a** and **37a** of the hydrogen bond donor NH (which is absent in **25a**), even though the docking study did not provide evidence for a specific contact between the substituent in the 7-position of **35a** and **37a** with FPR1 binding site. As for compounds **30** and **37a**, docking studies indicated that the hexyl substituent established contacts with the FPR1 binding site similar to that of the hexyl substituent of compound **25a**. At the same time, the other moieties of **30** and **37a** do not superimpose with the docking pose of **25a**.

Overall, our design strategy proved to be successful, as the replacement of the benzimidazole moiety of compound **4** with the 2-methoxy substituted phenyl ring led to an improvement of FPR1 antagonist potency (compound **24 a-c**). Replacement of the 2-CH_3_ substituent with a CF_3_ further improved the potency, leading to compound **25a**, the most potent compound within this series. The 7-acetoxy function proved to be important for the interaction of the molecule with the receptor because its replacement with other substituents led to a loss of activity. Although docking studies did not support an interaction of the acetoxy group within the binding site, it can be argued that the presence of this substituent is essential for an efficient orientation of the molecule within the binding site. On the other hand, the presence of this structural feature negatively contributed to the limited metabolic stability of the molecule.

## 6. Evaluation of the effect of FPR1 antagonism in gastric cancer cells

**Fig. 5.**
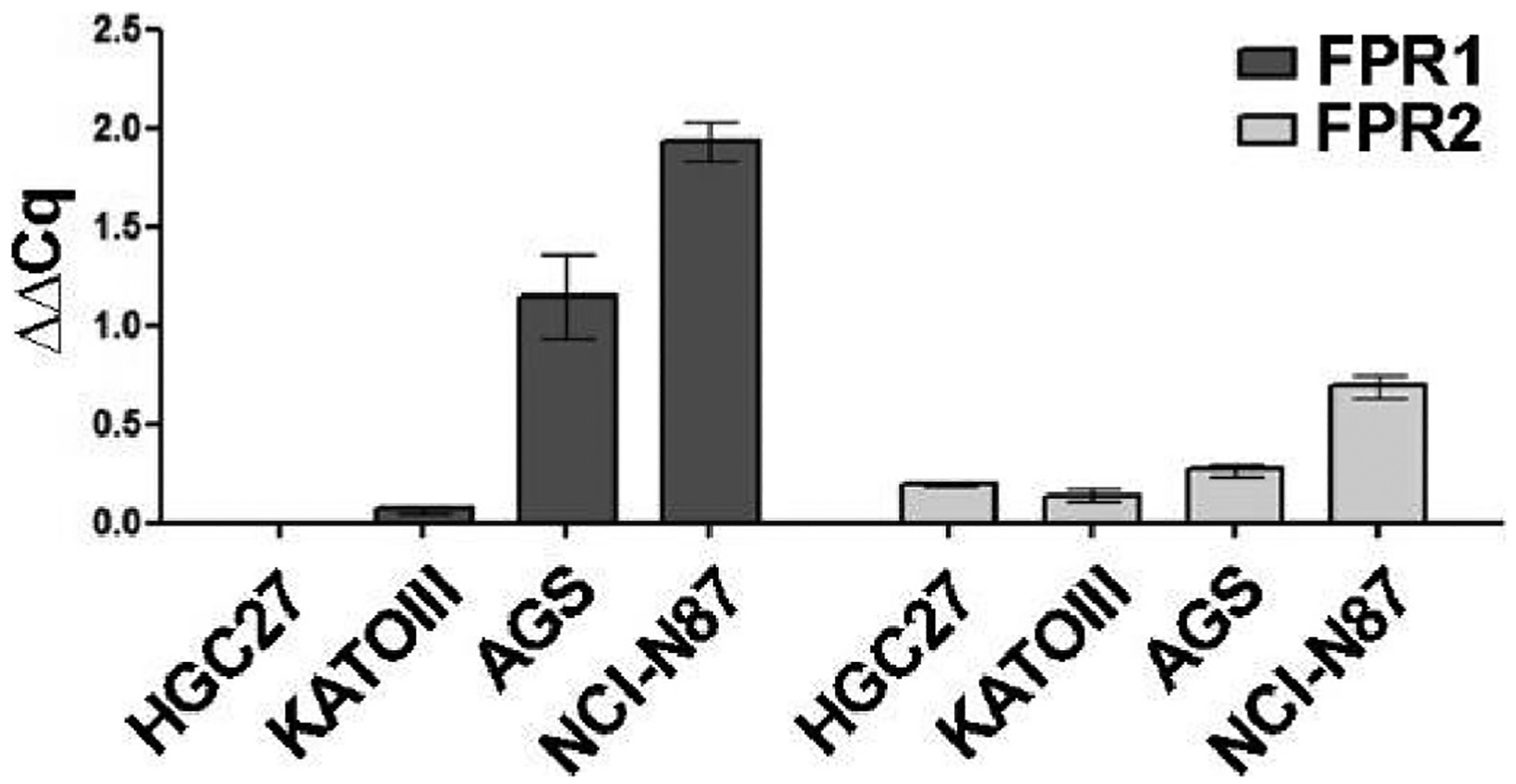
FPR1 and FPR2 RNA expression level in GC cell lines. Real-time PCR experiments in HGC27, KATOIII, AGS and NCl–N87 cell lines with specific primers for FPR1 and FPR2 genes. The mRNA expression was normalized on the GAPDH housekeeping gene. Data were mean ± SD (n = 3).

Literature data indicate that silencing FPR1 in GC cells through RNA interference impairs cell growth and migration while increasing proangiogenic response in GC epithelial cells [17]. We decided to study, for the first time, the effect of pharmacological blockade of FPR1 on cell proliferation, cell migration, and angiogenesis in GC by using our antagonists **24a** and **25a**. We first assessed the expression levels of FPR1 and FPR2 mRNA in four GC cell lines, i.e., HGC27, derived from human gastric carcinoma, KATOIII, AGS, and NCl–N87, derived from human gastric adenocarcinoma. We found that FPR1 had a high expression level in NCl–N87 and AGS cells, a low expression level in KATOIII cells, and was absent in the HGC27 cell line. On the other hand, FPR2 was expressed in all lines, with notably higher levels in AGS and NCl–N87 cells (Fig. 5). We selected AGS and NCl–N87 cell lines for our studies based on these data. We first assessed the effect of compounds **24a** and **25a** on cell viability in both cell lines at 24, 48, and 72 h using the MTT assay (Table 2). We found that the compounds exhibited EC_50_ values in the low micromolar range and that the cytotoxicity did not change significantly during this time (Table 2).

### 6.1. Effect of FPR1 blockade on cell growth

**Fig. 6.**
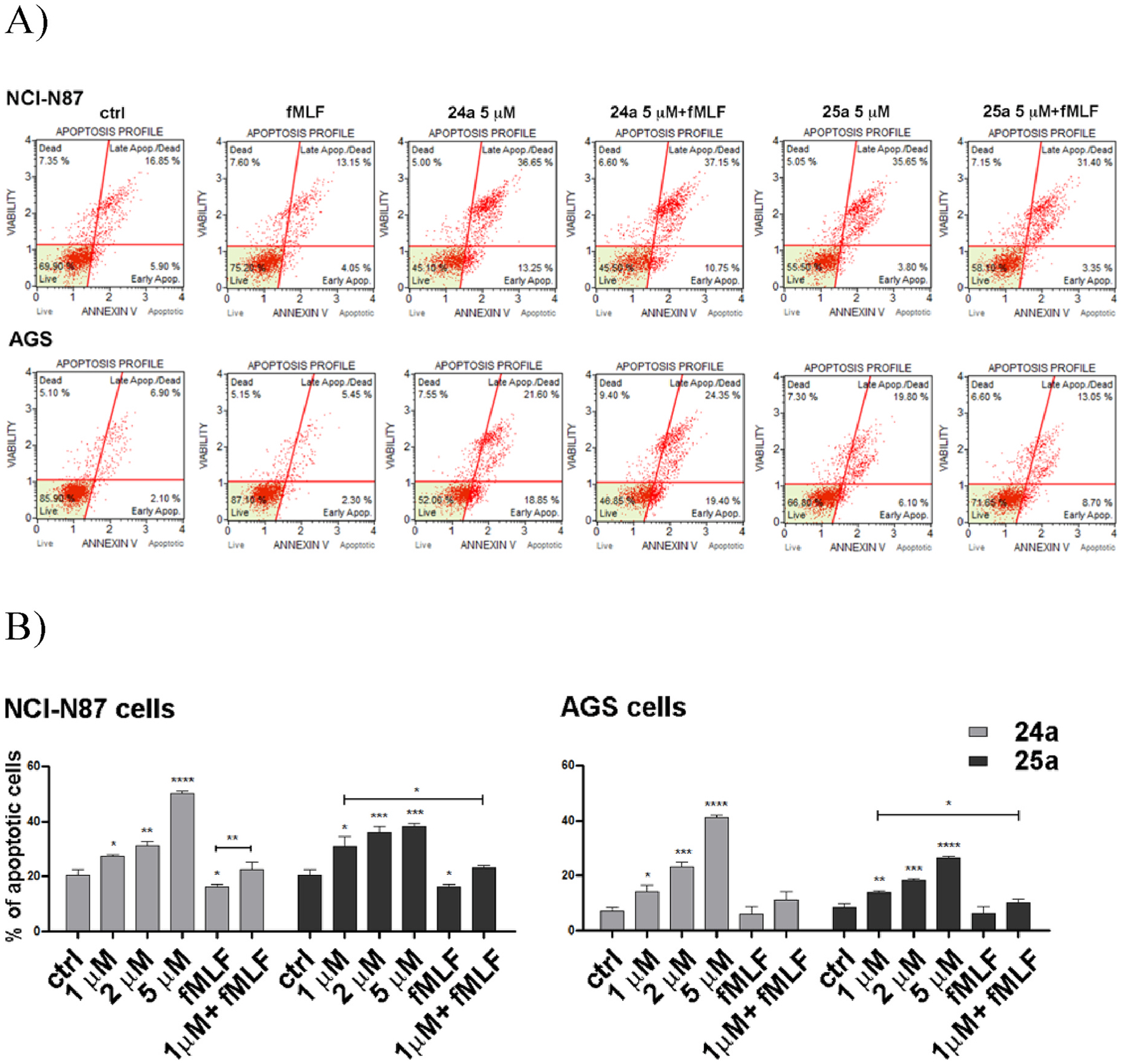
Apoptotic profile of **24a**, **25a** in NCl–N87 and AGS cell lines. Muse Annexin V Cell Assay was assessed after 48 h of drug treatment with *f*MLF (10 nM) and **24a** or **25a** (1, 2, 5 μM) administrated alone or combining the lowest dose of **24a** or **25a** with *f*MLF. Representative flow cytometry charts (panel A). Four cell populations can be distinguished relative to the percentage of cells alive (bottom left quadrant), in early apoptosis (bottom right quadrant), in late apoptosis (top right quadrant), and dead (top left quadrant). Statistical charts (panel B). The results derived from three independent experiments were expressed as means ± SD and reported in the relative graphs. Statistical analysis was assessed by comparing the values obtained using single drug treatment to those of corresponding untreated cells and the combined treatments to those of the single treatments, *p < 0.05; **p < 0.001; ***p < 0.0002; ****p < 0.0001.

Cell growth results from the balance between the rates of proliferation and of apoptosis, two events that exert opposite effects on cell growth. We evaluated the effects of the reference FPR1 agonist *f*MLF and the antagonists **24a** and **25a** on apoptosis and cell proliferation in AGS and NCl–N87 cells after 48 h of treatment. The compounds were tested at 1, 2, and 5 μM concentration based on the FPR1 antagonist potency and the results of cytotoxicity assays. To assess if the observed effects were FPR1-mediated, we co-administered the antagonists **24a** and **25a** with the FPR1 agonist *f*MLF (10 nM). In co-administration assays we chose a 10 nM dose of *f*MLF because higher doses induced appreciable cytotoxicity (data not shown). The apoptosis rate was assessed using the Annexin V cytofluorimetric assay. We found that *f*MLF (10 nM) induced a slight but significant reduction of apoptosis only in NCl–N87 cells, while **24a** and **25a** exhibited pro-apoptotic effects in both NCl–N87 and AGS cell lines. The effects were dose-dependent and, at lower doses, were blocked by *f*MLF (10 nM), thus suggesting that the observed effects were FPR1-mediated. Compound **24a** significantly increased the apoptosis rate, even at the lowest tested concentration (1 μM) as compared to the untreated cells in both cell lines. The effect was even more significant at the highest tested concentration (5 μM). Compound **25a** also exhibited a significant pro-apoptotic effect, albeit lower than that induced by **24a** (Fig. 6). In particular, compound **24a** induced a dose-dependent increase in the percentage of apoptosis in AGS cells, from 7.5% in untreated cells to 14% (1 μM), 23.3% (2 μM), and 42.2% at the highest dose (5 μM). Similarly, compound **25a** induced a dose-dependent increase of the apoptosis percentage in AGS cells, from 7.5% of untreated cells to 13.9% (1 μM), 18.2 (2 μM), and 26% at the highest dose (5 μM). Similar results were obtained in NCl–N87 cells. The percentage of apoptosis increased from 20.6% of untreated cells to 50.3% for the highest dose of **24a** and to 38.2% for the highest dose of **25a**. (Fig. 6). Interestingly, the growth-inhibiting effects were more pronounced in NCl–N87 cells, which expressed higher levels of FPR1.

**Fig. 7.**
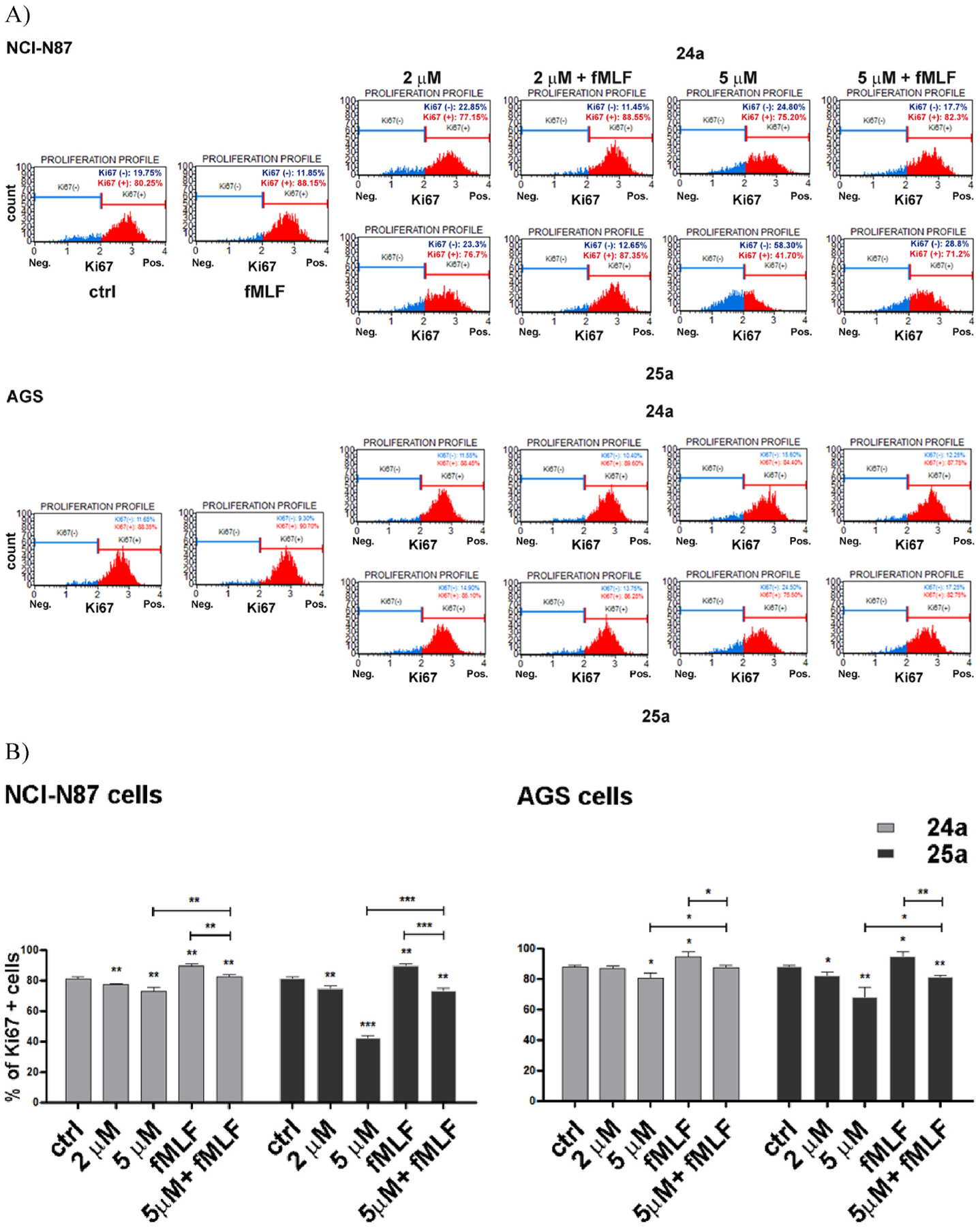
Effect of **24a** and **25a** on cell proliferation in NCl–N87 and AGS cell lines. Muse Ki67 Assay was assessed after 24 h of drug treatment with *f*MLF (10 nM) and **24a** or **25a** (2 or 5 μM) administrated alone or combining the higher dose of **24a** or **25a** with *f*MLF. Representative flow cytometry charts reporting percentage of Ki67 negative (blue) and positive (red) cells (panel A). Statistical charts reporting the results from three independent experiments and expressed as means ± SD (panel B). Statistical analysis was assessed by comparing the values obtained using single drug treatment to those of corresponding untreated cells and the combined treatments to those of the single treatments, *p < 0.05; **p < 0.001; ***p < 0.0001.

The effect of *f*MLF (10 nM), **24a**, and **25a** on proliferation rate was determined by evaluating the percentage of Ki67(+) cells after 24 h of drug treatment using the Muse Ki67 Proliferation Assay (Fig. 7). We found that *f*MLF induced a slight but significant induction of the cell proliferation rate. Compounds **24a** and **25a** dose-dependently inhibited cell proliferation in NCl–N87 and AGS cells with a more marked effect in the former cell line. In control NCl–N87 cells, the percentage of Ki67(+) cells was 81.3%, and the treatment with **24a** (5 μM) reduced the percentage to 73.1%, whereas **25a** (5 μM) reduced the percentage to 41.9%. In AGS cells, the treatment with **24a** induced negligible effects, while **25a** (5 μM) caused a significant effect (88% of Ki67(+) cells in control cells vs 68.2% in **25a**-treated cells). In both cell lines, the observed effects were FPR1-mediated, as they were partially reversed by coincubation with 10 nM *f*MLF.

### 6.2. Effect of FPR1 blockade on cell migration

**Fig. 8.**
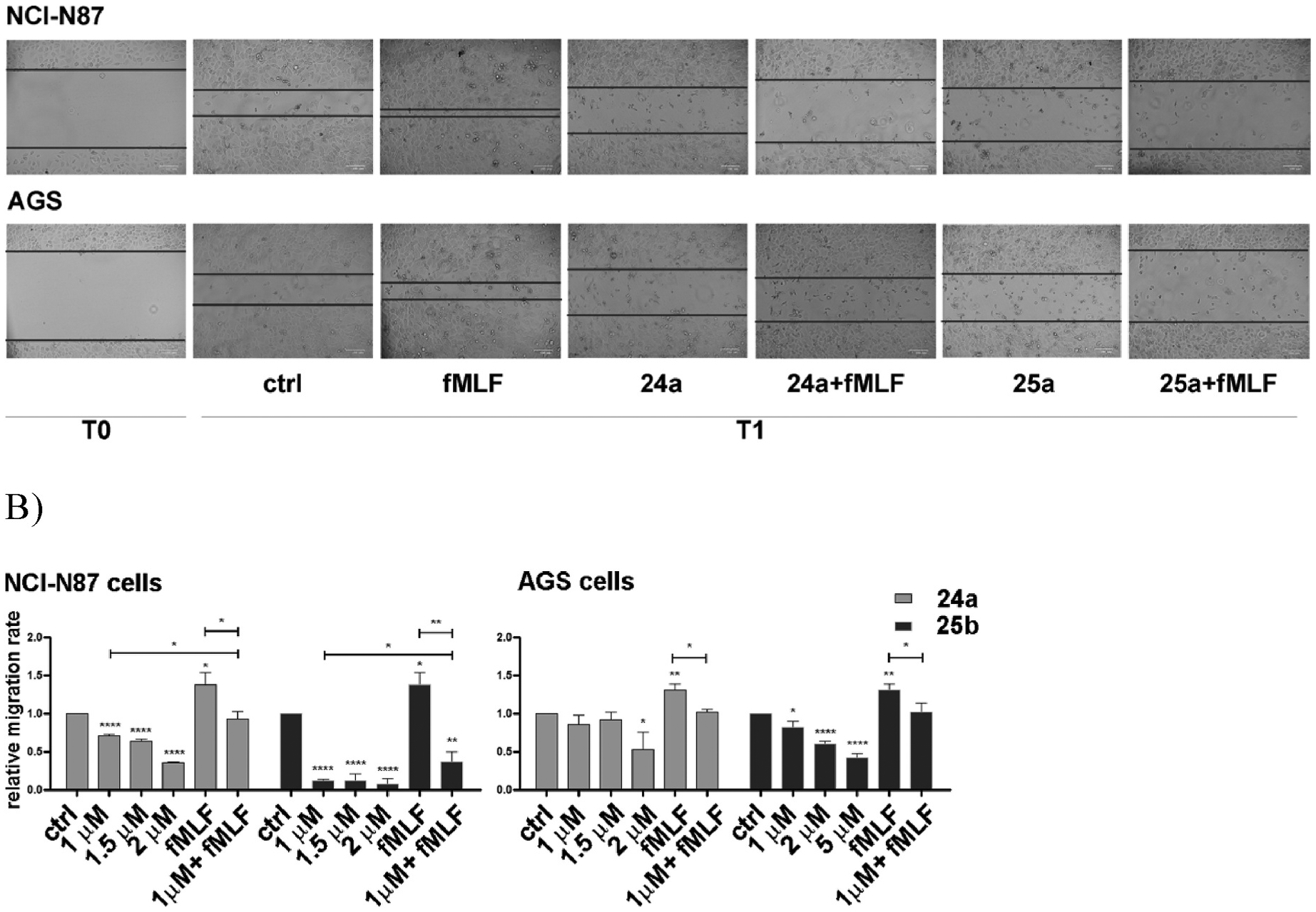
Effect of **24a** and **25a** on cell migration in NCl–N87 and AGS cell lines. Scratch assay assessed on cells treated with *f*MLF (10 nM) and **24a** or **25a** (1, 1.5, 2 μM) administrated alone or combining the lowest dose of **24a** or **25a** with *f*MLF. The cells were microscopically analyzed at the time of the scratch (T0) and after 24 h (T1). The relative migration rate was calculated by setting the percentage of migration of the control cells at time T1 equal to 1 and comparing the migration percentage of the cells after each drug treatment to this value. Representative original photographs (panel A) and quantitative analysis (panel B) of the cell-free scratch path ares. The experiments were performed in triplicate, and the mean values ± SD were plotted in the relative graph. Statistical analysis was assessed by comparing the values obtained using a single drug treatment to those of corresponding untreated cells and the combined treatments to those of the single treatments, *p < 0.05, **p < 0.001, ****p < 0.0001.

The effect of FPR1 blockade on cell migration was assessed using the scratch assay, an easy and well-developed method to measure cell migration in vitro. We found that 24 h incubation of NCl–N87 and AGS cells with *f*MLF (10 nM) significantly increased cell mobility (41% in NCl–N87 cells and 32% in AGS cells) (Fig. 8, Fig. S4). Conversely, **24a** and **25a** reduced the migration rate in a dose-dependent manner. The effect was more pronounced in NCl–N87 as compared to AGS cells. In NCl–N87 cells, **24a** (2 μM) induced a 64% reduction in the migration rate compared to untreated cells, whereas **25a** (2 μM) caused a 92% reduction in the migration rate. The effect was FPR1-mediated, as it was counterbalanced by co-administration of the agonist *f*MLF (10 nM).

### 6.3. Effect of FPR1 blockade on the expression and secretion of pro-angiogenesis factors

**Fig. 9.**
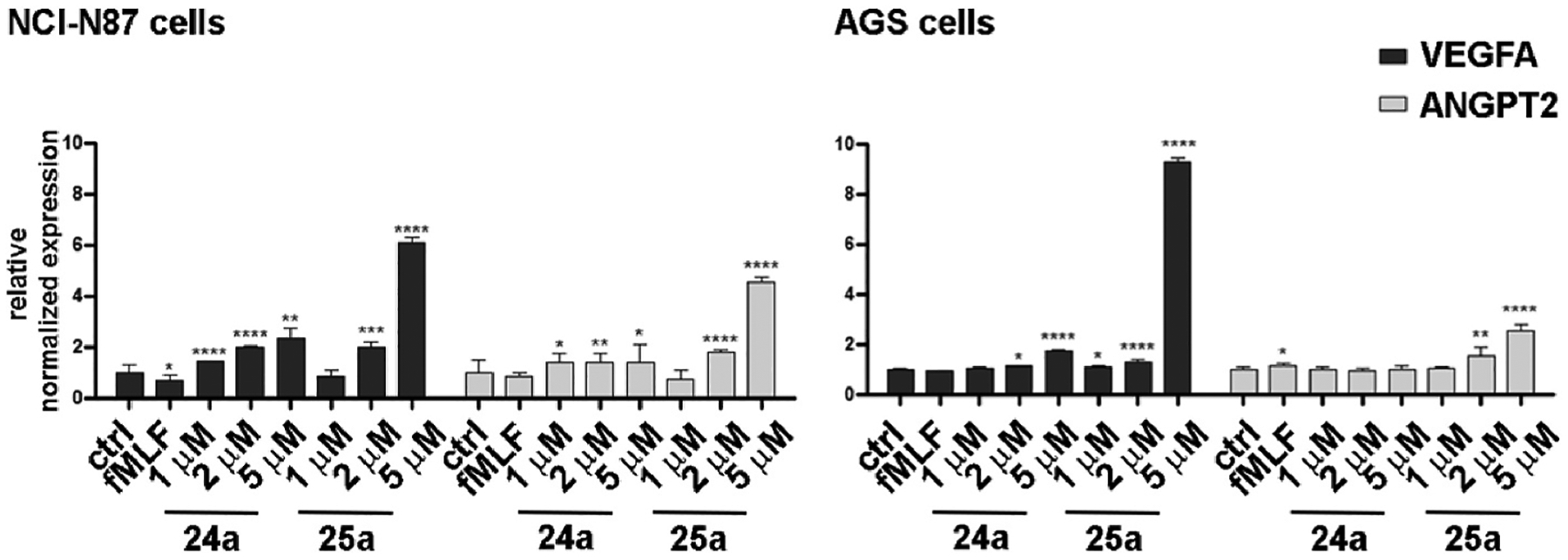
Effect of **24a** and **25a** on mRNA expression of VGFA and ANGPT2 in NCl–N87 and AGS cell lines. Real-time PCR experiments in NCl–N87 and AGS cells with specific primers for VEGFA and ANGPT2 genes. The mRNA expression was normalized on the GAPDH housekeeping gene. Expression analysis was performed after treatment with *f*MLF (10 nM) and **24a** or **25a** (1, 2, 5 μM) administrated alone or combining the lowest dose of **24a** or **25a** with *f*MLF. All expression values were calculated against the value of untreated PTX-sensitive cells, set equal to 1. Statistical analysis was assessed by comparing the values obtained using single drug treatment to those of corresponding untreated cells and the combined treatments to those of single treatments. The values of untreated PTX-resistant cells were compared with those of untreated PTX-sensitive ones. Data were mean ± SD (n = 3). *p < 0.05, **p < 0.001, ***p < 0.0002, ****p < 0.0001.

It has been reported that FPR1 can exert a tumor suppressor function in human GC by inhibiting angiogenesis and that silencing of the receptor increases the constitutive proangiogenic potential of GC cells. Therefore, we assessed the effect of **24a** and **25a** on the mRNA expression level and secretion of the proangiogenic factors, the vascular growth factor A (VEGFA), and the angiopoietin 2. We found that stimulation of FPR1 with the agonist *f*MLF (10 nM) reduced expression of both VGFA and angiopoietin 2 (ANGPT2) mRNA, whereas receptor blockade by **24a** and **25a** resulted in a dose-dependent increase in the mRNA expression for the two proangiogenic factors. These effects were more pronounced in NCl–N87 cells and resulted more strongly for **25a** than **24a**. The normalized relative expression of both genes after 24 h of treatment with 1 or 2 μM **24a** or **25b** is shown in Fig. 9. The values obtained after each treatment were normalized to the expression of GAPDH and compared to the reference value of the two genes in untreated cells, which were set equal to 1 (Fig. 9).

**Fig. 10.**
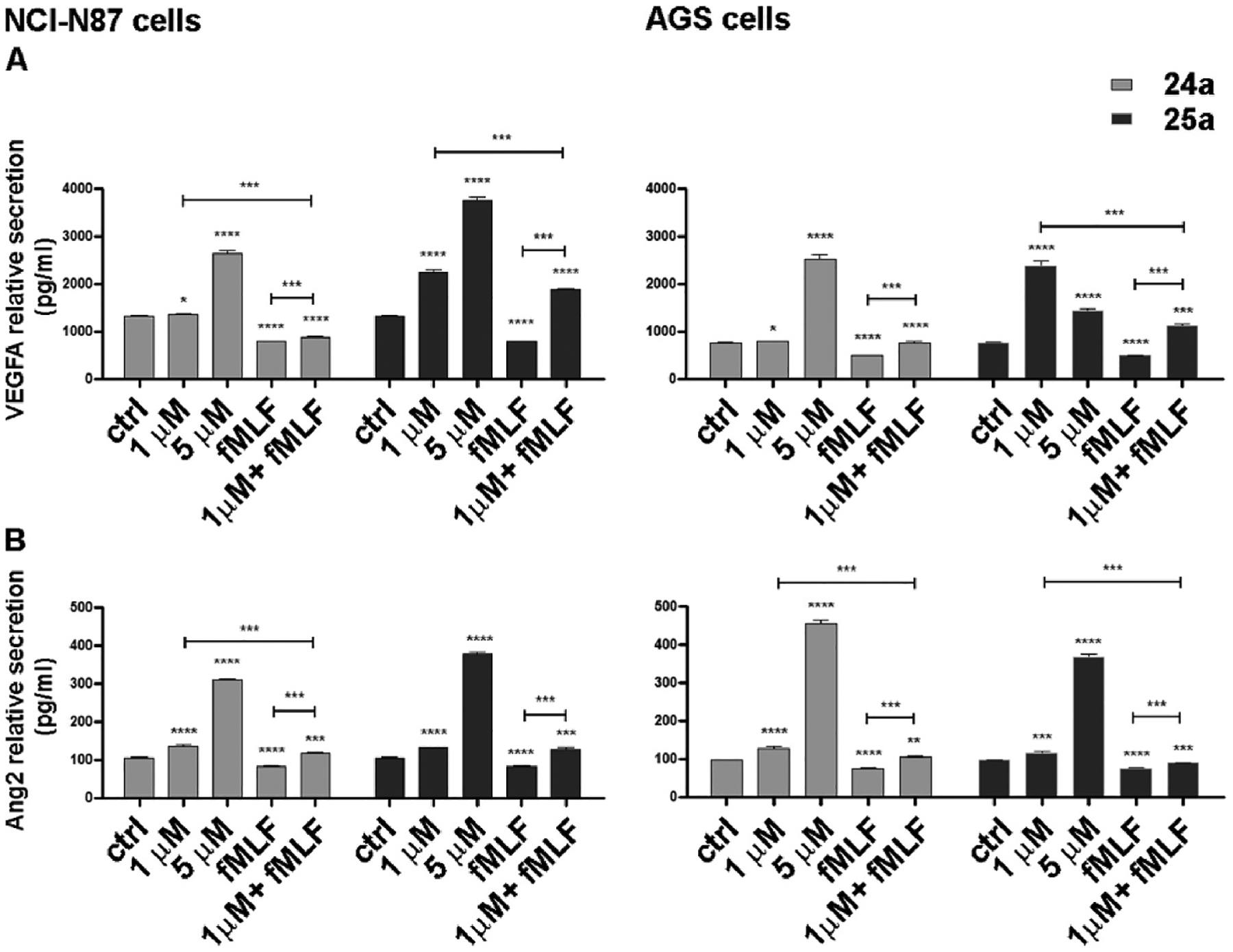
Effect of **24a** and **25a** on the secretion of VGFA (A) and Ang2 (B) in NCl–N87 and AGS cell lines. The ELISA assays were assessed on cells after 48 h of drug treatment with *f*MLF (10 nM) and **24a** or **25a** (1, 5 μM) administrated alone or combining the lowest dose of **24a** or **25a** with *f*MLF. The concentration of VEGFA (A) or Ang2 (B) was determined in the medium and normalized for the cell number. The values ± SD, obtained from three independent experiments expressed as pg/mL, were shown in the relative graphs. Statistical analysis was assessed by comparing the values obtained using a single drug treatment to those of corresponding untreated cells and the combined treatments to those of the single treatments. **p < 0.001; ***p < 0.0002; ****p < 0.0001.

Secretion of VEGFA and angiopoietin 2 (Ang2) in the culture medium of NCl–N87 and AGS cells was assessed after 48 h of drug treatment using ELISA methods (Fig. 10). Consistent with our mRNA data, we observed that stimulation of FPR1 with the agonist *f*MLF (10 nM) induced a reduction in the secretion of the two pro-angiogenic factors by approximately 40% (VEGFA) and 22% (Ang2) in NCl–N87 cells and 35% (VEGFA) and 23% (Ang2) in AGS. In contrast, treatment with **24a** and **25a** led to a dose-dependent increase in the secretion of both factors. Once again, the effects were more pronounced in NCl–N87 cells than in AGS, and the effect of **25a** was more pronounced as compared to **24a**. The co-administration of *f*MLF (10 nM) partially counteracted the effects.

Overall, the data obtained by blocking FPR1 activity through the antagonists **24a** and **25a** in AGS and NCl–N87 cells agree with those reported in the literature. While blocking FPR1 induces the inhibition of cellular processes important for tumor progression, it also results in the induction of factors such as VEGFA and angiopoietin 2, which are actively secreted by tumor cells into the tumor microenvironment where they not only constitute key growth factors for tumor development but also contribute to the formation of new vessels [8,17,30], thus suggesting that FPR1 could play a dual role as oncogene and onco-suppressor.

## 7. Conclusions

FPRs have been traditionally studied for their role in host-mediated immune responses, such as chemotaxis, phagocytosis, generation of reactive oxygen species, or cytokines release. Over the years, it has become evident that FPR1 also promote cancer progression by regulating the growth, survival, and invasiveness of cancer cells and, as such, they could be a valuable target in cancer treatment. This has been demonstrated in glioblastoma [31], neuroblastoma [9], and several cancer of digestive tract, such as hepatocellular, pancreatic, and colorectal carcinoma [32]. In GC, FPR1 activation provides a different scenario because it promotes cell growth and migration from one side and reduces angiogenesis from the other side [17,18]. In this study, we have identified potent and selective FPR1 antagonists and have studied their effects on cell growth and invasiveness in two cell models of GC, the third cause of cancer-related death. Continuing our work on FPR1 antagonists with 4*H*-chromen-4-one scaffold, we identified compounds **24a** and **25a**, which exhibited potent FPR1 antagonist activity. Molecular docking studies highlighted the key interactions formed by compounds **24a** and **25a** within the FPR1 binding cavity, identifying an additional lipophilic subpocket that is not occupied by the agonist *f*MIFL and which could be important for the antagonist activity of the compounds. The most potent antagonists emerging from this study, compounds **24a** and **25b**, were selected to study for the first time the effect of pharmacological blockade of FPR1 in AGS and NCl–N87 cells, two cell models of GC. Both compounds could potently inhibit cell growth through a combined effect on apoptosis and cell proliferation and reduce cell migration. On the other hand, compounds **24a** and **25a** increased the transcription and secretion of VEGFA and Angiopoietin 2, two angiogenic factors. Our data suggest that the pharmacological blockade of FPR1 exerts a potent inhibitory action on cell growth and motility; however, it also triggers resistance mechanisms by tumor cells which, through the secretion of pro-angiogenic factors, create a microenvironment that promotes cancer cells growth and, eventually, contribute to the onset of drug resistance. Because drug resistance is one of the major challenges for cancer research and clinical practice [33,34], the study of combined molecular therapies can be a winning weapon in cancer treatment. Our study might suggest that the synergic effect of FPR1 antagonists, such as compound **24a** and **25a**, and anti-angiogenic drugs could deserve further investigation in GC research. Finally, it is widely accepted that chronic inflammation plays a significant role in stomach tumorigenesis [38,39]. As a pattern recognition receptor, FPR1 is a key player in modulating immune response and inflammatory processes also in the gastric mucosa [6–8,17,18]. Thus, a deeper understanding of the effect of FPR1 blockade on the inflammatory mechanisms represents an important challenge for future research on gastric carcinogenesis.

## 8. Experimental section

### 8.1. Chemistry

Chemicals were purchased from Sigma-Aldrich, Alfa Aesar, TCI Chemicals. Unless otherwise stated, all chemicals were used without further purification. Thin layer chromatography (TLC) was performed using plates from Merck (silica gel 60 F254). Column chromatography was performed with 1:30 Merck silica gel 60 Å (63–200 μm) as the stationary phase. Flash chromatographic separations were performed on a Biotage SP1 purification system using flash cartridges pre-packed with KP-Sil 32–63 μm, 60 Å silica. ^1^H NMR spectra were recorded on a Varian Mercury-VX spectrometer (300 MHz) or on a 500-vnmrs500 Agilent spectrometer (500 MHz). All chemical shift values are reported in ppm (*δ*). Recording of mass spectra was performed on an HP6890-5973 MSD gas chromatograph/mass spectrometer; only significant *m*/*z* peaks, with their percentage of relative intensity in parentheses, are reported. High resolution mass spectra (electrospray ionization, ESI-TOF) (HRMS) were recorded on an Agilent 6530 Accurate Mass Q-TOF (mass range 50–3000 *m*/*z*, dry gas nitrogen 10 mL/min, dry heater 325 °C, capillary voltage 4000 V, electrospray ion source in positive or negative ion mode). All spectra were in accordance with the assigned structures. Elemental analyses (C,H,N) of the target compounds were performed on a Eurovector Euro EA 3000 analyzer. Analyses indicated by the symbols of the elements were within ±0.4% of the theoretical values. RP-HPLC analysis was performed on an Agilent 1260 Infinity Binary LC System equipped with a diode array detector using a Phenomenex Synergi Fusion-RP column (100 mm × 3 mm, 4 μm particle size). All target compounds were eluted with ACN/H_2_O, 7:3 at a flow rate of 0.8 mL/min. The purity of the target compounds listed in Table 1 was assessed by RP-HPLC and combustion analysis. All compounds exhibited ≥95% purity.

The following compounds were prepared according to the literature methods: 4-ethylbenzene-1,3-diol (**6**) [26]; 4-propylbenzene-1,3-diol (**7**) [27]; 4-butylbenzene-1,3-diol (**8**) [27]; 4-pentylbenzene-1,3-diol (**9**) [28].

#### 8.1.1. General procedure for the synthesis of ethanone derivatives 11a-c-12a-c, 13–15, 16a-c

To a cooled (0–5 °C) solution of the appropriate 4-alkylbenzene-1,3-diol (2.0 mmol) in anhydrous 1,2 dichlorobenzene (20 mL), anhydrous AlCl_3_ was added (2.0 mmol). The appropriate methoxyphenyl acetyl chloride (2.0 mmol) was slowly added, the reaction mixture was heated at 70 °C for 2 h under stirring, and the reaction mixture was cooled to room temperature and stirred overnight at this temperature. Subsequently, 3 N HCl was added (20 mL) and the reaction mixture was extracted with CH_2_Cl_2_ (2 × 30 mL). The collected organic layers were washed with brine, dried over anhydrous Na_2_SO_4_, and concentrated in vacuo. The crude residue was chromatographed as detailed below to obtain the pure desired compound.

##### 8.1.1.1. 1-(5-Hexyl-2,4-dihydroxyphenyl)-2-(2-methoxyphenyl)ethanone (11a).

Eluted with *n*-hexane/EtOAc 8:2. Yellow-orange oil, 53% yield. ^1^H NMR (300 MHz, CDCl_3_): *δ* 0.87–0.91 (m, 3H), 1.23–1.31 (m, 6H), 1.51–1.58 (m, 2H), 2.52 (t, 2H, *J* = 7.6 Hz), 3.82 (s, 3H), 4.23 (s, 2H), 5.77 (br s, 1H, D_2_O exchanged), 6.30 (s, 1H), 6.88–6.95 (m, 2H), 7.18–7.29 (m, 2H), 7.66 (s, 1H), 12.51 (1H, s, D_2_O exchanged). HRMS (ESI^–^) calcd for [(C_21_H_26_O_4_)–H]^−^: 341.1753, found 341.1759. ESI^–^/MS/MS [M – H]^−^ *m/z* 311 (100).

##### 8.1.1.2. 1-(5-Hexyl-2,4-dihydroxyphenyl)-2-(3-methoxyphenyl)ethanone (11b).

Gradient elution from *n*-hexane/EtOAc 7:3 to *n*-hexane/EtOAc 4:6. Yellow solid, 17% yield. ^1^H NMR (300 MHz, CDCl_3_) *δ* 0.89 (t, 3H *J* = 6.4 Hz), 1.25–1.31 (m, 6H), 1.53–1.55 (m, 2H), 2.52 (t, 2H, *J* = 7.6 Hz), 3.79 (s, 3H), 4.18 (s, 2H), 5.77 (br s, 1H, D_2_O exchanged), 6.30 (s, 1H), 6.79–6.87 (m, 2H), 7.22–7.27 (m, 2H), 7.57 (s, 1H), 12.48 (s, 1H, D_2_O exchanged). HRMS (ESI^–^) calcd for [(C_21_H_26_O_4_)–H]^−^: 341.1753, found 341. 1748. ESI^–^/MS/MS [M - H]^−^ *m/z* 219 (100), 175 (73). HRMS (ESI^–^) calcd for [(C_21_H_26_O_4)_–H]^–^: 341.1753, found 341.1746. ESI^–^/MS/MS [M – H]^−^ *m/z* 219 (100).

##### 8.1.1.3. 1-(5-Hexyl-2,4-dihydroxyphenyl)-2-(4-methoxyphenyl)ethanone (11c).

Gradient elution from CH_2_Cl_2_ to CH_2_Cl_2_/EtOAc 9:1. Yellow solid, 62% yield. ^1^H NMR (300 MHz, CDCl_3_) *δ* 0.87–0.91 (m, 3H), 1.30–1.32 (m, 6H), 1.51–1.58 (m, 2H), 2.53 (app t, 2H), 3.79 (s, 3H), 4.16 (s, 2H), 5.77 (br s, 1H, D_2_O exchanged), 6.30 (s, 1H), 6.88 (d, 2H, *J* = 8.8 Hz), 7.18 (d, 2H, *J* = 8.8 Hz), 7.57 (s, 1H), 12.52 (s, 1H, D_2_O exchanged). HRMS (ESI^–^) calcd for [(C_21_H_26_O_4_)–H]^−^: 341.1753, found 341. 1751. ESI^–^/MS/MS [M – H]^−^ *m/z* 220 (87), 163 (100).

##### 8.1.1.4. 2-(2-Methoxyphenyl)-1-(5-propyl-2,4-dihydroxyphenyl)ethanone (12a).

Eluted with *n*-hexane/EtOAc 8:2. Yellow oil, 10% yield. ^1^H NMR (500 MHz, CDCl_3_) *δ* 0.93–0.96 (m, 3H), 1.58–1.64 (m, 2H), 2.51 (app t, 2H), 3.81 (s, 3H), 4.22 (s, 2H), 5.77 (br s, 1H, D_2_O exchanged), 6.30 (s, 1H), 6.88–6.95 (m, 2H), 7.20 (dd, 1H, *J* = 1.5 Hz and *J* = 8.8 Hz), 7.24–7.28 (m, 1H), 7.66 (s, 1H), 12.51 (s, 1H, D_2_O exchanged). HRMS (ESI^–^) calcd for [(C_18_H_20_O_4_)–H]^−^: 299.1283, found 299.1284. ESI^–^/MS/MS [M – H]^−^ *m/z* 269 (100), 133 (64).

##### 8.1.1.5. 2-(3-Methoxyphenyl)-1-(5-propyl-2,4-dihydroxyphenyl)ethanone (12b).

Gradient elution from *n*-hexane/EtOAc 9:1 to *n*-hexane/EtOAc 8:2. Yellow solid, 24% yield. ^1^H NMR (300 MHz, CDCl_3_) *δ* 0.91–0.97 (m, 3H), 1.55–1.65 (m, 2H), 2.50 (t, 2H, *J* = 7.6 Hz), 3.79 (s, 3H), 4.18 (s, 2H), 5.77 (br s, 1H, D_2_O exchanged), 6.30 (s, 1H), 6.79–6.87 (m, 2H), 7.22–7.29 (m, 2H), 7.56 (s, 1H), 12.50 (s, 1H, D_2_O exchanged). HRMS (ESI^–^) calcd for [(C_21_H_26_O_4_)–H]^−^: 299.1283, found 299.1288. ESI^–^/MS/MS [M – H]^−^ *m/z* 269 (100).

##### 8.1.1.6. 2-(4-Methoxyphenyl)-1-(5-propyl-2,4-dihydroxyphenyl)ethanone (12c).

Gradient elution from *n*-hexane/EtOAc 8:2 to *n*-hexane/EtOAc 6:4. Yellow-orange solid, 10% yield. ^1^H NMR (300 MHz, CDCl_3_) *δ* 0.91–0.97 (m, 3H), 1.55–1.65 (m, 2H), 2.50 (t, 2H, *J* = 7.6 Hz), 3.79 (s, 3H), 4.18 (s, 2H), 5.77 (br s, 1H, D_2_O exchanged), 6.30 (s, 1H), 6.88 (d, 2H, *J* = 8.8 Hz), 7.18 (d, 2H, *J* = 8.8 Hz), 7.57 (s, 1H), 12.50 (s, 1H, D_2_O exchanged). HRMS (ESI^–^) calcd for [(C_18_H_20_O_4_)–H]^−^: 299.1283, found 299.1275. ESI^–^/MS/MS [M – H]^−^ *m/z* 178 (100), 133 (81), 121 (84).

##### 8.1.1.7. 1-(5-Ethyl-2,4-dihydroxyphenyl)-2-(2-methoxyphenyl)ethanone (13).

Eluted with *n*-hexane/EtOAc 8:2. Yellow-orange oil, 57% yield. ^1^H NMR (500 MHz, CDCl_3_) *δ* 1.21 (t, 3H, *J* = 7.3 Hz), 2.57 (q, 2H, *J* = 7.3 Hz), 3.82 (s, 3H), 4.24 (s, 2H), 5.77 (br s, 1H, D_2_O exchanged), 6.30 (s, 1H), 6.89–6.95 (m, 2H), 7.20 (dd, 1H, *J* = 1.5 Hz and *J* = 7.3 Hz), 7.26–7.28 (m, 1H), 7.68 (s, 1H), 12.53 (s, 1H, D_2_O exchanged). HRMS (ESI^–^) calcd for [(C_17_ H_18_O_4_)–H]^−^: 285.1127, found 285.1131. ESI^–^/MS/MS [M – H]^−^ *m/z* 119 (100).

##### 8.1.1.8. 1-(5-Butyl-2,4-dihydroxyphenyl)-2-(2-methoxyphenyl)ethanone (14).

Eluted with *n*-hexane/EtOAc 8:2. Yellowish oil. 16% Yield. ^1^H NMR (300 MHz, CDCl_3_) *δ* 0.94 (t, 3H, *J* = 7.3 Hz), 1.30–1.42 (m, 2H), 1.51–1.61 (m, 2H), 2.53 (app t, 2H), 3.82 (s, 3H), 4.23 (s, 2H), 5.77 (br s, 1H, D_2_O exchanged), 6.30 (s, 1H), 6.89–6.96 (m, 2H), 7.18–7.21 (m, 1H), 7.24–7.29 (m, 1H), 7.66 (s, 1H), 12.49 (s, 1H, D_2_O exchanged). HRMS (ESI^–^) calcd for [(C_19_H_22_ O_4_)–H]^−^: 313.1518, found 313.1513. ESI^–^/MS/MS [M – H]^−^ *m/z* 205 (100)

##### 8.1.1.9. 2-(2-Methoxyphenyl)-1-(5-pentyl-2,4-dihydroxyphenyl)ethanone (15).

Eluted with *n*-hexane/EtOAc 8:2. Yellow solid. 54% Yield. ^1^H NMR (300 MHz, CDCl_3_) *δ* 0.90 (t, 3H, *J* = 7.0 Hz), 1.30–1.37 (m, 4H), 1.54–1.62 (m, 2H), 2.52 (t, 2H, *J* = 7.6 Hz), 3.82 (s, 3H), 4.23 (s, 2H), 5.77 (br s, 1H, D_2_O exchanged), 6.30 (s, 1H), 6.89–6.96 (m, 2H), 7.18–7.27 (m, 2H), 7.66 (s, 1H), 12.50 (s, 1H, D_2_O exchanged). HRMS (ESI^–^) calcd for [(C_20_H_24_O_4_)–H]^−^: 327.1596, found 327.1589. ESI^–^/MS/MS [M – H]^−^ *m/z* 205 (100), 161 (81).

##### 8.1.1.10. 2-(2-Methoxyphenyl)-1-(5-methyl-2,4-dihydroxyphenyl)ethanone (16a).

Eluted with *n*-hexane/EtOAc 8:2. Yellow solid, 10% yield. ^1^H NMR (300 MHz, CDCl_3_) *δ* 2.49 (s, 3H), 3.82 (s, 3H), 4.23 (s, 2H), 5.77 (br s, 1H, D_2_O exchanged), 6.30 (s, 1H), 6.89–6.96 (m, 2H), 7.18–7.27 (m, 2H), 7.66 (s, 1H), 12.50 (s, 1H, D_2_O exchanged). HRMS (ESI^–^) calcd for [(C_16_H_16_ O_4_)–H]^−^: 271.0970, found 271.0968. ESI^–^/MS/MS [M – H]^−^ *m/z* 105 (100).

##### 8.1.1.11. 2-(3-Methoxyphenyl)-1-(5-methyl-2,4-dihydroxyphenyl)ethanone (16b).

Eluted with *n*-hexane/EtOAc 8:2. Yellow solid, 15% yield. ^1^H NMR (300 MHz, CDCl_3_) *δ* 2.52 (s, 3H), 3.82 (s, 3H), 4.22 (s, 2H), 5.77 (br s, 1H, D_2_O exchanged), 6.30 (s, 1H), 6.90–6.96 (m, 2H), 7.18–7.27 (m, 2H), 7.65 (s, 1H), 12.50 (s, 1H, D_2_O exchanged). HRMS (ESI^–^) calcd for [(C_16_H_16_O_4_)–H]^−^: 271.0970, found 271.0968. ESI^–^/MS/MS [M – H]^−^ *m/z* 105 (100).

##### 8.1.1.12. 2-(4-Methoxyphenyl)-1-(5-methyl-2,4-dihydroxyphenyl)ethanone (16c).

Eluted with *n*-hexane/EtOAc 8:2. Yellow solid, 10% yield. ^1^H NMR (300 MHz, CDCl_3_) *δ* 2.50 (s, 3H), 3.79 (s, 3H), 4.15 (s, 2H), 5.77 (br s, 1H, D_2_O exchanged), 6.31 (s, 1H), 6.86–6.91 (m, 2H), 7.17–7.20 (m, 2H), 7.29 (s, 1H), 12.54 (s, 1H, D_2_O exchanged). HRMS (ESI^–^) calcd for [(C_16_H_16_O_4_)–H]^−^: 271.0970, found 271.0969. ESI^–^/MS/MS [M – H]^−^ *m/z* 150 (100), 105 (81).

#### 8.1.2. General procedure for the synthesis of 4H-chromen-4-one derivatives 17a-c, 19a-c, 20–22, 23a-23c

To a mixture of the appropriate ethanone derivative **11a-c-12a-c, 13–15, 16a-c** (1.0 mmol) and K_2_CO_3_ (5.54 mmol) in anhydrous DMF (5 mL) acetic anhydride was added (0.39 mL, 4.06 mmol). The reaction mixture was stirred at 115 °C for 120 min, and the mixture was cooled and poured onto ice water. The solid was filtered, washed with water, and dried under reduced pressure. The crude solid was then chromatographed as detailed below to obtain the desired pure compound.

##### 8.1.2.1. 6-Hexyl-7-hydroxy-3-(2-methoxyphenyl)-2-methyl-4H-chromen-4-one (17a).

Gradient elution from *n*-hexane/EtOAc 8:2 to *n*-hexane/EtOAc 6:4. Yellow oil, 38% yield. ^1^H NMR (300 MHz, CDCl_3_) *δ* 0.84–0.89 (m, 3H), 1.23–1.30 (m, 6H), 1.55–1.63 (m, 2H), 2.18 (s, 3H), 2.62 (t, 2H, *J* = 7.6 Hz), 3.75 (s, 3H), 6.72 (s, 1H), 6.90–7.01 (m, 2H), 7.17 (dd, 1H, *J* = 1.8 Hz and *J* = 7.6 Hz), 7.30 (td, 1H, *J* = 1.8 Hz and *J* = 7.6 Hz), 7.84 (br s, 1H, D_2_O exchanged), 7.93 (s, 1H). HRMS (ESI^–^) calcd for [(C_23_H_26_O_4_)–H]^−^: 365.1753, found 365.1748. ESI^–^/MS/MS [M – H]^−^ *m/z* 365 (96), 349 (100).

##### 8.1.2.2. 6-Hexyl-7-hydroxy-3-(3-methoxyphenyl)-2-methyl-4H-chromen-4-one (17b).

Eluted with *n*-hexane/EtOAc 6:4. Brownish solid, 21% yield. ^1^H NMR (300 MHz, CDCl_3_) *δ* 0.84–0.89 (m, 3H), 1.23–1.30 (m, 6H), 1.55–1.63 (m, 2H), 2.22 (s, 3H), 2.52 (t, 2H, *J* = 7.6 Hz), 3.79 (s, 3H), 6.70 (s, 1H), 6.90–7.01 (m, 2H), 7.22–7.27 (m, 2H), 7.84 (br s, 1H, D_2_O exchanged), 7.93 (s, 1H). HRMS (ESI^–^) calcd for [(C_23_H_26_O_4_)–H]^−^:365.1753, found 365.1751. ESI^–^/MS/MS [M – H]^−^ *m/z* 365 (74), 350 (100).

##### 8.1.2.3. 6-Hexyl-7-hydroxy-3-(4-methoxyphenyl)-2-methyl-4H-chromen-4-one (17c).

Eluted with *n*-hexane/EtOAc 1:1. Brownish solid, 85% yield. ^1^H NMR (300 MHz, CDCl_3_) *δ* 0.87 (t, 3H, *J* = 7.0 Hz), 1.24–1.31 (m, 8H), 2.27 (s, 3H), 2.66 (t, 2H, *J* = 7.6 Hz), 3.83 (s, 3H), 6.77 (s, 1H), 6.90–6.96 (m, 2H), 7.18–7.21 (m, 2H), 7.84 (br s, 1H, D_2_O exchanged), 7.94 (s, 1H). HRMS (ESI^–^) calcd for [(C_23_H_26_O_4_)–H]^−^: 365.1753, found 365.1750. ESI^–^/MS/MS [M – H]^−^ *m/z* 350 (100).

##### 8.1.2.4. 7-Hydroxy-3-(2-methoxyphenyl)-2-methyl-6-propyl-4H-chromen-4-one (19a).

Eluted with *n*-hexane/EtOAc 8:2. Yellow oil, 47%, yield. ^1^H NMR (300 MHz, CDCl_3_) *δ* 0.93 (t, 3H, *J* = 7.4 Hz), 1.56–1.68 (m, 2H), 2.19 (s, 3H), 2.59 (app t, 2H), 3.75 (s, 3H), 6.73 (s, 1H), 6.87–6.92 (m, 1H), 6.96–7.01 (m, 1H), 7.19 (dd, 1H *J* = 1.7 Hz and 7.5 Hz), 7.26–7.32 (m, 21H), 7.93 (s, 1H). HRMS (ESI^–^) calcd for [(C_20_H_20_O_4_)–H]^−^: 323.1283, found 323.1278. ESI^–^/MS/MS [M – H]^−^ *m/z* 307 (100).

##### 8.1.2.5. 7-Hydroxy-3-(3-methoxyphenyl)-2-methyl-6-propyl-4H-chromen-4-one (19b).

Eluted with *n*-hexane/EtOAc 7:3. Yellow oil, 53% yield. ^1^H NMR (300 MHz, CDCl_3_) *δ* 0.91–0.97 (m, 3H), 1.55–1.65 (m, 2H), 2.26 (s, 3H), 2.55 (t, 2H, *J* = 7.6 Hz), 3.80 (s, 3H), 6.77 (s, 1H), 6.90–6.93 (m, 2H), 7.19–7.22 (m, 2H), 7.84 (br s, 1H, D_2_O exchanged), 7.94 (s, 1H). HRMS (ESI^–^) calcd for [(C_20_H_20_O_4_)–H]^−^: 323.1283, found 323.1290.ESI^–^/MS/MS [M – H]^−^ *m/z* 308 (100), 307 (92).

##### 8.1.2.6. 7-Hydroxy-3-(4-methoxyphenyl)-2-methyl-6-propyl-4H-chromen-4-one (19c).

Eluted with *n*-hexane/EtOAc 8:2. Yellow solid, 44% yield. ^1^H NMR (300 MHz, CDCl_3_) *δ* 0.91–0.97 (m, 3H), 1.55–1.65 (m, 2H), 2.27 (s, 3H), 2.56 (t, 2H, *J* = 7.6 Hz), 3.81 (s, 3H), 6.70 (s, 1H), 6.98 (d, 2H, *J* = 8.8 Hz), 7.22 (d, 2H, *J* = 8.8 Hz)), 7.82 (br s, 1H, D_2_O exchanged), 7.95 (s, 1H). HRMS (ESI^–^) calcd for [(C_20_H_20_O_4_)–H]^−^: 323.1283, found 323.1251. ESI^–^/MS/MS [M – H]^−^ *m/z* 308 (100).

##### 8.1.2.7. 6-Ethyl-7-hydroxy-3-(2-methoxyphenyl)-2-methyl-4H-chromen-4-one (20).

Gradient elution from *n*-hexane/EtOAc 8:2 to *n*-hexane/EtOAc 6:4. Yellow solid, 72% yield. ^1^H NMR (300 MHz, CDCl_3_) *δ* 1.21 (t, 3H, *J* = 7.3 Hz), 2.23 (s, 3H), 2.57 (q, 2H, *J* = 7.3 Hz), 3.75 (s, 3H), 6.71 (s, 1H), 6.90–6.93 (m, 2H), 7.19–7.22 (m, 2H), 7.82 (br s, 1H, D_2_O exchanged), 7.94 (s, 1H). HRMS (ESI^–^) calcd for [(C_19_H_18_O_4_)–H]^−^: 309.1127, found 309.1132. ESI^–^/MS/MS [M – H]^−^ *m/z* 293 (100).

##### 8.1.2.8. 6-Butyl-7-hydroxy-3-(2-methoxyphenyl)-2-methyl-4H-chromen-4-one (21).

Gradient elution from *n*-hexane/EtOAc 8:2 to *n*-hexane/EtOAc 6:4. Yellow solid, 52% yield. ^1^H NMR (300 MHz, CDCl_3_) *δ* 0.91 (t, 3H, *J* = 7.3 Hz), 1.31–1.41 (m, 2H), 1.54–1.64 (m, 2H), 2.19 (s, 3H), 2.63 (app t, 2H), 3.74 (s, 3H), 6.78 (s, 1H), 6.90–7.01 (m, 2H), 7.16 (dd, 1H, *J* = 1.7 Hz, *J* = 7.4 Hz), 7.27–7.33 (m, 1H), 7.93 (s, 1H), 8.01 (br s, 1H, D_2_O exchanged). HRMS (ESI^–^) calcd for [(C_21_H_22_O_4_)–H]-:21 4) H] : 337.1518, found 337.1565. ESI^–^/MS/MS [M – H]^−^ *m/z* 293 (100).

##### 8.1.2.9. 7-Hydroxy-3-(2-methoxyphenyl)-2-methyl-6-pentyl-4H-chromen-4-one (22).

Gradient elution from *n*-hexane/EtOAc 8:2 to *n*-hexane/EtOAc 6:4. Yellow solid. 48% Yield. ^1^H NMR (300 MHz, CDCl_3_) *δ* 0.85–0.89 (m, 3H), 1.30–1.44 (m, 4H), 1.56–1.66 (m, 2H), 2.18 (s, 3H), 2.63 (app t, 2H), 3.75 (s, 3H), 6.73 (s, 1H), 6.91–7.01 (m, 2H), 7.17 (dd, 1H, *J* = 1.8 Hz and *J* = 7.6 Hz), 7.27–7.33 (m, 1H), 7.70 (br s, 1H, D_2_O exchanged), 7.93 (s, 1H). HRMS (ESI^–^) calcd for [(C_22_H_24_O_4_)–H]^−^: 351.1596, found 351.1569. ESI^–^/MS/MS [M – H]^−^ *m/z* 335 (100).

##### 8.1.2.10. 2,6-Dimethyl-7-hydroxy-3-(2-methoxyphenyl)-4H-chromen-4-one (23a).

Gradient elution from *n*-hexane/EtOAc 8:2 to *n*-hexane/EtOAc 1:1. Yellow solid, 41% yield. ^1^H NMR (300 MHz, CDCl_3_) *δ* 2.18 (s, 3H), 2.23 (s, 3H), 3.75 (s, 3H), 6.73 (s, 1H), 6.91–7.01 (m, 2H), 7.17 (dd, *J* = 1.8 Hz and *J* = 7.6 Hz), 7.27–7.33 (m, 1H), 7.70 (br s, 1H, D_2_O exchanged), 7.93 (s, 1H). HRMS (ESI^–^) calcd for [(C_23_H_26_O_4_)–H]^−^: 295.0970, found 295.0969. ESI^–^/MS/MS [M – H]^−^ *m/z* 279 (100).

##### 8.1.2.11. 2,6-Dimethyl-7-hydroxy-3-(3-methoxyphenyl)-4H-chromen-4-one (23b).

Gradient elution from *n*-hexane/EtOAc 8:2 to *n*-hexane/EtOAc 1:1. Yellow solid, 41% yield. ^1^H NMR (300 MHz, CDCl_3_) *δ* 2.15 (s, 3H), 2.18 (s, 3H), 3.71 (s, 3H), 6.73 (s, 1H), 6.80–7.01 (m, 3H), 7.19–7.22 (m, 1H), 7.72 (br s, 1H, D_2_O exchanged), 7.78 (s, 1H). HRMS (ESI^–^) calcd for [(C_18_H_16_O_4_)–H]^−^: 295.0970, found 295.0965. ESI^–^/MS/ MS [M – H]^−^ *m/z* 279 (100).

##### 8.1.2.12. 2,6-Dimethyl-7-hydroxy-3-(4-methoxyphenyl)-4H-chromen-4-one (23c).

Gradient elution from *n*-hexane/EtOAc 8:2 to *n*-hexane/EtOAc 1:1. Yellow solid, 41% yield. ^1^H NMR (300 MHz, CDCl_3_) *δ* 2.15 (s, 3H), 2.18 (s, 3H), 3.71 (s, 3H), 6.73 (s, 1H), 6.82–6.98 (m, 2H), 7.18–7.21 (m, 2H), 7.70 (br s, 1H, D_2_O exchanged), 7.78 (s, 1H). HRMS (ESI^–^) calcd for [(C_18_H_16_O_4_)–H]^−^: 295.0970, found 295.0965. ESI^–^/MS/MS [M – H]^−^ *m/z* 279 (100).

#### 8.1.3. General procedure for the synthesis of 4H-chromen-4-one derivatives 18a-c

To a solution of the appropriate ethanone derivative **11a-c** (0.4 mmol) in anhydrous pyridine (5 mL) trifluoroacetic anhydride (1.0 mmol) was added. The reaction mixture was stirred at 40–50 °C for 15 min and then at room temperature overnight, the reaction mixture was diluted with 3 N HCl, and the aqueous solution was extracted with CH_2_Cl_2_ (2 × 30 mL). The collected organic layers were washed with brine, dried over Na_2_SO_4_, and concentrated in vacuo. The crude product was chromatographed as detailed below to obtain the desired pure compound.

##### 8.1.3.1. 6-Hexyl-7-hydroxy-3-(2-methoxyphenyl)-2-trifluoromethyl-4H-chromen-4-one (18a).

Eluted with *n*-hexane/EtOAc 8:2. Yellow solid, 33% yield. ^1^H NMR (300 MHz, CDCl_3_) *δ* 0.86–0.91 (m, 3H), 1.23–1.38 (m, 6H), 1.58–1.63 (m, 2H), 2.63 (app t, 2H), 3.75 (s, 3H), 4.85 (br s, 1H, D_2_O exchanged), 6.95–7.04 (m, 2H), 7.12 (dd, 1H, *J* = 1.8 Hz and *J* = 7.0 Hz), 7.34 (s, 1H), 7.38–7.44 (m, 1H), 8.08 (s, 1H). HRMS (ESI^–^) calcd for [(C_23_H_23_F_3_O_4_)–H]^−^: 419.1470, found 419.1454. ESI^–^/MS/MS [M – H]^−^ *m/z* 419 (80), 335 (100)

##### 8.1.3.2. 6-Hexyl-7-hydroxy-3-(3-methoxyphenyl)-2-trifluoromethyl-4H-chromen-4-one (18b).

Eluted with *n*-hexane/EtOAc 8:2. White solid, 47% yield. ^1^H NMR (500 MHz, CDCl_3_) *δ* 0.87–0.90 (m, 3H), 1.28–1.35 (m, 6H), 1.57–1.61 (m, 2H), 2.63 (t, 2H, *J* = 7.8 Hz), 3.82 (s, 3H), 5.05 (br s, 1H, D_2_O exchanged), 6.79–6.84 (m, 2H), 6.98 (dd, 1H, *J* = 2.0 Hz and *J* = 8.3 Hz), 7.36 (app t, 2H), 8.08 (s, 1H). HRMS (ESI^–^) calcd for [(C_23_H_23_F_3_O_4_)–H]^−^: 419.1470, found 419.1467. ESI^–^/MS/MS [M – H]^−^ *m/z* 419 (94), 404 (100).

##### 8.1.3.3. 6-Hexyl-7-hydroxy-3-(4-methoxyphenyl)-2-trifluoromethyl-4H-chromen-4-one (18c).

Eluted with *n*-hexane/EtOAc 7:3. Yellow solid, 10%. ^1^H NMR (500 MHz, CDCl_3_) *δ* 0.86–0.88 (m, 3H), 1.08–1.38 (m, 6H), 1.40–1.71 (m, 2H), 2.53 (app t, 2H), 3.80 (s, 3H), 5.04 (br s, 1H, D_2_O exchanged), 6.89 (d, 2H, *J* = 8.8 Hz), 7.14 (s, 1H), 7.28 (d, 2H, *J* = 8.8 Hz), 8.02 (s, 1H). HRMS (ESI^–^) calcd for [(C_23_H_23_F_3_O_4_)–H]^−^: 419.1470, found 419.1464. ESI^–^/MS/MS [M−H]^−^ *m/z* 419 (81), 404 (100).

#### 8.1.4. General procedure for the synthesis of compounds 31a-c and 32

To a solution of the appropriate phenol **7** and **10** (0.96 mmol), DMAP (0.096 mmol) and pyridine (0.33 mL) in anhydrous CH_2_Cl_2_ (10 mL), cooled at 0 °C, trifluoromethanesulfonic anhydride (0.32 mL, 1.9 mmol) was added dropwise, and the reaction mixture was stirred at room temperature for 4 h. After completion of the reaction, 1 M HCl (9 mL) was added, and the mixture was further stirred at room temperature for 10 min. The organic layer was separated, and the aqueous phase was extracted twice with CH_2_Cl_2_ (2×20 mL). The collected organic layers were washed with brine, dried over anhydrous Na_2_SO_4_, and concentrated in vacuo. The obtained crude trifluoromethanesulfonate was then mixed with palladium acetate (0.065 mmol), cesium carbonate (1.63 mmol), and BINAP (0.065 mmol) in anhydrous THF (20 mL), and benzophenone imine (0.84 mmol) was added. The reaction mixture was stirred at 80 °C for 22 h and then at room temperature for additional 24 h. After completion of the reaction, the reaction mixture was diluted with EtOAc (20 mL) and washed with H_2_O and brine. The collected organic layers were washed with brine, dried over anhydrous Na_2_SO_4_, and concentrated in vacuo. The crude residue was chromatographed using a mixture of *n-*hexane/EtOAc 9:1 as eluent to obtain the pure desired compound.

##### 8.1.4.1. 7-[(Diphenylmethylene)amino]-6-hexyl-3-(2-methoxyphenyl)-2-methyl-4H-chromen-4-one (31a).

Yellow solid. 22% yield. ^1^H NMR (500 MHz, CDCl_3_) *δ* 0.84 (app t, 3H), 1.24–1.29 (m, 6H), 1.65–1.71 (m, 2H), 2.12 (s, 3H), 2.68 (t, 2H, *J* = 7.8 Hz), 3.77 (s, 3H), 6.44 (s, 1H), 6.96–7.02 (m, 2H), 7.12–7.16 (m, 3H), 7.32–7.36 (m, 4H), 7.45–7.51 (m, 3H), 7.80 (broad s, 2H), 7.92 (s, 1H). HRMS (ESI^+^) calcd for [(C_36_H_35_NO_3_)+ Na]^+^: 552.2515, found 552.2522. ESI^+^/MS/MS [M+Na]^+^ *m/z* 552 (100).

##### 8.1.4.2. 7-[(Diphenylmethylene)amino]-6-hexyl-3-(3-methoxyphenyl)-2-methyl-4H-chromen-4-one (31b).

Red solid. 20% yield. ^1^H NMR (500 MHz, CDCl_3_) *δ* 0.84 (app t, 3H), 1.24–1.29 (m, 6H), 1.65–1.71 (m, 2H), 2.12 (s, 3H), 2.68 (t, 2H, *J* = 7.8 Hz), 3.77 (s, 3H), 6.45 (s, 1H), 6.96–7.02 (m, 2H), 7.10–7.12 (m, 3H), 7.32–7.35 (m, 4H), 7.45–7.51 (m, 3H), 7.80 (broad s, 2H), 7.93 (s, 1H). HRMS (ESI^+^) calcd for [(C_36_H_35_NO_3_)+Na]^+^: 552.2515, found 552.2543. ESI^+^/MS/MS [M+Na]^+^ *m/z* 552 (100).

##### 8.1.4.3. 7-[(Diphenylmethylene)amino]-6-hexyl-3-(4-methoxyphenyl)-2-methyl-4H-chromen-4-one (31c).

Yellow solid, 25% yield. ^1^H NMR (500 MHz, CDCl_3_) *δ* 0.84 (app t, 3H), 1.24–1.29 (m, 6H), 1.65–1.71 (m, 2H), 2.12 (s, 3H), 2.68 (t, 2H, *J* = 7.8 Hz), 3.77 (s, 3H), 6.41 (s, 1H), 6.96–7.02 (m, 2H), 7.15–7.19 (m, 3H), 7.32–7.35 (m, 4H), 7.45–7.51 (m, 3H), 7.80 (broad s, 2H), 7.93 (s, 1H). HRMS (ESI^+^) calcd for [(C_36_H_35_NO)+Na]^+^: 552.2515, found 552.2507. ESI^+^/MS/MS [M+Na]^+^ *m/z* 552 (100).

##### 8.1.4.4. 7-[(Diphenylmethylene)amino]-3-(2-methoxyphenyl)-2-methyl-6-propyl-4H-chromen-4-one (32).

Yellow solid, 15% yield. ^1^H NMR (500 MHz, CDCl_3_) *δ* 0.91–0.97 (m, 3H), 1.55–1.65 (m, 2H), 2.12 (s, 3H), 2.50 (app t, 2H), 3.77 (s, 3H), 6.45 (s, 1H), 6.96–7.02 (m, 2H), 7.10–7.12 (m, 3H), 7.32–7.35 (m, 4H), 7.45–7.51 (m, 3H), 7.80 (broad s, 2H), 7.93 (s, 1H). HRMS (ESI^+^) calcd for [(C_33_H_29_NO_3_)+Na]^+^ : 510.2045, found 510.2046. ESI^+^/MS/MS [M+Na]^+^ *m/z* 510 (100).

#### 8.1.5. General procedure for the synthesis of compounds 33a-c and 34

To the appropriate diphenylethylenamino derivate **31a-c** and **32** (0.15 mmol) in THF (5 mL), 2 M HCl (2 mL) was added. The mixture was stirred at room temperature for 1 h. After completion, the reaction mixture was alkalinized using diluted NH_4_OH. The reaction mixture was then extracted with EtOAc (3×20 mL). The collected organic layers were dried over anhydrous Na_2_SO_4_, filtered and concentrated in vacuo. The crude residue was chromatographed using a gradient of elution from *n-*hexane/EtOAc 8:2 to *n-*hexane/EtOAc 1:1 to obtain the pure target compound.

##### 8.1.5.1. 7-Amino-6-hexyl-3-(2-methoxyphenyl)-2-methyl-4H-chromen-4-one (33a).

Yellow solid, 90% yield. ^1^H NMR (300 MHz, CDCl_3_) *δ* 0.86–0.91 (m, 3H), 1.28–1.41 (m, 6H), 1.62–1.70 (m, 2H), 2.15 (s, 3H), 2.53 (app t, 2H), 3.76 (s, 3H), 4.15 (br s, 2H, D_2_O exchanged), 6.56 (s, 1H), 6.94–7.02 (m, 2H), 7.14–7.17 (m, 1H), 7.31–7.37 (m, 1H), 7.86 (s, 1H). HRMS (ESI^+^) calcd for [(C_23_H_27_NO_3_)+Na]^+^: 388.1889, found 388.1873. ESI^+^/MS/MS [M+Na]^+^ *m/z* 77 (100).

##### 8.1.5.2. 7-Amino-6-hexyl-3-(3-methoxyphenyl)-2-methyl-4H-chromen-4-one (33b).

Red oil, quantitative yield. ^1^H NMR (500 MHz, CDCl_3_) *δ* 0.87–0.90 (m, 3H), 1.32–1.38 (m, 4H), 1.39–1.41 (m, 2H), 1.62–1.69 (m, 2H), 2.24 (s, 3H), 2.54 (t, 2H, *J* = 7.8 Hz), 3.81 (s, 3H), 4.15 (br s, 2H, D_2_O exchanged), 6.58 (s, 1H), 6.83–6.90 (m, 3H), 7.30–7.33 (m, 1H), 7.88 (s, 1H). HRMS (ESI^+^) calcd for [(C_23_H_27_NO_3_)+Na]^+^: 388.1889, found 388.1886. ESI^+^/MS/MS [M+Na]^+^ *m/z* 388 (100).

##### 8.1.5.3. 7-Amino-6-hexyl-3-(4-methoxyphenyl)-2-methyl-4H-chromen-4-one (33c).

Brownish solid, 86% yield. ^1^H NMR (500 MHz, CDCl_3_) *δ* 0.87–0.90 (m, 3H), 1.30–1.33 (m, 4H), 1.38–1.41 (m, 2H), 1.62–1.68 (m, 2H), 2.25 (s, 3H), 2.54 (t, 2H, *J* = 7.8 Hz, 2H), 3.83 (s, 3H), 4.15 (br s, 2H, D_2_O exchanged), 6.58 (s, 1H), 6.95 (d, 2H, *J* = 8.3 Hz), 7.20 (d, 2H, *J* = 8.8 Hz), 7.87 (s, 1H). HRMS (ESI^+^) calcd for [(C_23_H_27_NO3)+Na]^+^: 388.1889, found 388.1890. ESI^+^/MS/MS [M+Na]^+^ *m/z* 388 (100).

##### 8.1.5.4. 7-Amino-3-(2-methoxyphenyl)-2-methyl-6-propyl-4H-chromen-4-one (34).

Yellow solid, 70% yield. ^1^H NMR (500 MHz, CDCl_3_) *δ* 1.00 (t, 3H, *J* = 7.3 Hz), 1.66–1.71 (m, 2H), 2.16 (s, 3H), 2.51 (t, 2H, *J* = 7.8 Hz), 3.76 (s, 3H), 4.15 (br s, 2H, D_2_O exchanged), 6.56 (s, 1H), 6.95 (d, 1H, *J* = 7.8 Hz), 6.99–7.02 (m, 1H), 7.16 (d, 1H, *J* = 7.3 Hz), 7.32–7.35 (m, 1H), 7.86 (s, 1H). HRMS (ESI^+^) calcd for [(C_20_H_21_NO_3_)+Na]^+^: 346.1419, found 346.1416. ESI^+^/MS/MS [M+Na]^+^ *m/z* 346 (100).

#### 8.1.6. General procedure for the synthesis of final compounds 24a-c-26a-c, and 27–29

A mixture of the appropriate phenol **17a-c-19a-c** and **20–22** (0.11 mmol), pyridine (0.24 mL) and acetic anhydride (0.4 mL, 0.46 mmol) was stirred at room temperature for 4 days. The reaction mixture was poured into 3 N HCl (10 mL) and the reaction mixture was extracted with CH_2_Cl_2_ (2 × 20 mL). The collected organic layers were washed with brine, dried over Na_2_SO_4_, and concentrated under reduced pressure. The crude residue was chromatographed as detailed below to obtain the desired target compound.

##### 8.1.6.1. 6-Hexyl-3-(2-methoxyphenyl)-2-methyl-4-oxo-4H-chromen-7-yl acetate (24a).

Gradient elution from *n*-hexane/EtOAc 8:2 to *n*-hexane/EtOAc 6:4. Pale yellow solid. 64% Yield. ^1^H NMR (500 MHz, CDCl_3_) *δ* 0.88 (t, 3H, *J* = 7.0 Hz), 1.28–1.35 (m, 6H), 1.57–1.63 (m, 2H), 2.20 (s, 3H), 2.37 (s, 3H), 2.60 (app t, 2H), 3.76 (s, 3H), 6.98 (d, 1H, *J* = 8.5 Hz), 7.01–7.04 (m, 1H), 7.16 (dd, 1H, *J* = 1.5 Hz and 7.5 Hz), 7.19 (s, 1H), 7.34–7.38 (m, 1H), 8.08 (s, 1H). HRMS (ESI^+^) calcd for [(C_25_H_28_O_5_)+Na]^+^: 431.1834, found 431.1831. ESI^+^/MS/MS [M+Na]^+^ *m/z* 109 (100).

##### 8.1.6.2. 6-Hexyl-3-(3-methoxyphenyl)-2-methyl-4-oxo-4H-chromen-7-yl acetate (24b).

Gradient elution from *n*-hexane/EtOAc 8:2 to *n*-hexane/EtOAc 6:4. White solid, 52% yield. ^1^H NMR (500 MHz, CDCl_3_) *δ* 0.88 (app t, 3H), 1.27–1.34 (m, 6H), 1.57–1.63 (m, 2H), 2.29 (s, 3H), 2.38 (s, 3H), 2.61 (t, 2H, *J* = 7.8 Hz), 3.82 (s, 3H), 6.81–6.84 (m, 2H), 6.90–6.92 (m, 1H), 7.20 (s, 1H), 7.34 (t, 1H, *J* = 7.8 Hz), 8.08 (s, 1H). HRMS (ESI^+^) calcd for [(C_25_H_28_O_5_)+Na]^+^: 431.1834, found 431.1832. ESI^+^/MS/MS [M+Na]^+^ *m/z* 431 (100).

##### 8.1.6.3. 6-Hexyl-3-(4-methoxyphenyl)-2-methyl-4-oxo-4H-chromen-7-yl acetate (24c).

Gradient elution from *n*-hexane/EtOAc 7:3 to *n*-hexane/EtOAc 1:1. White solid, 16% yield. ^1^H NMR (300 MHz, CDCl_3_) *δ* 0.85–0.90 (m, 3H), 1.24–1.32 (m, 6H), 1.57–1.61 (m, 2H), 2.29 (s, 3H), 2.97 (s, 3H), 2.60 (app t, 2H), 3.84 (s, 3H), 6.95–6.98 (m, 2H), 7.16–7.21 (s + m, 3H), 8.07 (s, 1H). HRMS (ESI^+^) calcd for [(C_25_H_28_O_5_)+Na]^+^: 431.1834, found 431.1834. ESI^+^/MS/MS [M+Na]^+^ *m/z* 389 (92), 431 (100).

##### 8.1.6.4. 6-Hexyl-3-(2-methoxyphenyl)-4-oxo-2-(trifluoromethyl)-4H-chromen-7-yl acetate (25a).

Gradient elution from *n*-hexane/EtOAc 9:1 to *n*-hexane/EtOAc 8:2. Yellow-orange oil, 56% yield. ^1^H NMR (300 MHz, CDCl_3_) *δ* 0.86–0.91 (m, 3H), 1.23–1.38 (m, 6H), 1.58–1.63 (m, 2H), 2.39 (s, 3H), 2.63 (app t, 2H), 3.75 (s, 3H), 6.95–7.04 (m, 2H), 7.12 (dd, 1H, *J* = 1.8 Hz and 7.0 Hz), 7.34 (s, 1H), 7.38–7.44 (m, 1H), 8.08 (s, 1H). HRMS (ESI^+^) calcd for [(C_25_H_25_F_3_O_5_)+Na]^+^: 485.1552, found 485.1548. ESI^+^/MS/MS [M+Na]^+^ *m/z* 104 (85), 485 (100).

##### 8.1.6.5. 6-Hexyl-3-(3-methoxyphenyl)-4-oxo-2-(trifluoromethyl)-4H-chromen-7-yl acetate (25b).

Eluted with *n*-hexane/EtOAc 8:2. White solid, 64% yield. ^1^H NMR (500 MHz, CDCl_3_) *δ* 0.87–0.90 (m, 3H), 1.28–1.35 (m, 6H), 1.57–1.61 (m, 2H), 2.39 (s, 3H), 2.63 (t, 2H, *J* = 7.8 Hz), 3.82 (s, 3H), 6.79–6.84 (m, 2H), 6.98 (dd, 1H, *J* = 2.0 Hz and 8.3 Hz), 7.36 (app t, 2H), 8.08 (s, 1H). HRMS (ESI^+^) calcd for [(C_25_H_25_F_3_O_5_)+Na]^+^: 485.1552, found 485.1828. ESI^+^/MS/MS [M+Na]^+^ *m/z* 77 (100).

##### 8.1.6.6. 6-Hexyl-3-(4-methoxyphenyl)-4-oxo-2-(trifluoromethyl)-4H-chromen-7-yl acetate (25c).

Eluted with *n*-hexane/EtOAc 8:2. White solid, 64% yield. ^1^H NMR (300 MHz, CDCl_3_) *δ* 0.88 (t, 3H, *J* = 6.7 Hz), 1.28–1.36 (m, 6H), 1.55–1.62 (m, 2H), 2.39 (s, 3H), 2.63 (t, 2H, *J* = 7.7 Hz), 3.85 (s, 3H), 6.98 (d, 2H, *J* = 8.7 Hz), 7.18 (d, 2H, *J* = 8.7 Hz), 7.34 (s, 1H), 8.04 (s, 1H). HRMS (ESI^+^) calcd for [(C_25_H_25_F_3_O_5_)+Na]^+^: 485.1552, found 485.1555. ESI^+^/MS/MS [M+Na]^+^ *m/z* 485 (100).

##### 8.1.6.7. 3-(2-Methoxyphenyl)-2-methyl-4-oxo-6-propyl-4H-chromen-7-yl acetate (26a).

Eluted with *n*-hexane/EtOAc 8:2. Yellow solid, 71% yield. ^1^H NMR (300 MHz, CDCl_3_) *δ* 0.96 (t, 3H, *J* = 7.3 Hz), 1.60–1.68 (m, 2H), 2.20 (s, 3H), 2.37 (s, 3H), 2.58 (app t, 2H), 3.76 (s, 3H), 6.96–7.05 (m, 2H), 7.15 (dd, 1H, *J* = 1.8 Hz and 7.5 Hz), 7.19 (s, 1H), 7.33–7.39 (m, 1H), 8.08 (s, 1H). HRMS (ESI^+^) calcd for [(C_22_H_22_O_5_)+Na]^+^: 389.1365, found 389.1361. ESI^+^/MS/MS [M+Na]^+^ *m/z* 76 (80), 347 (100).

##### 8.1.6.8. 3-(3-Methoxyphenyl)-2-methyl-4-oxo-6-propyl-4H-chromen-7-yl acetate (26b).

Eluted with *n*-hexane/EtOAc 8:2. Yellow solid, 14% yield. ^1^H NMR (300 MHz, CDCl_3_) *δ* 0.96 (t, 3H, *J* = 7.3 Hz), 1.60–1.68 (m, 2H), 2.18 (s, 3H), 2.37 (s, 3H), 2.58 (app t, 2H), 3.76 (s, 3H), 6.96–7.05 (m, 2H), 7.15 (dd, 1H, *J* = 1.8 Hz and 7.5 Hz), 7.19 (s, 1H), 7.33–7.39 (m, 1H), 8.06 (s, 1H). HRMS (ESI^+^) calcd for [(C_22_H_22_O_5_)+Na]^+^: 389.1365, found 389.1361. ESI^+^/MS/MS [M+Na]^+^ *m/z* 77 (100).

##### 8.1.6.9. 3-(4-Methoxyphenyl)-2-methyl-4-oxo-6-propyl-4H-chromen-7-yl acetate (26c).

Eluted with CH_2_Cl_2_/EtOAc 9:1. Yellow solid, 58% yield. ^1^H NMR (300 MHz, CDCl_3_) *δ* 0.95 (app t, 3H), 1.60–1.68 (m, 2H), 2.30 (s, 3H), 2.38 (s, 3H), 2.59 (app t, 2H), 3.85 (s, 3H), 6.97 (d, 2H, *J* = 8.8 Hz), 7.18–7.21 (m, 3H), 8.08 (s, 1H). HRMS (ESI^+^) calcd for [(C_22_H_22_O_5_)+Na]^+^: 389.1365, found 389.1368. ESI^+^/MS/MS [M+Na]^+^ *m/z* 77 (100), 347 (89).

##### 8.1.6.10. 6-Ethyl-3-(2-methoxyphenyl)-2-methyl-4-oxo-4H-chromen-7-yl acetate (27).

Eluted with *n*-hexane/EtOAc 7:3. Yellow oil, 60% yield. ^1^H NMR (300 MHz, CDCl_3_) *δ* 1.24 (t, 3H, *J* = 7.6 Hz), 2.20 (s, 3H), 2.38 (s, 3H), 2.64 (q, 2H, *J* = 7.6 Hz), 3.76 (s, 3H), 6.96–7.05 (m, 2H), 7.15 (dd, 1H, *J* = 1.7 and 7.0 Hz), 7.19 (s, 1H), 7.33–7.39 (m, 1H), 8.11 (s, 1H). HRMS (ESI^+^) calcd for [(C_21_H_20_O_5_)+Na]^+^: 375.1208, found 375.1206. ESI^+^/MS/MS [M+Na]^+^ *m/z* 104 (72), 333 (100).

##### 8.1.6.11. 6-Butyl-3-(2-methoxyphenyl)-2-methyl-4-oxo-4H-chromen-7-yl acetate (28).

Eluted with *n*-hexane/EtOAc 7:3. Yellow oil, 52% yield. ^1^H NMR (300 MHz, CDCl_3_) *δ* 0.93 (app t, 3H), 1.30–1.42 (m, 2H), 1.54–1.64 (m, 2H), 2.20 (s, 3H), 2.38 (s, 3H), 2.61 (app t, 2H), 3.76 (s, 3H), 6.96–7.04 (m, 2H), 7.14–7.20 (s+m, 2H), 7.33–7.38 (m, 1H), 8.09 (s, 1H). HRMS (ESI^+^) calcd for [(C_23_H_24_O_5_)+Na]^+^: 403.1516, found 403.1521. ESI^+^/MS/MS [M+Na]^+^ *m/z* 403 (100).

##### 8.1.6.12. 3-(2-Methoxyphenyl)-2-methyl-4-oxo-6-pentyl-4H-chromen-7-yl acetate (29).

Eluted with *n*-hexane/EtOAc 7:3. Yellow oil, 80% yield. ^1^H NMR (300 MHz, CDCl_3_) *δ* 0.89 (app t, 3H), 1.28–1.37 (m, 4H), 1.56–1.66 (m, 2H), 2.20 (s, 3H), 2.37 (s, 3H), 2.60 (app t, 2H), 3.76 (s, 3H), 6.96–7.05 (m, 2H), 7.16 (dd, 1H, *J* = 1.8 Hz and 7.5 Hz), 7.19 (s, 1H), 7.33–7.39 (m, 1H), 8.08 (s, 1H). HRMS (ESI^+^) calcd for [(C_24_H_26_O_5_)+Na]^+^: 417.1672, found 417.1670. ESI^+^/MS/MS [M+Na]^+^ *m/z* 417 (100).

##### 8.1.6.13. 7-(2,2-Difluoroethoxy)-6-hexyl-3-(2-methoxyphenyl)-2-methyl-4H-chromen-4-one (30).

A solution of phenol **17a** (0.18 g, 0.50 mmol), 2,2-difluoroethyl methanesulfonate (0.09 g, 0.55 mmol) and K_2_CO_3_ (0.14 g, 1 mmol) in anhydrous DMF (3 mL) was stirred at 90 °C for 16 h. After completion, the reaction mixture was diluted with H_2_O (10 mL) and extracted with AcOEt (3×20 mL). The organic layers were collected and washed with brine, dried over anhydrous Na_2_SO_4_, and concentrated in vacuo. The crude residue was chromatographed with *n-*hexane/AcOEt 8:2 to obtain a yellow oil (32% yield). ^1^H NMR (300 MHz, CDCl_3_) *δ* 0.88 (app t, 3H), 1.28–1.36 (m, 6H), 1.57–1.60 (m, 2H), 2.20 (s, 3H), 2.68 (app t, 2H), 3.77 (s, 3H), 4.26 (td, 2H, *J*_*H-H*_ = 4.1 Hz, *J*_*H-F*_ = 12.8 Hz), 6.17 (tt, 1H, *J*_*H-H*_ = 4.1 Hz, *J*_*H-F*_ = 55.0 Hz), 6.77 (s, 1H), 6.97–7.04 (m, 2H), 7.16 (dd, 1H, *J* = 1.8 Hz and 6.5 Hz), 7.33–7.39 (m, 1H), 7.97 (s, 1H). HRMS (ESI^+^) calcd for [(C_25_H_28_F_2_O_4_)+Na]^+^: 453.1848, found 453.1857. ESI^+^/MS/MS [M+Na] *m/z* 453 (100).

#### 8.1.7. General procedure for the synthesis of final compounds 35a-c and 36

To a cooled (10 °C) solution of the appropriate amino derivate **33a-c** and **34** (0.8 mmol) in CH_2_Cl_2_, acetyl chloride (0.05 mL, 0.69 mmol) and Et_3_N (0.12 mL, 0.85 mmol) were added. The mixture was stirred at the same temperature for 5 min and then at room temperature for 1–5 h. After completion of the reaction, H_2_O (10 mL) was added to the reaction mixture. The organic layer was separated, and the aqueous phase was extracted with CH_2_Cl_2_ (2×10 mL). The collected organic layers were dried over anhydrous Na_2_SO_4_, filtered and concentrated in vacuo. The crude residue was chromatographed as detailed below to obtain the pure desired compound.

##### 8.1.7.1. N-[6-Hexyl-3-(2-methoxyphenyl)-2-methyl-4-oxo-4H-chromen-7-yl]acetamide (35a).

Eluted with *n*-hexane/EtOAc 6:4. White solid, 70% yield. ^1^H NMR (500 MHz, CDCl_3_) *δ* 0.88–0.90 (m, 3H), 1.31–1.41 (m, 4H), 1.60–1.68 (m, 4H), 2.20 (s, 3H), 2.27 (s, 3H), 2.64 (t, 2H, *J* = 7.8 Hz), 3.48 (s, 3H), 6.97–7.03 (m, 2H), 7.16 (dd, 1H, *J* = 1.5 Hz, *J* = 7.3 Hz), 7.33–7.37 (m, 2H), 7.98 (s, 1H), 8.37 (broad s, 1H, D_2_O exchanged). HRMS (ESI^+^) calcd for [(C_25_H_29_NO_4_)+Na]+: 430.1994, found 430.2002. ESI^+^/MS/MS [M+Na]^+^ m/z 77 (93), 430 (100).

##### 8.1.7.2. N-[6-Hexyl-3-(3-methoxyphenyl)-2-methyl-4-oxo-4H-chromen-7-yl]acetamide (35b).

Eluted with CH_2_Cl_2_/EtOAc 19:1. Yellow solid, 53% yield. ^1^H NMR (300 MHz, CDCl_3_) *δ* 0.87–0.91 (m, 3H), 1.28–1.42 (m, 6H), 1.59–1.70 (m, 2H), 2.27 (s, 3H), 2.29 (s, 3H), 2.64 (t, 2H, *J* = 7.6 Hz), 3.82 (s, 3H), 6.82–6.92 (m, 2H), 7.31–7.36 (m, 2H), 7.98 (s, 1H), 8.38 (br s, 1H, D_2_O exchanged). HRMS (ESI^+^) calcd for [(C_25_H_29_NO_4_) +Na]^+^: 430.1994, found 430.1993. ESI^+^ MS/MS [M+Na]^+^ *m/z* 430 (100).

##### 8.1.7.3. N-[6-Hexyl-3-(4-methoxyphenyl)-2-methyl-4-oxo-4H-chromen-7-yl]acetamide (35c).

Eluted with CH_2_Cl_2_/EtOAc 19:1. White solid, 90% yield. ^1^H NMR (300 MHz, CDCl_3_) *δ* 0.87–0.91 (m, 3H), 1.24–1.41 (m, 6H), 1.59–1.70 (m, 2H), 2.27 (s, 3H), 2.30 (s, 3H), 2.64 (t, 2H, *J* = 7.6 Hz), 3.84 (s, 3H), 6.95 (d, 2H, *J* = 8.8 Hz), 7.19 (d, 2H, *J* = 8.8 Hz), 7.30 (s, 1H), 7.98 (s, 1H), 8.37 (br s, 1H, D_2_O exchanged). HRMS (ESI^+^) calcd for [(C_25_H_29_NO_4_)+Na]^+^: 430.1994, found 430430.1984. ESI^+^/MS/MS [M+Na]^+^ *m/z* 430 (100).

##### 8.1.7.4. N-[3-(2-methoxyphenyl)-2-methyl-4-oxo-6-propyl-4H-chromen-7-yl]acetamide (36).

Eluted with CH_2_Cl_2_/EtOAc, 9:1. Colorless oil, 71% yield. ^1^H NMR (500 MHz, CDCl_3_) *δ* 0.99 (t, *J* = 7.3 Hz, 3H), 1.64–1.71 (m, 2H), 2.20 (s, 3H), 2.25 (s, 3H), 2.62 (app t, 2H), 3.75 (s, 3H), 6.96 (d, 1H, *J* = 8.3 Hz), 7.01 (app t, 1H), 7.16 (dd, 1H, *J* = 1.5 Hz and 7.3 Hz), 7.33–7.40 (m, 2H), 7.97 (s, 1H), 8.35 (s, 1H, D_2_O exchanged). HRMS (ESI^+^) calcd for [(C_22_H_23_NO_4_)+Na]^+^: 388.1525, found 388.1534. ESI^+^/MS/MS [M+Na]^+^ m/z 388 (100).

#### 8.1.8. General procedure for the synthesis of final compounds 37a-c

To a cooled (0–5 °C) solution of the appropriate phenol **16a-c** (0.34 mmol), DMAP (0.034 mmol) and Et_3_N (0.1 mL) in anhydrous DMF (5 mL) was added dropwise under stirring to a DMF solution of heptanoyl chloride, prepared from the corresponding acid (0.05 g, 0.36 mmol) and SOCl_2_ (5 mL). The reaction mixture was stirred at room temperature for 2 h and was diluted with H_2_O (10 mL), and extracted with AcOEt (3× 20 mL). The collected organic layers were washed with brine, dried over anhydrous Na_2_SO_4_, and concentrated in vacuo. The crude residue was chromatographed as detailed below to obtain the pure desired compound.

##### 8.1.8.1. 3-(2-Methoxyphenyl)-2,6-dimethyl-4-oxo-4H-chromen-7-yl heptanoate (37a).

Eluted with *n-*hexane/AcOEt 8:2. Yield 30%. ^1^H NMR (300 MHz, CDCl_3_) *δ* 0.91 (app t, 3H), 1.33–1.44 (m, 6H), 1.74–1.84 (m, 2H), 2.19 (s, 3H), 2.26 (s, 3H), 2.63 (app t, 2H), 3.75 (s, 3H), 6.96–7.04 (m, 2H), 7.14–7.17 (m, 2H), 7.33–7.39 (m, 1H), 8.07 (s, 1H). HRMS (ESI^+^) calcd for [(C_25_H_28_O_5_)+Na]^+^: 431.1834, found 431.1831. ESI^+^/MS/MS [M+Na]^+^ m/z 319 (100).

##### 8.1.8.2. 3-(3-Methoxyphenyl)-2,6-dimethyl-4-oxo-4H-chromen-7-yl heptanoate (37b).

Eluted with *n-*hexane/AcOEt 8:2. Yield 35%. ^1^H NMR (300 MHz, CDCl_3_) *δ* 0.91 (app t, 3H), 1.33–1.44 (m, 6H), 1.74–1.84 (m, 2H), 2.19 (s, 3H), 2.26 (s, 3H), 2.63 (app t, 2H), 3.75 (s, 3H), 6.95–7.04 (m, 2H), 7.13–7.15 (m, 2H), 7.33–7.42 (m, 1H), 8.07 (s, 1H). HRMS (ESI^+^) calcd for [(C_25_H_28_O_5_)+Na]^+^: 431.1834, found 431.1829. ESI^+^/MS/MS [M+Na]^+^ *m/z* 319 (100).

##### 8.1.8.3. 3-(4-Methoxyphenyl)-2,6-dimethyl-4-oxo-4H-chromen-7-yl heptanoate (37c).

Eluted with *n-*hexane/AcOEt 8:2. Yield 37%. ^1^H NMR (300 MHz, CDCl_3_) *δ* 0.92 (app t, 3H), 1.32–1.44 (m, 6H), 1.74–1.84 (m, 2H), 2.27 (s, 3H), 2.30 (s, 3H), 2.63 (app t, 2H), 3.77 (s, 3H), 6.95–6.98 (m, 2H), 7.17–7.18 (m, 2H), 7.21 (s, 1H), 8.07 (s, 1H). HRMS (ESI^+^) calcd for [(C_25_H_28_O_5_)+Na]^+^: 431.1834, found 431.1849. ESI^+^/MS/MS [M+Na]^+^ *m/z* 319 (100).

#### 8.1.9. General procedure for the preparation of carbamates 38 and 39

A mixture of the phenol **17a** and **19a** (0.08 g, 0.22 mmol), *N,N*-dimethylcarbamoylcarbonate (0.03 mL, 0.33 mmol) and potassium carbonate (0.05 g, 0.33 mmol) in acetonitrile (5 mL) was refluxed for 5 h. After cooling, the reaction mixture was concentrated under reduced pressure. The crude residue was dissolved in H_2_O and extracted in CH_2_Cl_2_ (2× 20 mL). The organic layers were collected, washed first with 1 M NaOH and then with brine. Finally, the organic layers were dried over anhydrous Na_2_SO_4_ and concentrated. The crude residue was chromatographed as detailed below to obtain the pure desired compound.

##### 8.1.9.1. 6-Hexyl-3-(2-methoxyphenyl)-2-methyl-4-oxo-4H-chromen-7-yl carbamate (38).

Eluted with *n-*hexane/AcOEt 6:4. 42%. ^1^H NMR (300 MHz, CDCl_3_) *δ* 0.85–0.89 (m, 3H), 1.25–1.36 (m, 6H), 1.59–1.66 (m, 2H), 2.20 (s, 3H), 2.64 (t, 2H, *J* = 7.8 Hz), 3.05 (s, 3H), 3.10 (s, 3H), 3.75 (s, 3H), 6.96–7.04 (m, 2H), 7.14–7.17 (m, 1H), 7.26 (d, 1H, *J* = 0.9 Hz), 7.33–7.39 (m, 1H), 8.06 (s, 1H). HRMS (ESI^+^) calcd for [(C_26_H_31_NO_5_)+ Na]^+^: 460.2094, found 460.2103. ESI^+^/MS/MS [M+Na]^+^ *m/z* 460 (100).

##### 8.1.9.2. 3-(2-Methoxyphenyl)-2-methyl-4-oxo-6-propyl-4H-chromen-7-yl dimethylcarbamate (39).

Eluted with *n-*hexane/AcOEt 6:4. 35%. ^1^H NMR (300 MHz, CDCl_3_) *δ* 0.94 (app t, 3H), 1.57–1.69 (m, 2H), 2.18 (s, 3H), 2.60 (app t, 2H), 3.05 (s, 3H), 3.10 (s, 3H), 3.75 (s, 3H), 6.73 (s, 1H), 6.90–7.01 (m, 2H), 7.17 (dd, 1H, *J* = 1.8 Hz and *J* = 7.5 Hz), 7.28–7.33 (m, 1H), 7.93 (s, 1H). HRMS (ESI^+^) calcd for [(C_23_H_25_NO_5_)+Na]^+^: 418.1625, found 418.1635. ESI^+^/MS/MS [M+Na]^+^ *m/z* 418 (100).

### 8.2. Molecular modeling studies

Initial 3D structures of compounds **24a**, **25a**, **30**, **35a**, and **37a**, **38** were built with ChemOffice Professional (PerkinElmer, Waltham, MA) and refined by molecular mechanics with the MM2 force field [35]. For the docking computations, Molegro Virtual Docker (MVD) software, version 6.0 (CLC Bio, Copenhagen, Denmark) was used. The structure of FPR1 in complex with Gi and peptide agonist *f*Met-Ile-Phe-Leu (*f*MIFL) (7VFX entry of the Protein Data Bank) determined by cryo-EM method [29] was used for the docking studies. Chain R complexed with *f*MIFL was extracted from the 7VFX structure using the UCSF Chimera 1.16 program (University of California, USA); water molecules and the ligand were deleted. Hydrogen atoms were added to the chain R protein and their positions were optimized using Maestro software. Protonation states of the acidic and basic residues were adjusted to pH 7.0 according to PROPKA prediction. The prepared protein and the structures of the investigated chromone derivatives were imported in the MVD program. Cavities in the protein were found with the “Detect cavities” instrument incorporated in MVD. The largest cavity of 963 Å^3^ in volume corresponds to the location of the *f*MIFL peptide. A search for docking poses was performed within a spherical area of 11 Å radius positioned at the geometric center of the peptide. Five hundred docking runs with MolDock score were performed for each compound considering full flexibility of a ligand around all rotatable bonds with rigid receptor. “Internal HBond” and “sp^2^-sp^2^ Torsions” options were enabled on ligand evaluation. “Constrain poses to cavity” option was switched on. The post-docking optimizations of ligand conformation and H-bonds were applied. The docking poses were analyzed and visualized using built-in facilities of MVD software.

### 8.3. Biology

#### 8.3.1. Ca^2+^ mobilization assay

Changes in intracellular Ca^2+^ were measured with a FlexStation II scanning fluorometer (Molecular Devices, Sunnyvale, CA, USA) as previously reported [36]. The cells, suspended in Hank’s balanced salt solution without Ca^2+^ and Mg^2+^ but with 10 mM HEPES (HBSS^−^), were loaded with 1.25 μg/mL Fluo-4 AM dye and incubated for 30 min in the dark at 37 °C. After dye loading, the cells were washed with HBSS^−^containing 10 mM HEPES, resuspended in HBSS^+^ containing Ca^2+^, Mg^2+^, and 10 mM HEPES (HBSS^+^), and aliquoted into the wells of flat-bottom, half-area-well black microtiter plates (2 × 10^5^ cells/well). For the evaluation of direct agonist activity, compounds were added from a source plate containing dilutions of test compounds in HBSS^+^, and changes in fluorescence were monitored (λ_ex_ = 485 nm, λ_em_ = 538 nm) every 5 s for 240 s at room temperature after the automated addition of compounds. Maximum change in fluorescence during the first 3 min, expressed in arbitrary units over baseline, was used to determine a response. Responses to agonists were normalized to the responses induced by the positive control peptides: 5 nM *f*MLF for FPR1-HL60 cells or 5 nM WKYMVM for FPR2-HL60 cells. Responses induced by the positive control peptides were assigned a value of 100%. To evaluate inhibitory effects of the compounds on FPR1/FPR2-dependent Ca^2+^ flux, the compounds were added to the wells (final concentration of DMSO was 1%) with FPR1/FPR2 HL60 cells. The samples were pre-incubated for 10 min, followed by addition of 5 nM *f*MLF (for FPR1-HL60 cells) or 5 nM WKYMVM (for FPR2-HL60 cells). The maximum change in fluorescence, expressed in arbitrary units over baseline, was used to determine the agonist response. Curve fitting (at least five or six points) and calculation of median effective concentration values (IC_50_) were performed by nonlinear regression analysis of the dose–response curves generated using Prism 7 (GraphPad Software, Inc., San Diego, CA, USA). Efficacy was determined by comparing individual responses activated by the test compounds to that induced by a positive control (5 nM *f*MLF for FPR1-HL60 cells or 5 nM WKYMVM for FPR2-HL60 cells), which was assigned a value of 100%.

#### 8.3.2. Stability assay in rat liver microsomes

Microsomal stability assays were performed as previously reported [37]. Test compounds were pre-incubated at 37 °C with rat liver microsomes (Tebu-Bio, Milan, Italy) (1.0 mg/mL microsomal protein) at a 10 μM final concentration in 100 mM potassium phosphate buffer (pH 7.4) for 10 min. Metabolic reactions were initiated by the addition of the NADPH regenerating system (containing 10 mM NADP, 50 mM glucose-6-phosphate, and 10 unit/mL glucose-6-phosphate dehydrogenase, final glucose-6-phosphate dehydrogenase concentration, 1 unit/mL). After 30 min, the mixture was quenched by adding an equal volume of cold acetonitrile containing the internal standard. Test compounds incubated with microsomes without the NADPH regenerating system were included. Quenched samples were centrifuged at 4500 rpm for 15 min and the supernatants were injected for quantification analysis. Samples (100 μL) were analyzed by using an Agilent 1260 Infinity Binary LC System equipped with a diode array detector (Open Lab software was used to analyze the chromatographic data) and a Phenomenex Gemini C-8 column (250 × 4.6 mm, 5 μm particle size). The samples were eluted using CH_3_CN/H_2_O (70:30, v/v) as the eluent (1 mL/min). Concentrations were quantified by measuring the area under the peak. The percentage of the parent compound remaining after a 30-min incubation was calculated according to the equation:

$$\%\text{ of parent compound remaining after }30\text{ min}={\text{C}}_{\text{parent}}/{\text{C}}_{\text{control}}•100$$

where C_parent_ is test compound concentration after incubation with the microsome fraction and NADPH regenerating system and C_control_ is test compound concentration after incubation with the microsome fraction only.

#### 8.3.3. Viability assay

##### Materials.

AGS and NCl–N87 human gastric cancer cell lines were purchased and authenticated by American Type Culture Collection (Manassas, Virginia, USA). All cell culture components were purchased from Sigma-Aldrich (Milan, Italy) and Celbio s.r.l. (Milano, Italy).

##### Cell cultures.

AGS and NCl–N87 human gastric cancer cell lines were grown in RPMI high glucose supplemented with 10% fetal bovine serum, 2 mM glutamine, 100 U/mL penicillin, 100 μg/mL streptomycin, in a humidified incubator at 37 °C with a 5% CO_2_ atmosphere.

##### Viability assay.

Determination of cell growth was performed using the MTT assay at 24h, 48 h, and 72 h. On day 1, 5000 cells cells/well were seeded into 96-well plates in a volume of 100 μL. On day 2, the test compound was added at different concentrations. In all the experiments, the solvent used to dissolve the drug (ethanol, DMSO) was added in each control to evaluate possible cytotoxicity. After the established incubation time with drugs, MTT (0.5 mg/mL) was added to each well. Following 3 h incubation at 37 °C, the supernatant was removed. The formazan crystals were solubilized using 100 μL of DMSO and the absorbance values at 570 and 630 nm were determined on a Victor 3 microplate reader (PerkinElmer).

#### 8.3.4. Apoptosis assay

NCl–N87 and AGS cells were treated with 10 nM *f*MLF and compounds **24a** or **25a** at different concentrations 1–5 μM administrated alone or in combination for 48 h. After specified drug treatments, the cells were processed using the Muse Annexin V/Dead Cell Assay Kit (Luminex Corporation, Austin, USA) for quantitative analysis of live, early/late apoptotic, and dead cells on a Muse Cell Analyzer. Briefly, the assay utilized Annexin V to detect phosphatidyl serine on the external membrane of apoptotic cells. The fluorescent signal emitted by dyeconjugated antibodies was detected by flow cytometry technology (Muse Cell Analyzer, Luminex Corporation, Austin, USA). 7-Amino-Actinomycin D (7-AAD) dead cell marker was also used. The cells were then analyzed according to the user’s guide.

#### 8.3.5. Ki67 cell proliferation assay

NCl–N87 and AGS cells were treated with 10 nM *f*MLF and **24a** or **25a** at different concentrations (1–5 μM) administrated alone or in combination for 48 h. After specified drug treatments, the cells were processed by the Muse Ki67 cell proliferation kit, which identified actively proliferating cells based on the expression of Ki67, a nuclear protein present in the active phases of the cell cycle (G1, S, G2, and M phases) and absent in the resting G0 phase. The Muse Ki67 Proliferation Assay was used with the Guava Muse Cell Analyzer according to manufacturer’s instructions (Luminex Corporation, Austin, USA). Briefly, the cells were stained after fixation and permeabilization procedures by using Hu Ki67 or Hu IgG1 control fluorochrome-conjugated antibodies to distinguish Ki67^+^ or Ki67^−^ cells, respectively. The software provided the percentage of both Ki67(+) and Ki67(−) cells.

#### 8.3.6. Migration assay

NCl–N87 and AGS cells were grown until confluence. A scratch wound was generated with a pipette tip. After rinsing with medium to remove detached cells, a low serum medium (1% FBS) with different drug concentrations was added. Photographs were taken of each well immediately (T0) and after various times at T1 (9 h), T2 (24 h), and T3 (48 h), using a Leica DMRXA camera (Leica Microsystems, Milan, Italy). Images were analyzed using ImageJ Software (http://rsb.info.nih.gov/ij/). The distance that cells migrated through the area created by scratching was determined by measuring the wound width at T1, T2 and T3 and subtracting it from the wound width at the start (T0). The relative migration rate was calculated by setting the percentage of migration of the control cells at time T2 equal to 1 and comparing the percentage of migration of the cells after each drug treatment to this value. The results are representative of three independent experiments.

#### 8.3.7. Gene expression analysis

Total RNA was extracted from FPR1 antagonist-treated NCl–N87 and AGS treated cells using the Qiagen RNeasy Mini Kit (Qiagen, Hilden, Germany) following the manufacturer’s instructions. Samples were retro-transcribed using the iScript Advanced cDNA Synthesis Kit (Bio-Rad Laboratories, California, USA). Real-Time-PCRs analyzed cDNA samples for the evaluation of VEGFA and ANGPT2 expression. Experiments were carried out in triplicate using the SsoAdvanced Universal SYBR Green Supermix (Bio-Rad Laboratories, California, USA) on a CFX96 Touch Real-Time PCR Detection System (Bio-Rad Laboratories, California, USA) according to the manufacturer’s instructions. The mRNA expression was normalized on that of the GAPDH housekeeping gene. Pre-validated PrimePCR templates for SYBR Green Assay (Bio-Rad Laboratories, California, USA) was used for reactions. Relative quantification was performed using the ddCT method.

#### 8.3.8. Measurement of VEGFA and angiopoietin 2 in cell culture medium

The amount of VEGFA and VEGFC secreted in the culture medium by NCl–N87 and AGS treated cells was measured using a highly sensitive Enzyme-Linked Immunosorbent Assay (ELISA) Quantikine Kit ELISA (R&D Systems, Minneapolis, MN, USA) according to the manufacturer’s instructions. The measured values were normalized for the number of cells.

#### 8.3.9. Statistical analysis

GraphPad Prism 5.0 software (La Jolla, CA, USA) was used to evaluate the differences between two unmatched groups by Mann–Whitney nonparametric test. P < 0.05 was considered statistically significant. All experiments were performed in triplicate and repeated three times. Data were presented as mean ± standard deviation (SD).

## Supplementary Material

Supplementary Material

## Figures and Tables

**Table 1:** Antagonist effect of target compounds on Ca^2+^ mobilization in FPR1-and FPR2-HL60 transfected cells.

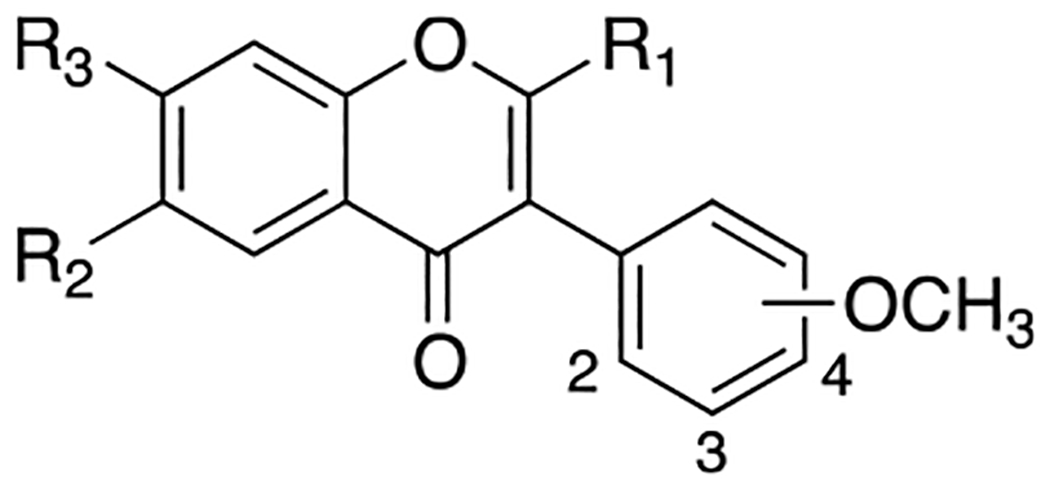						
Compd	R_1_	OCH_3_ position	R_2_	R_3_	FPR1-HL60 (IC_50_, μM)	FPR2-HL60 (IC_50_, μM)
**24a**	CH_3_	2	*n*-hexyl	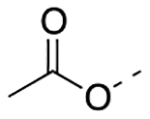	0.07 ± 0.025	N.A.^a^
**24b**	CH_3_	3	*n*-hexyl		0.17 ± 0.02	N.A.
**24c**	CH_3_	4	*n*-hexyl		0.8 ± 0.25	N.A.
**25a**	CF_3_	2	*n*-hexyl		0.025 ± 0.007	N.A.
**25b**	CF_3_	3	*n*-hexyl		0.16 ± 0.03	N.A.
**25c**	CF_3_	4	*n*-hexyl		0.49 ± 0.14	N.A.
**26a**	CH_3_	2	*n*-propyl		0.09 ± 0.03	N.A.
**26b**	CH_3_	3	*n*-propyl		0.15 ± 0.05	N.A.
**26c**	CH_3_	4	*n*-propyl		0.39 ± 0.13	N.A.
**27**	CH_3_	2	ethyl		1.2 ± 0.4	N.A.
**28**	CH_3_	2	*n*-butyl		0.8 ± 0.3	N.A.
**29**	CH_3_	2	*n*-pentyl		0.4 ± 0.1	N.A.
**30**	CH_3_	2	*n*-hexyl	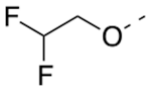	N.A.	N.A.
**35a**	CH_3_	2	*n*-hexyl	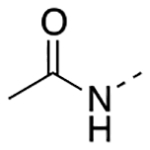	N.A.	N.A.
**35b**	CH_3_	3	*n*-hexyl		N.A.	N.A.
**35c**	CH_3_	4	*n*-hexyl		N.A.	N.A.
**36**	CH_3_	2	*n*-propyl		N.A.	N.A.
**37a**	CH_3_	2	methyl	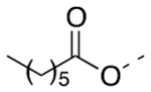	N.A.	N.A.
**37b**	CH_3_	3	methyl		21.9 ± 6.5	N.A.
**37c**	CH_3_	4	methyl		N.A.	N.A.
**38**	CH_3_	2	*n*-hexyl	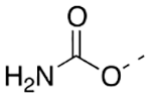	N.A.	N.A.
**39**	CH_3_	2	*n*-propyl		28.9 ± 7.6	N.A.

a Not Active.

**Table 2:** Cytotoxicity of compounds **24a** and **25a** in AGS and NCl–N87 cells.

	AGS EC_50_ (μM ± S.E.M.)			NCl–N87 EC_50_ (μM ± S.E.M.)		
**Compd**	**24 h**	**48 h**	**72 h**	**24 h**	**48 h**	**72 h**
**24a**	24.7 ± 4.0	10.97 ± 1.91	7.55 ± 1.75	32.9 ± 2.95	8.65 ± 0.65	11.5 ± 1.2
**25a**	16.7 ± 0.7	8.0 ± 0.1	5.45 ± 3.45	13.3 ± 1.88	10.2 ± 1.0	2.65 ± 0.50

## Data Availability

Data will be made available on request.

## Abbreviations:

ALOX: arachidonic acid lipoxygenase
ANGPT2: angiopoietin 2
*f*MLF: formyl-methionyl-leucyl-phenylalanine
GC: gastric cancer
FPRs: Formyl Peptide Receptors
GPCRs: G protein coupled receptors
SARs: structure-activity relationships
TM: transmembrane
VEGFA: vascular growth factor A
