# Supplementary material for: Development of potent isoflavone-based formyl peptide receptor 1 (FPR1) antagonists and their effects in gastric cancer cell models
Source: Eur J Med Chem. Author manuscript; Available in PMC 2024 Dec 5. (PMC10822168; doi:10.1016/j.ejmech.2023.115854)
Supplement: Supplementary Material [file NIHMS1960610-supplement-Supplementary_Material.pdf]

Supporting Information

Development of Potent Isoflavone-based Formyl Peptide Receptor 1 (FPR1) Antagonists and their Effects in Gastric Cancer Cell Models

Fabio Francavilla, Federica Sarcina, Igor A. Scheptkin, Lylia N. Kirpotina, Marialessandra Contino, Annalisa Schirizzi, Giampiero De Leonardis, Andrei Klebnikov, Rosalba D'Alessandro, Mark T. Quinn, Enza Lacivita,* Marcello Leopoldo

Content:

Elemental Analysis of Target Compounds.
Docking poses of compound **25a** superimposed with *f*MILF peptide
^{1}H NMR spectra of selected target compounds
HPLC traces of of compound **24a** and **25a**

Elemental Analysis

		calculated				found		
**compd**	**formula**	**C%**	**H%**	**N%**		**C%**	**H%**	**N%**
**24a**	$C_{25}H_{28}O_5$	73.51	6.91	--		73.36	6.61	--
**24b**	$C_{25}H_{28}O_5$	73.51	6.91	--		73.56	7.01	--
**24c**	$C_{25}H_{28}O_5$	73.51	6.91	--		73.37	6.76	--
**25a**	$C_{25}H_{25}F_3O_5$	64.93	5.45	--		64.96	5.78	--
**25b**	$C_{25}H_{25}F_3O_5$	64.93	5.45	--		64.59	5.75	--
**25c**	$C_{25}H_{25}F_3O_5$	64.93	5.45	--		65.32	5.36	--
**26a**	$C_{22}H_{22}O_5$	72.12	6.05	--		72.33	5.96	--
**26b**	$C_{22}H_{22}O_5$	72.12	6.05	--		72.47	6.02	--
**26c**	$C_{22}H_{22}O_5$	72.12	6.05	--		72.16	6.37	--
**27**	$C_{21}H_{20}O_5$	71.58	5.72	--		71.65	5.68	--
**28**	$C_{23}H_{24}O_5$	72.61	6.36	--		72.32	6.46	--
**29**	$C_{24}H_{26}O_5$	73.08	6.64	--		73.35	6.63	--
**30**	$C_{25}H_{28}F_2O_4$	69.75	6.56	--		69.78	6.52	--
**35a**	$C_{25}H_{29}NO_4$	73.68	7.17	3.44		73.32	6.95	3.40
**35b**	$C_{25}H_{29}NO_4$	73.68	7.17	3.44		73.78	7.36	3.18
**35c**	$C_{25}H_{29}NO_4$	73.68	7.17	3.44		73.45	6.97	3.23
**36**	$C_{22}H_{23}NO_4$	72.31	6.34	3.83		71.95	6.28	3.67
**37a**	$C_{25}H_{28}O_5$	73.51	6.91	--		73.56	6.76	--
**37b**	$C_{25}H_{28}O_5$	73.51	6.91	--		73.48	6.67	--
**37c**	$C_{25}H_{28}O_5$	73.51	6.91	--		73.33	6.58	--
**38**	$C_{26}H_{31}NO_5$	71.37	7.14	3.20		71.45	7.37	3.19
**39**	$C_{23}H_{25}NO_5$	69.86	6.37	3.54		69.55	6.24	3.43

Figure S1. The docking pose of compound **25a** (thin blue skeleton) superimposed on the experimental position (PDB: 7VFX) of fMILF peptide (thick red skeleton). Two projections of the superimposition are shown. Residues within 3 Å from the pose are visible.

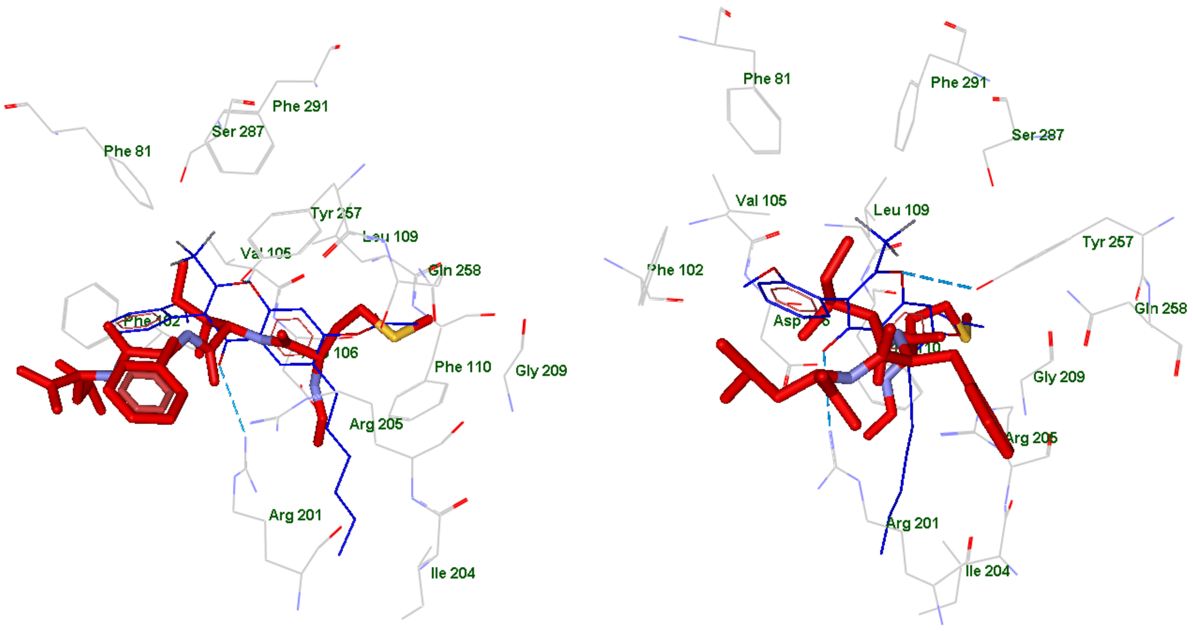

Figure S2. ^{1}H NMR spectra of selected target compounds.

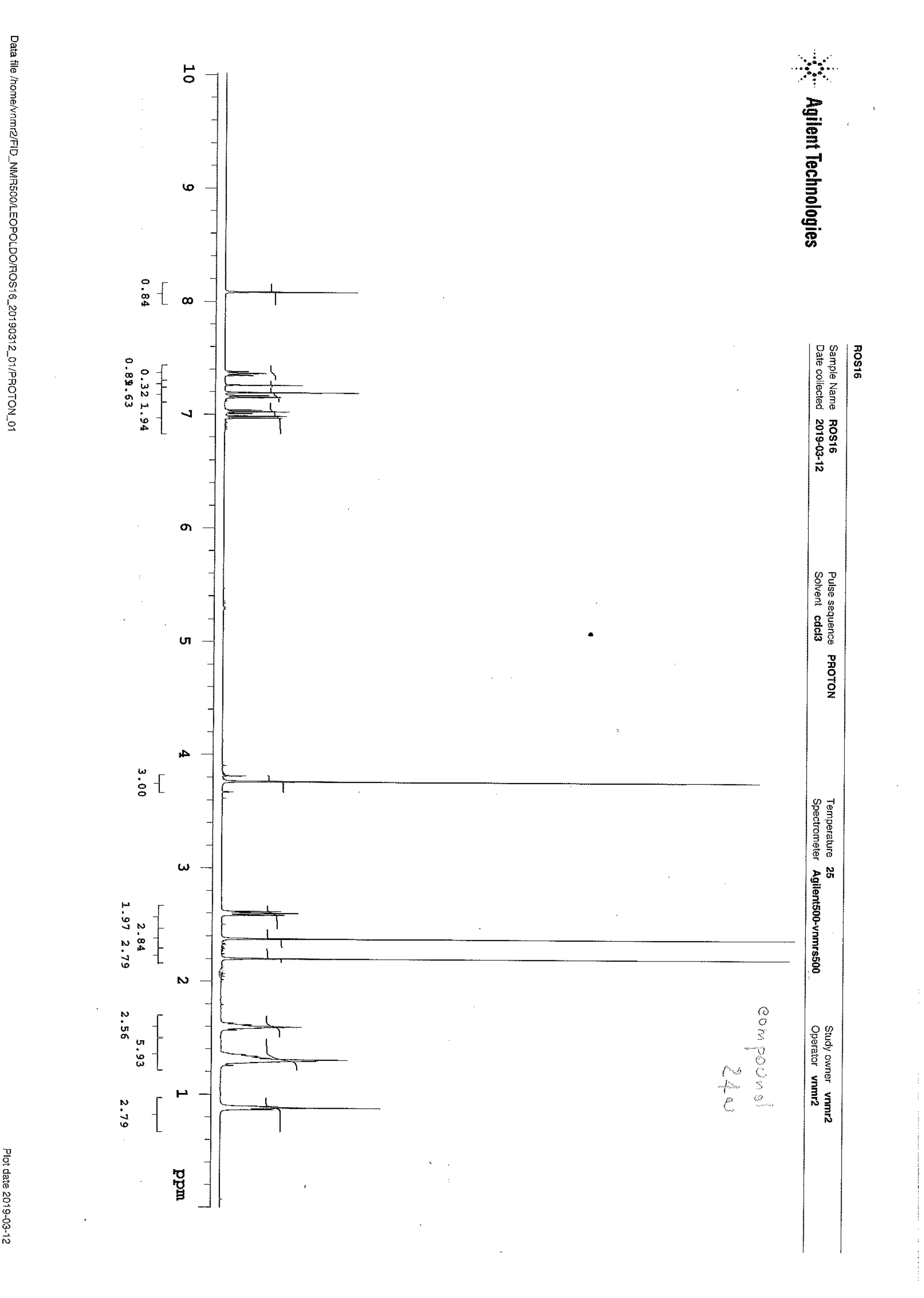

Agilent Technologies

ROS12

Sample Name	**ROS12**	Pulse sequence	**PROTON**	Temperature	**25**	Study owner	**vnmr2**
Date collected	**2019-03-12**	Solvent	**cdcl3**	Spectrometer	**Agilent500-vnmrs500**	Operator	**vnmr2**

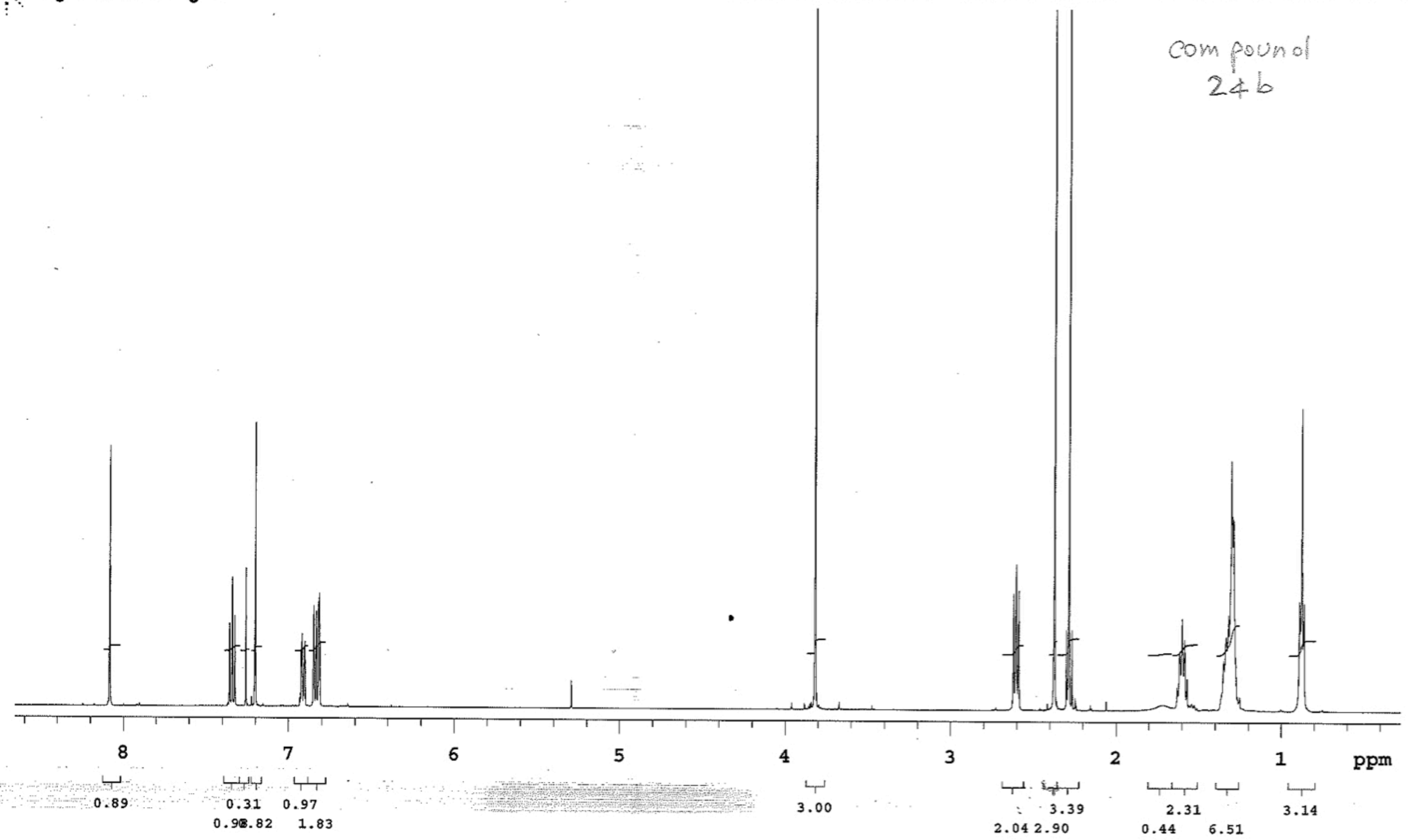

Data file /home/vnmr2/FID_NMR500/LEOPOLDO/ROS12_20190312_01/PROTON_01

Plot date 2019-03-12

Agilent Technologies

Sample Name	Pulse sequence **PROTON**	Temperature **25**	Study owner **vnmr2**
Date collected **2018-12-11**	Solvent **cdcl3**	Spectrometer **Mercury300-mercury300**	Operator **vnmr2**

com pound 24c

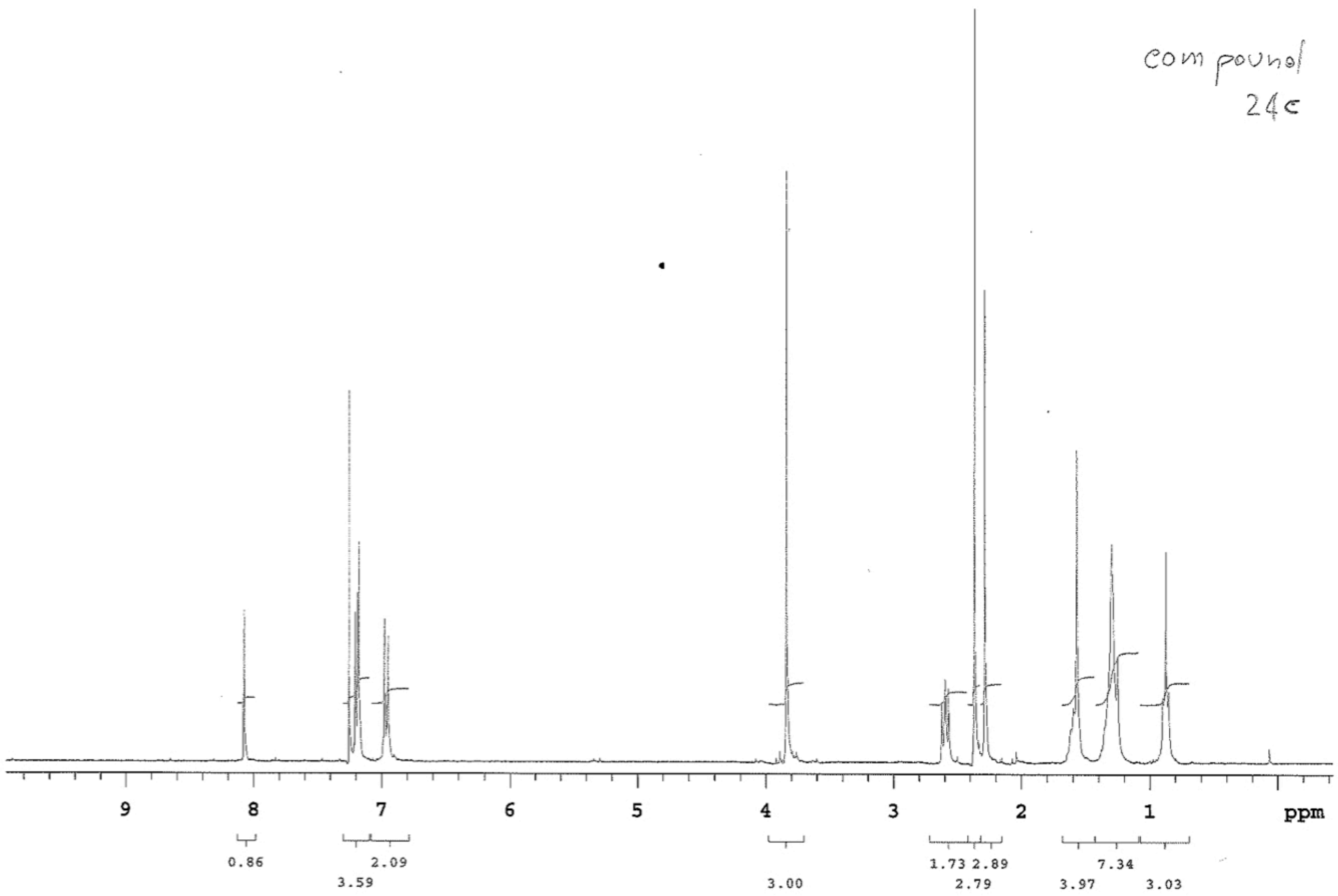

Data file exp

Plot date 2018-12-11

Agilent Technologies

Sample Name	Pulse sequence **PROTON**	Temperature **25**	Study owner **vnmr2**
Date collected **2019-02-27**	Solvent **cdcl3**	Spectrometer **Mercury300-mercury300**	Operator **vnmr2**

compound 25a

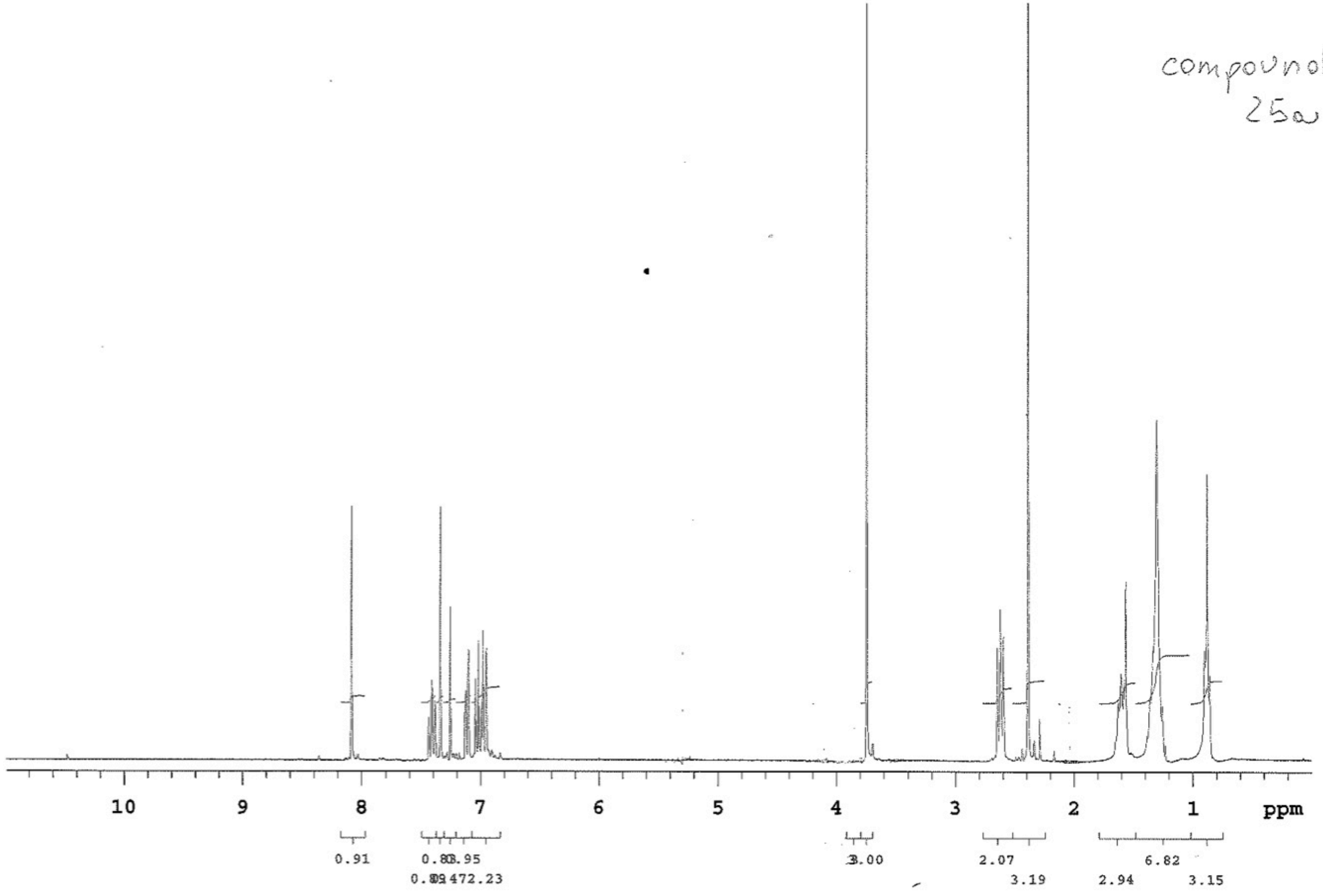

Data file exp

Plot date 2019-02-27

ROS24

Sample Name **ROS24**	Pulse sequence **PROTON**	Temperature **25**	Study owner **vnmr2**
Date collected **2019-03-12**	Solvent **cdcl3**	Spectrometer **Agilent500-vnmrs500**	Operator **vnmr2**

compound 25b

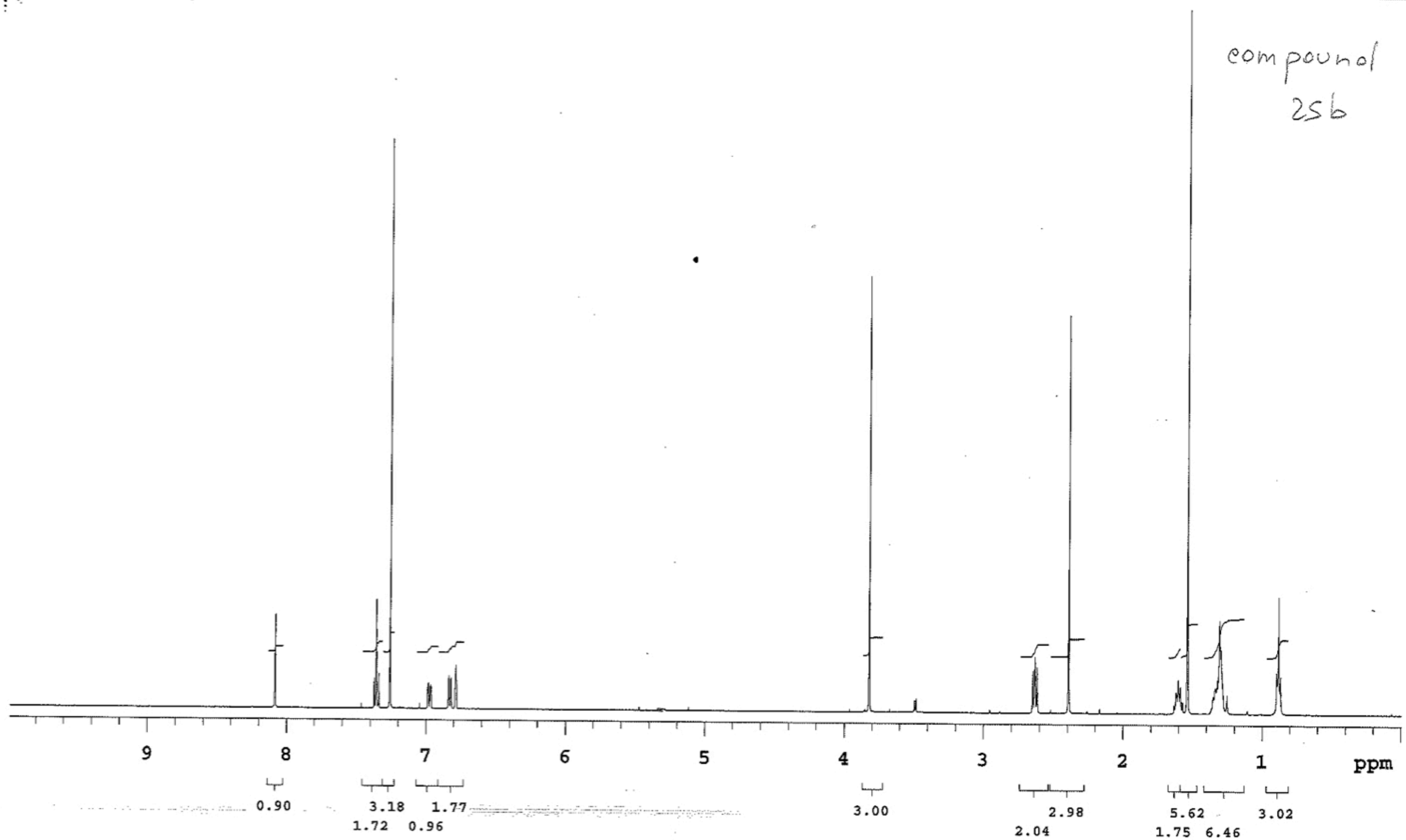

Data file /home/vnmr2/FID_NMR500/LEOPOLDO/ROS24_20190312_01/PROTON_01

Plot date 2019-03-12

Agilent Technologies

Sample Name
Date collected 2019-03-18

Pulse sequence PROTON
Solvent cdcl3

Temperature 25
Spectrometer Mercury300-mercury300

Study owner vnmr2
Operator vnmr2

compound 26a

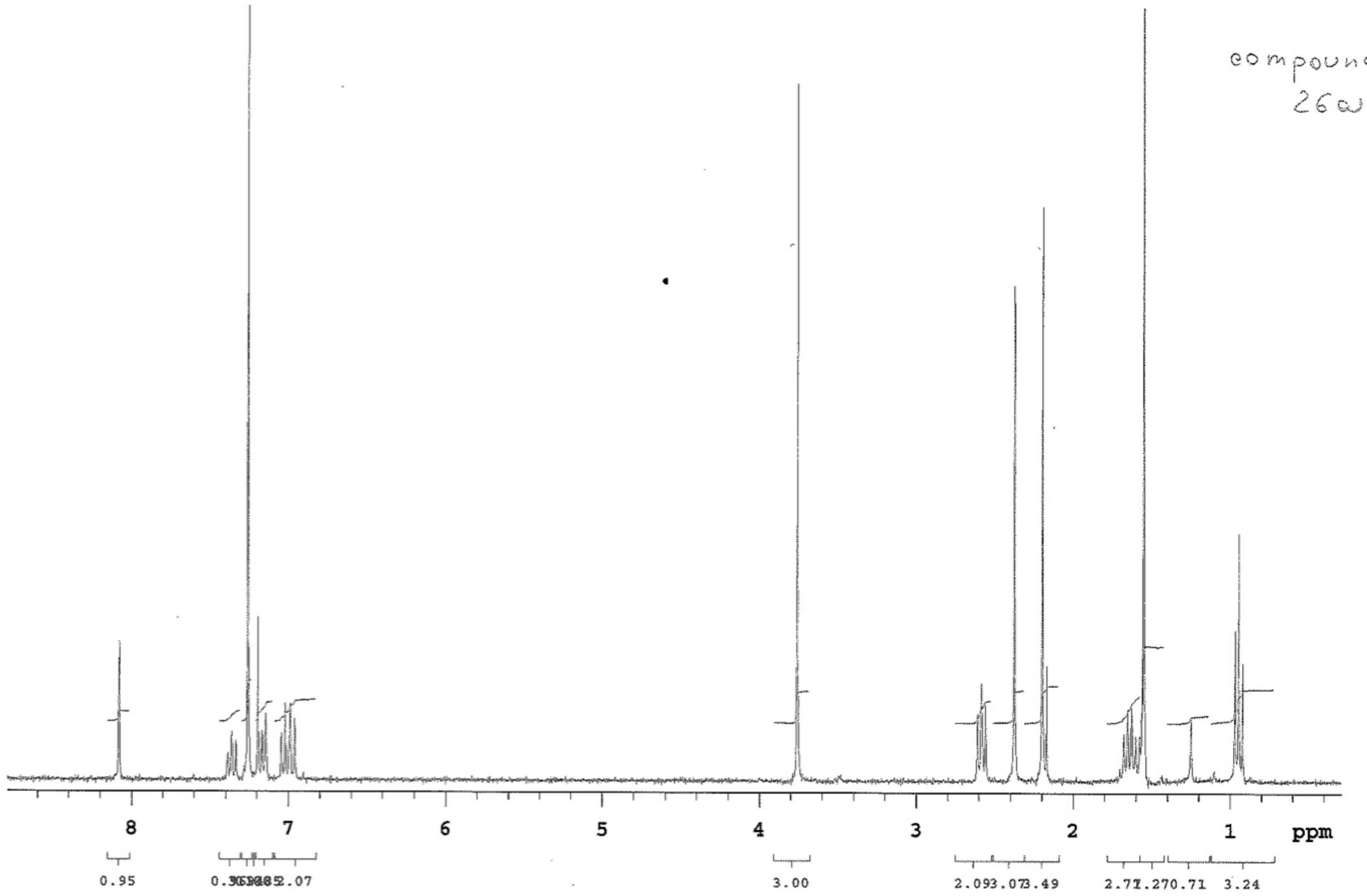

Data file exp

Plot date 2019-03-18

Agilent Technologies

Sample Name	Pulse sequence **PROTON**	Temperature **25**	Study owner **vnmr2**
Date collected **2019-03-20**	Solvent **cdcl3**	Spectrometer **Mercury300-mercury300**	Operator **vnmr2**

compound 26c

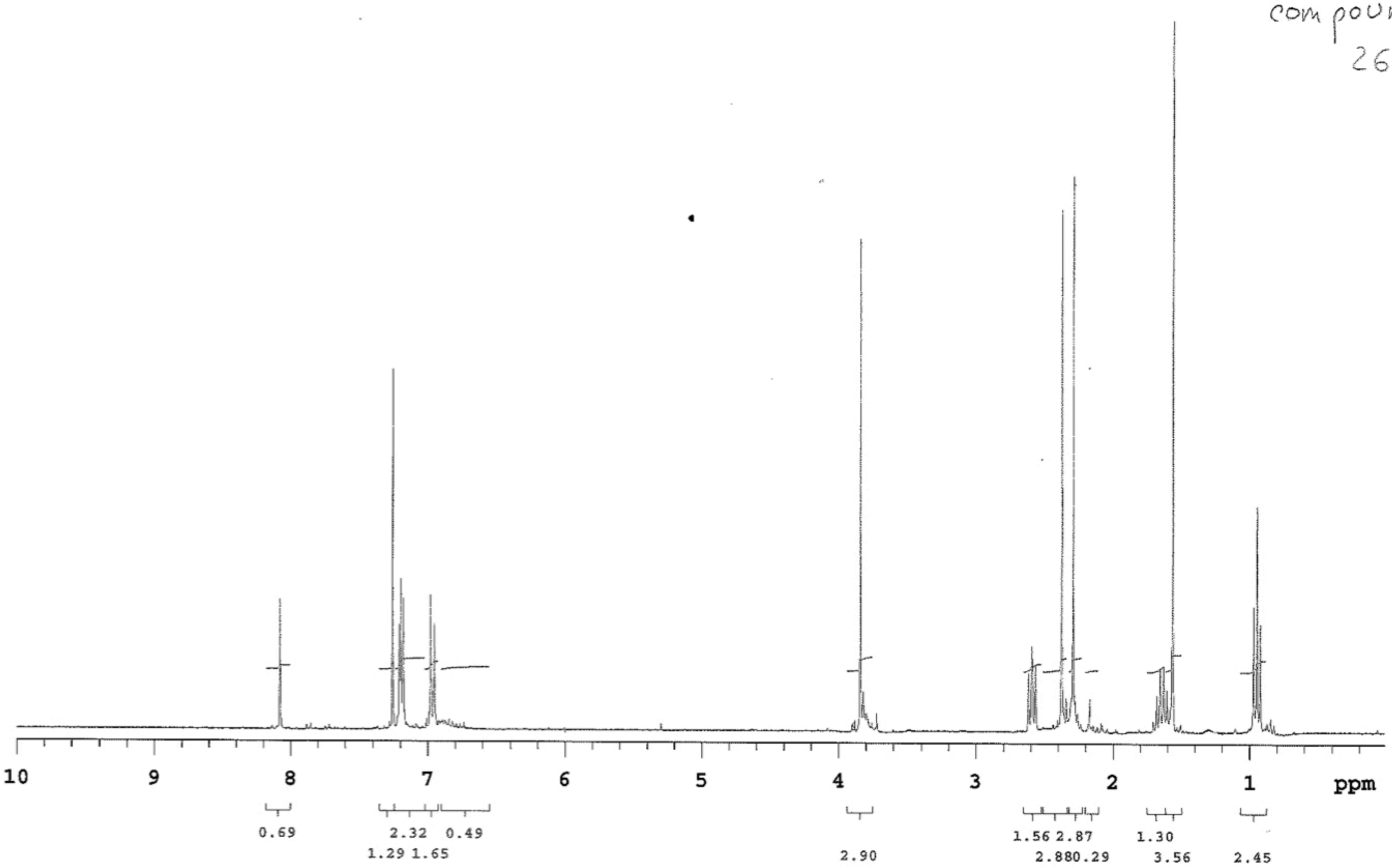

Data file exp

Plot date 2019-03-20

Agilent Technologies

Sample Name	Pulse sequence **PROTON**	Temperature **25**	Study owner **vnmr2**
Date collected **2019-11-18**	Solvent **cdcl3**	Spectrometer **Mercury300-mercury300**	Operator **vnmr2**

compound 27

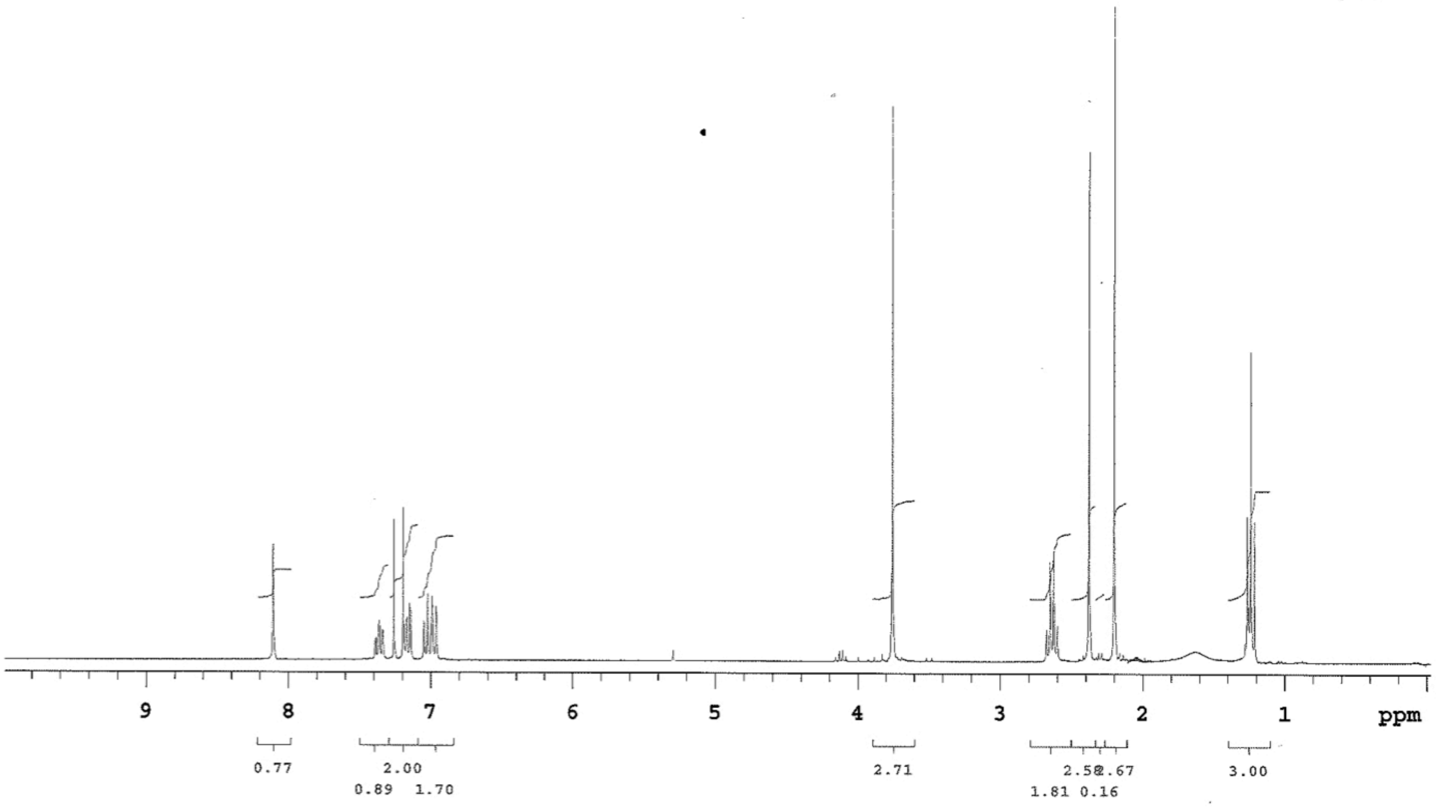

Data file exp

Plot date 2019-11-18

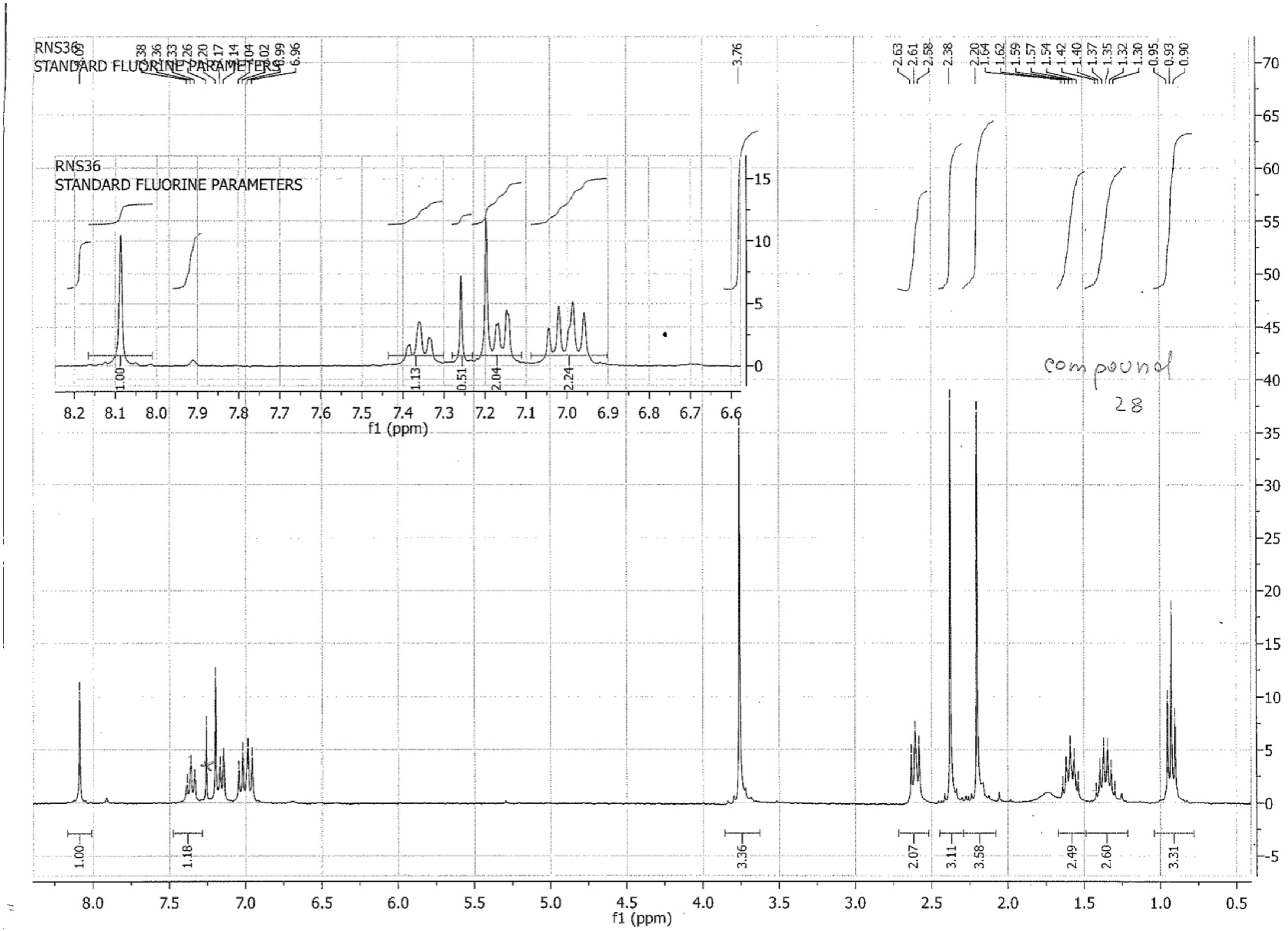

RNS36
STANDARD FLUORINE PARAMETERS
8.09
7.38
7.36
7.33
7.26
7.20
7.17
7.14
7.04
7.02
6.99
6.96
3.76
2.63
2.61
2.58
2.38
2.20
1.64
1.62
1.59
1.57
1.54
1.42
1.40
1.37
1.35
1.32
1.30
0.95
0.93
0.90
1.00
1.13
0.51
2.04
2.24
1.18
3.36
2.07
3.11
3.58
2.49
2.60
3.31
f1 (ppm)
compound 28

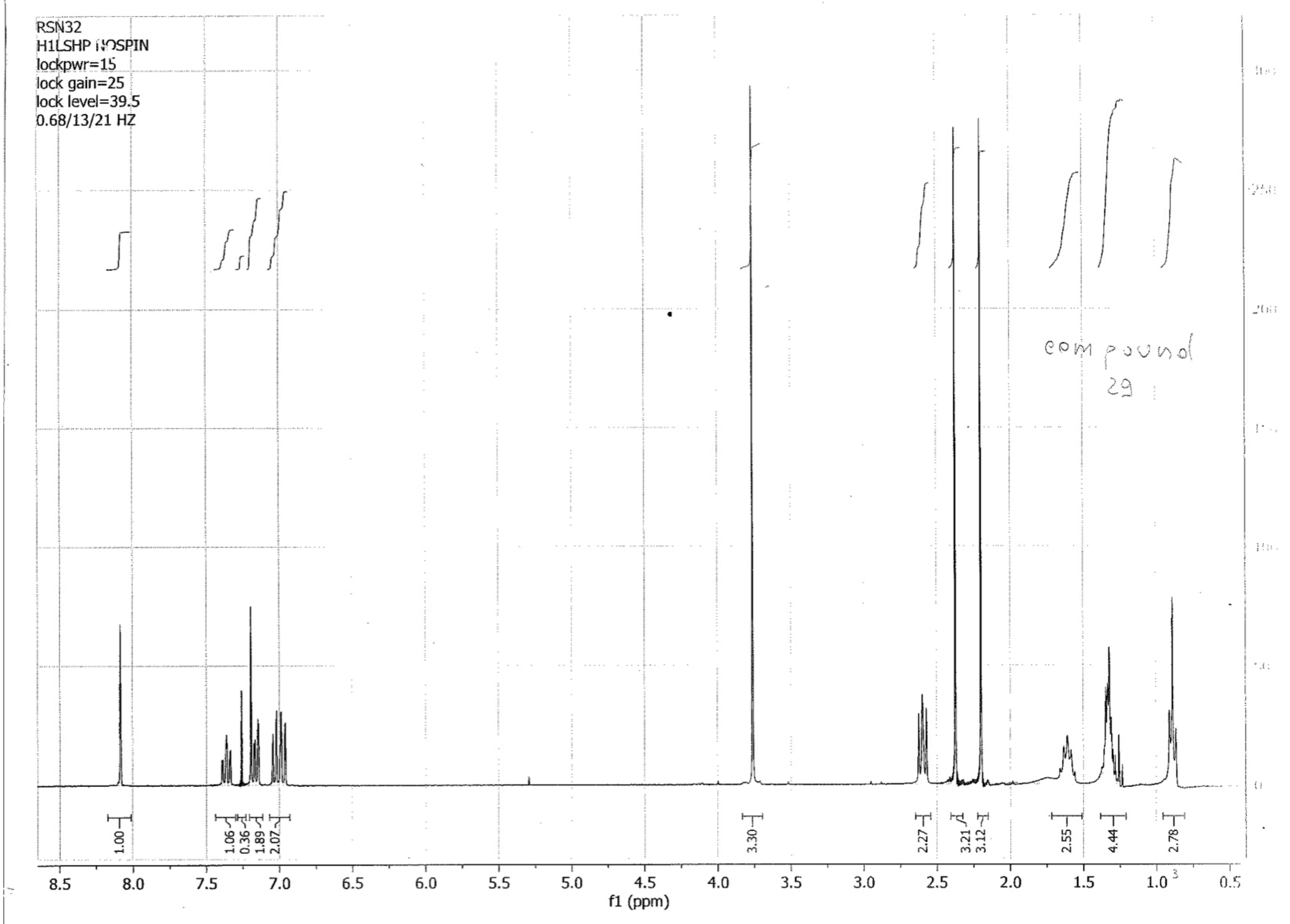
RSN32
H1LSHP NOSPIN
lockpwr=15
lock gain=25
lock level=39.5
0.68/13/21 HZ
1.00
1.06
0.36
1.89
2.07
3.30
2.27
3.21
3.12
2.55
4.44
2.78
8.5
8.0
7.5
7.0
6.5
6.0
5.5
5.0
4.5
4.0
3.5
3.0
2.5
2.0
1.5
1.0
0.5
f1 (ppm)
compound
29

Agilent Technologies

Sample Name		Pulse sequence	PROTON	Temperature	25	Study owner	vnmr2
Date collected	2019-12-11	Solvent	cdcl3	Spectrometer	Mercury300-mercury300	Operator	vnmr2

compound 30

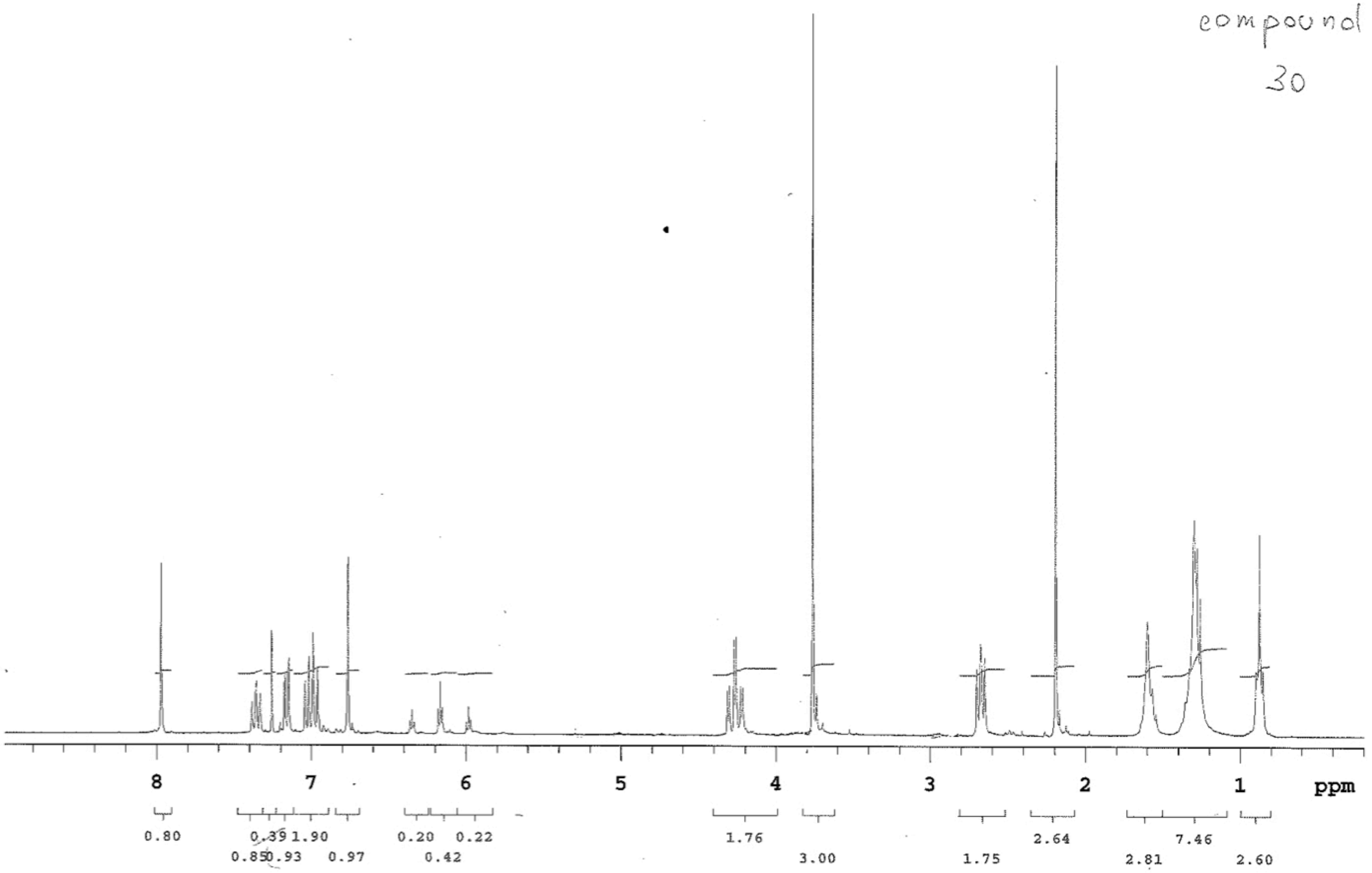

Data file exp

Plot date 2019-12-11

Agilent Technologies

RSN13

Sample Name **RSN13**	Pulse sequence **PROTON**	Temperature **25**	Study owner **vnmr2**
Date collected **2019-11-20**	Solvent **cdcl3**	Spectrometer **Agilent500-vnmrs500**	Operator **vnmr2**

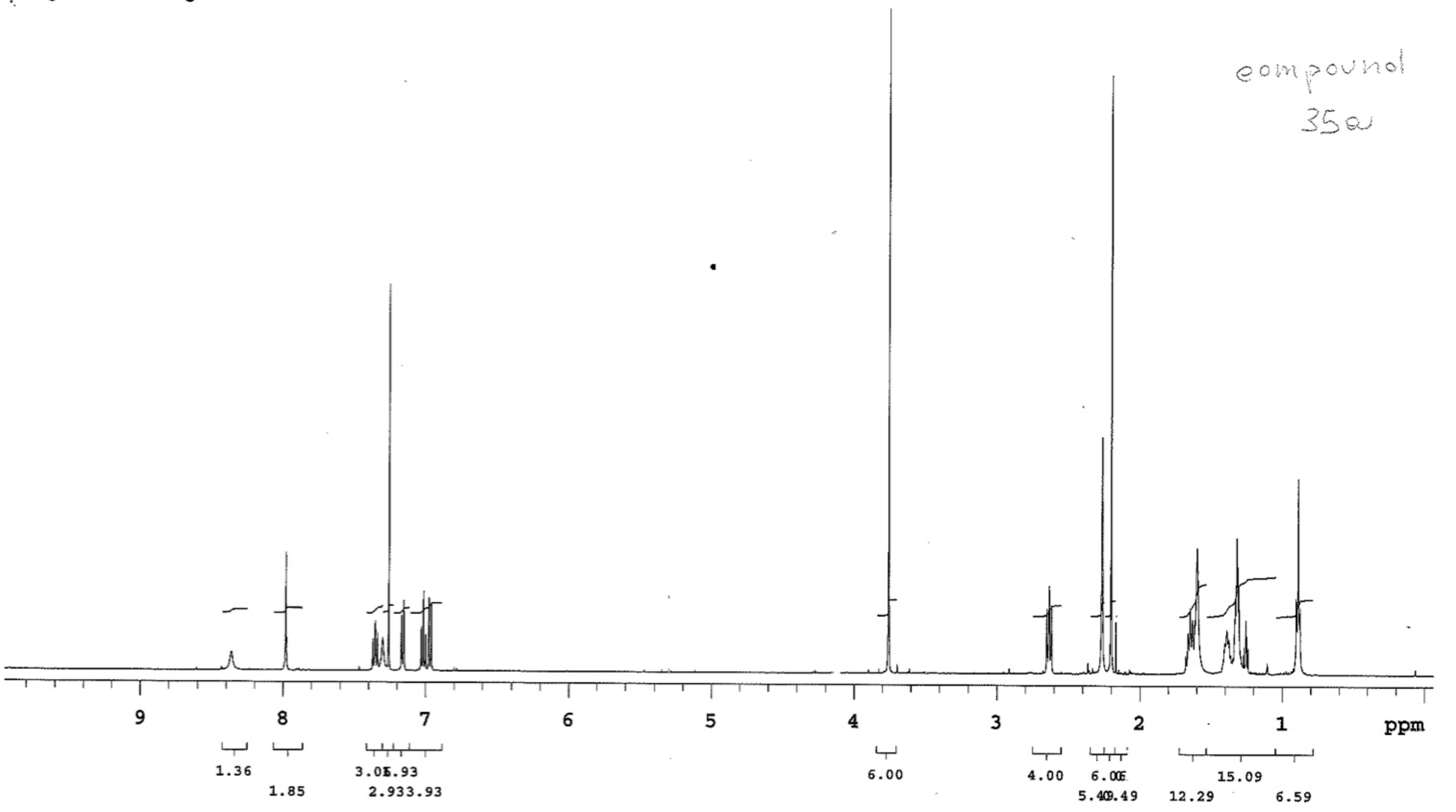

Data file /home/vnmr2/FID_NMR500/LEOPOLDO/RSN13_20191120_01/PROTON_01

Plot date 2019-11-20

Agilent Technologies

Sample Name	Pulse sequence **PROTON**	Temperature **25**	Study owner **vnmr2**
Date collected **2020-02-19**	Solvent **cdcl3**	Spectrometer **Mercury300-mercury300**	Operator **vnmr2**

compound 35C

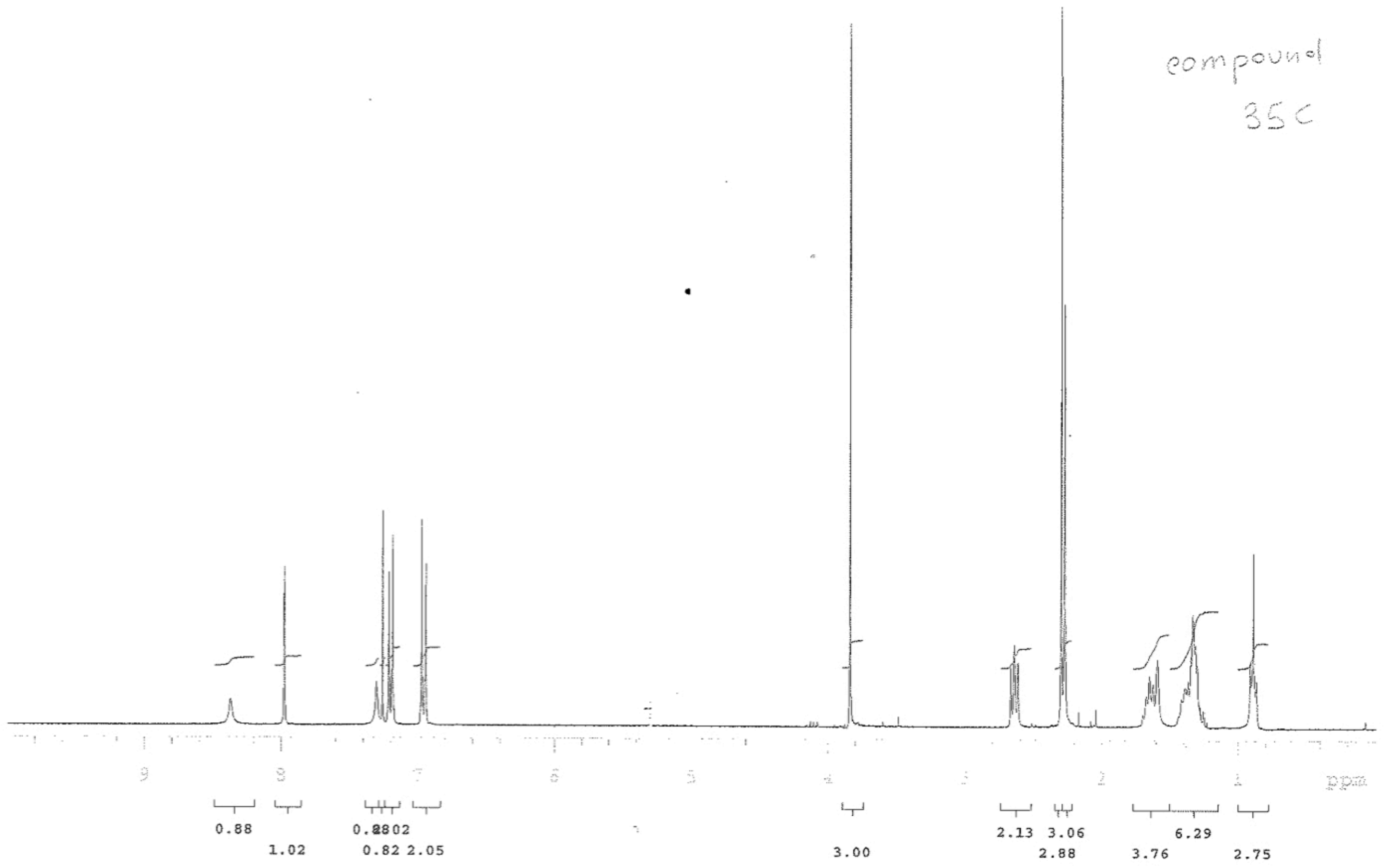

Data file exp

Plot date 2020-02-19

Agilent Technologies

RSN30A

Sample Name **RSN30A**	Pulse sequence **PROTON**	Temperature **25**	Study owner **vnmr2**
Date collected **2020-02-18**	Solvent **cdcl3**	Spectrometer **Agilent500-vnmrs500**	Operator **vnmr2**

compound 36

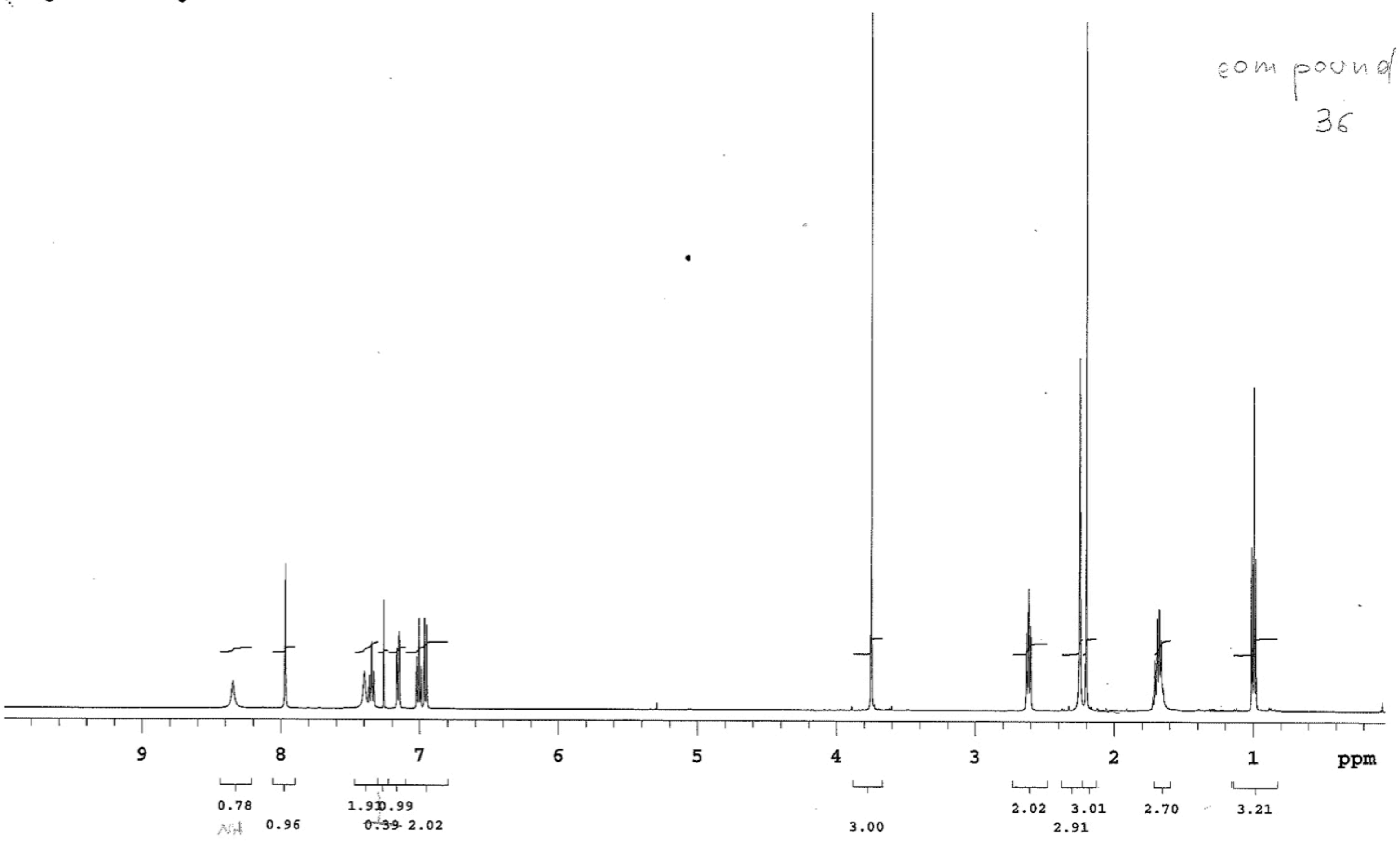

Data file /home/vnmr2/FID_NMR500/LEOPOLDO/RSN30A_20200218_01/PROTON_01

Plot date 2020-02-18

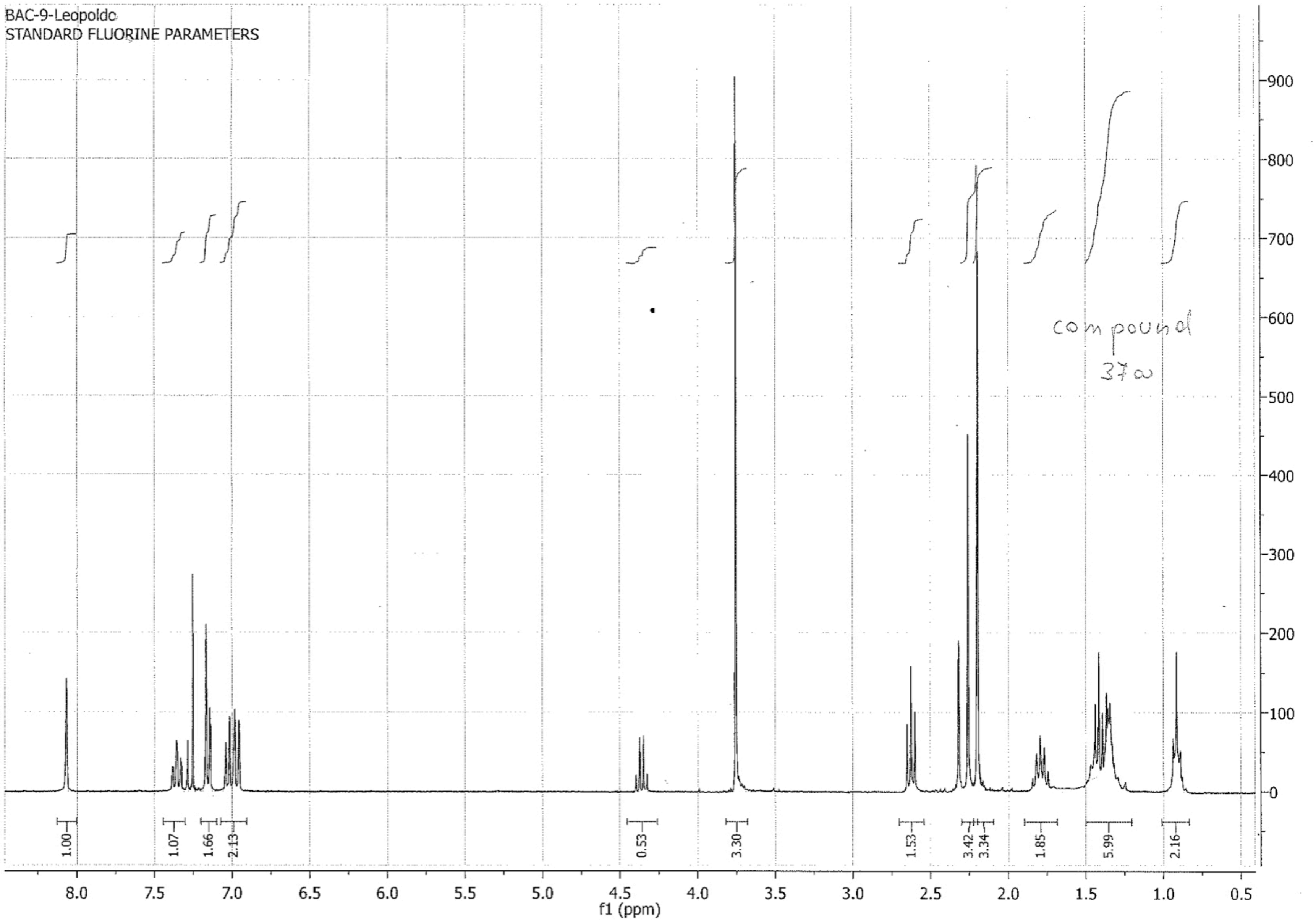
BAC-9-Leopoldo
STANDARD FLUORINE PARAMETERS
compound
37a
1.00
1.07
1.66
2.13
0.53
3.30
1.53
3.42
3.34
1.85
5.99
2.16
8.0
7.5
7.0
6.5
6.0
5.5
5.0
4.5
4.0
3.5
3.0
2.5
2.0
1.5
1.0
0.5
f1 (ppm)
900
800
700
600
500
400
300
200
100
0

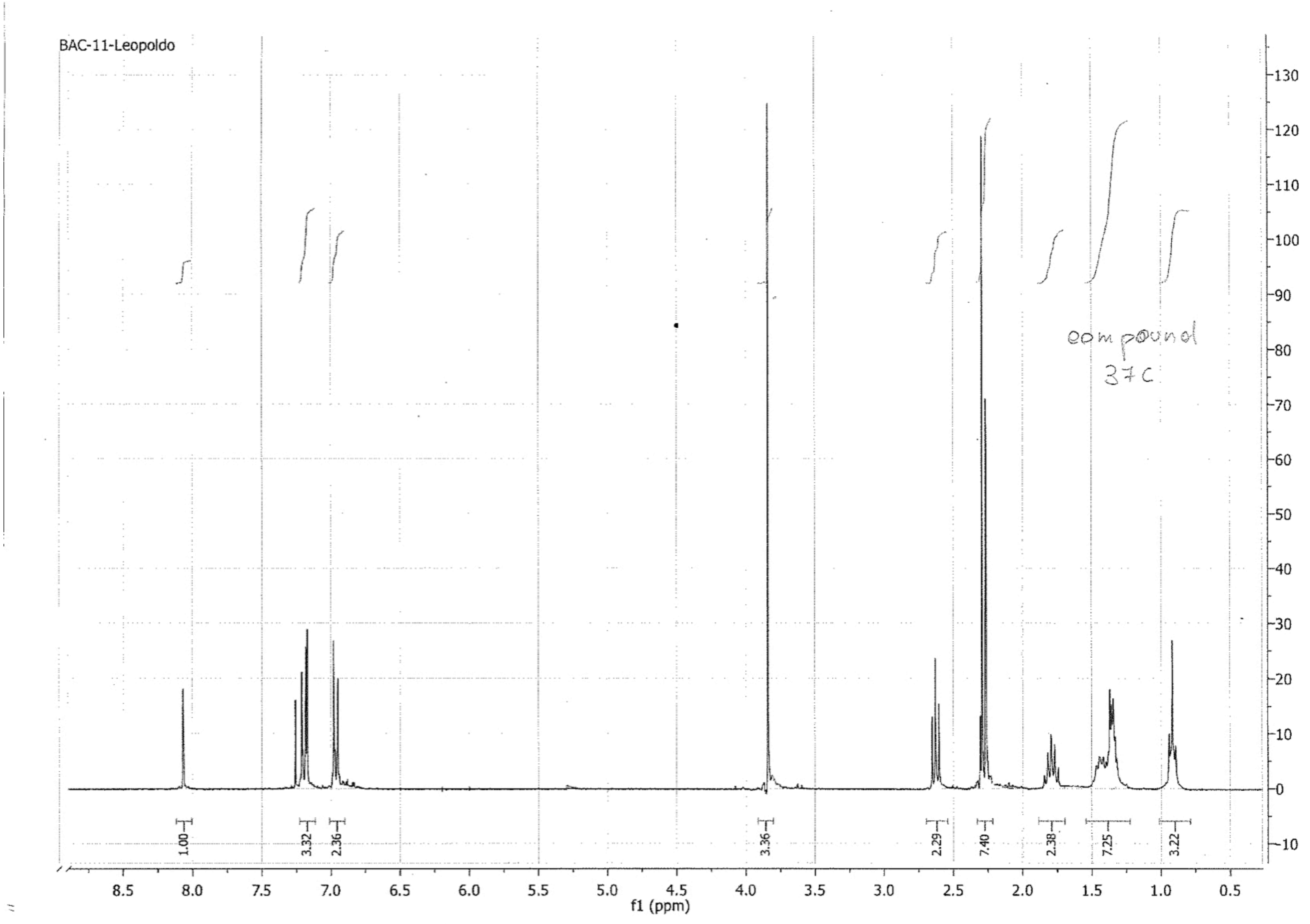
BAC-11-Leopoldo
compound
37C
1.00
3.32
2.36
3.36
2.29
7.40
2.38
7.25
3.22
8.5
8.0
7.5
7.0
6.5
6.0
5.5
5.0
4.5
4.0
3.5
3.0
2.5
2.0
1.5
1.0
0.5
f1 (ppm)
130
120
110
100
90
80
70
60
50
40
30
20
10
0
-10

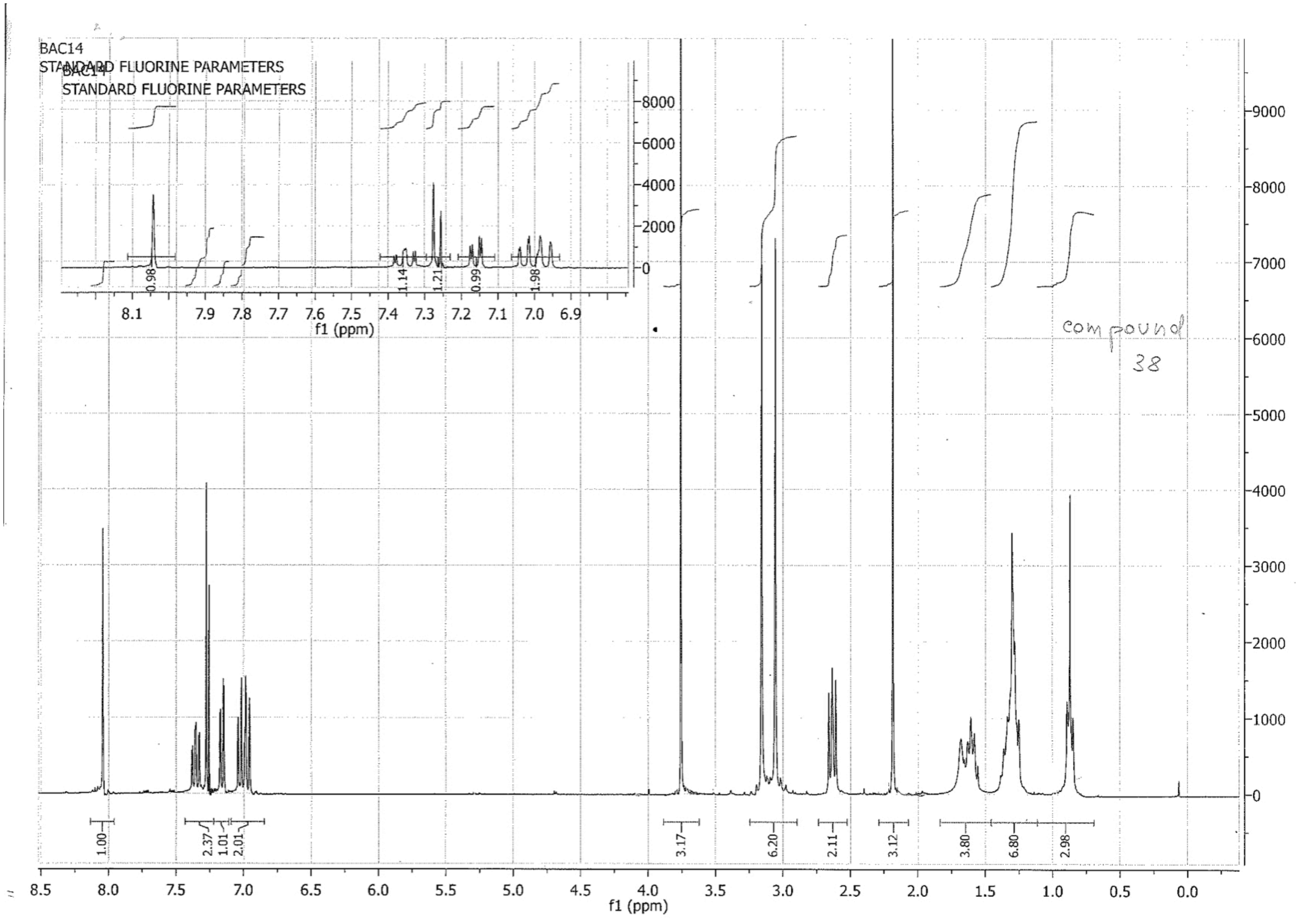

BAC14
STANDARD FLUORINE PARAMETERS
BAC14
STANDARD FLUORINE PARAMETERS
0.98
1.14
1.21
0.99
1.98
8.1 7.9 7.8 7.7 7.6 7.5 7.4 7.3 7.2 7.1 7.0 6.9
f1 (ppm)
0
2000
4000
6000
8000
compound 38
1.00
2.37
1.01
2.01
3.17
6.20
2.11
3.12
3.80
6.80
2.98
8.5 8.0 7.5 7.0 6.5 6.0 5.5 5.0 4.5 4.0 3.5 3.0 2.5 2.0 1.5 1.0 0.5 0.0
f1 (ppm)
0
1000
2000
3000
4000
5000
6000
7000
8000
9000

Figure S3. HPLC trace of compound **24a**.

```
Data File C:\CHEM32\1\DATA\EL\ROS\ROS_LC 2023-07-26 20-20-35\RNS10.D
Sample Name: RNS10
```

compound 24a

```
=====================================================================
Acq. Operator   :                               Seq. Line :   5
Acq. Instrument : HPLC PREP                      Location : Vial 5
Injection Date  : 26-Jul-23 21:46:31                   Inj :   1
                                               Inj Volume : 100.000 µl
Different Inj Volume from Sample Entry!  Actual Inj Volume : 5.000 µl
Method          : C:\CHEM32\1\DATA\EL\ROS\ROS_LC 2023-07-26 20-20-35\PX70_30.M (Sequence
                  Method)
Last changed    : 26-Jul-23 20:20:35
```

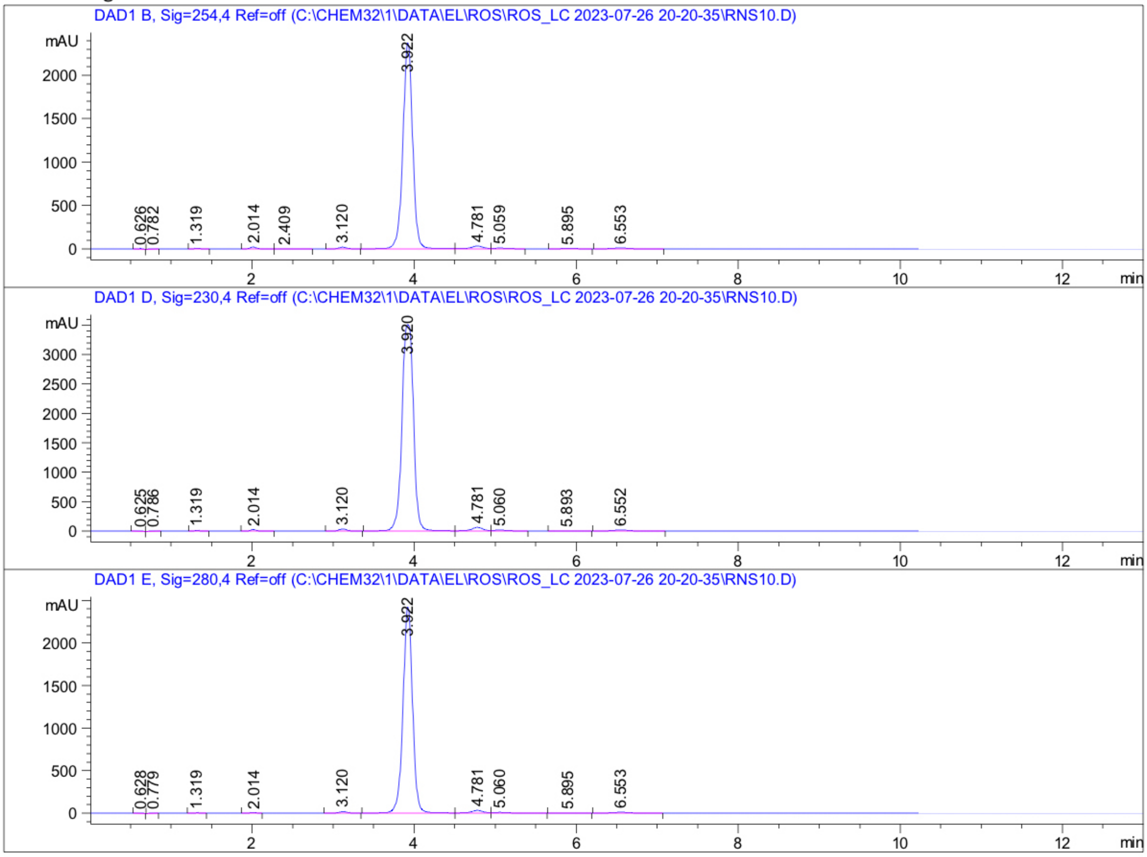


```
=================================================================================
                           Fraction Information
=================================================================================
Fraction collection off
=================================================================================
No Fractions found.
=================================================================================
```

Figure S6. HPLC trace of compound **25a**.

```
Data File C:\CHEM32\1\DATA\EL\ROS\ROS_LC 2023-07-26 20-20-35\RNS28.D
Sample Name: RNS28
```

compound 25a

```
=====================================================================
Acq. Operator   :                                Seq. Line :    4
Acq. Instrument : HPLC PREP                       Location : Vial 4
Injection Date  : 26-Jul-23 21:31:35                   Inj :    1
                                                Inj Volume : 100.000 µl
Different Inj Volume from Sample Entry!  Actual Inj Volume : 5.000 µl
Method          : C:\CHEM32\1\DATA\EL\ROS\ROS_LC 2023-07-26 20-20-35\PX70_30.M (Sequence
                  Method)
Last changed    : 26-Jul-23 20:20:35
```

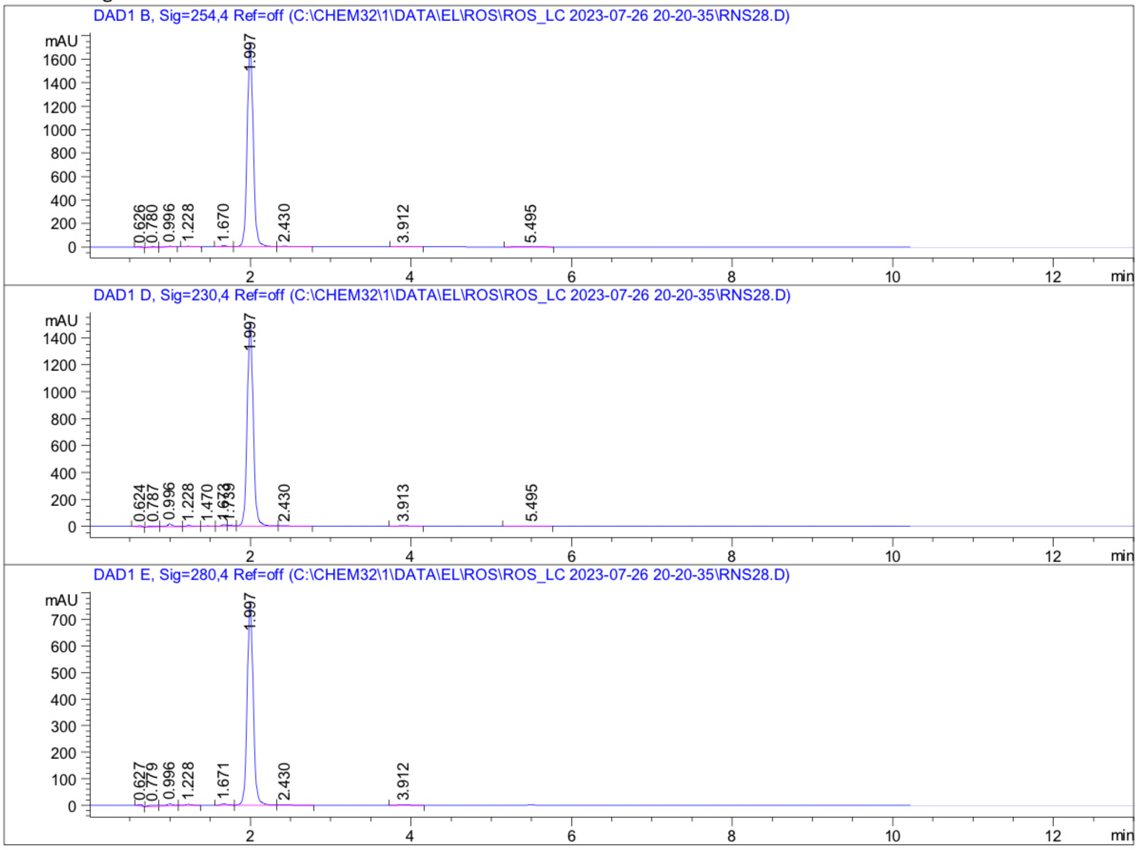


```
=================================================================================
                           Fraction Information
=================================================================================
Fraction collection off
=================================================================================
No Fractions found.
=================================================================================
```
